# HIF-1α alleviates ferroptosis in ulcerative colitis by regulation of GPX4

**DOI:** 10.1038/s41419-025-07883-8

**Published:** 2025-07-22

**Authors:** Weitao Hu, Yanliang Cai, Daxing Cai, Zongchi Chen, Siying Huang, Su Zhang, Huie Zhuang, Taiyong Fang, Xiaoqing Chen

**Affiliations:** 1 Department of Gastroenterology, The Second Affiliated Hospital of Fujian Medical University, Quanzhou, Fujian PR China; 2 Department of Pediatrics, The Second Affiliated Hospital of Fujian Medical University, Quanzhou, Fujian PR China; 3 Department of Rheumatology/Department of General Practice, The Second Affiliated Hospital of Fujian Medical University, Quanzhou, Fujian PR China

**Keywords:** Cell death, Cell signalling

## Abstract

Ferroptosis is an iron-dependent form of regulated cell death induced by the lethal accumulation of lipid peroxidation, while the precise mechanism of ferroptosis in the pathogenesis of ulcerative colitis (UC) remains to be elucidated. This study aimed to explore the potential effect of hypoxia inducible factor-1α (HIF-1α) on ferroptosis in intestinal epithelial cells (IECs) in UC. The relationship between ferroptosis and HIF-1α was initially investigated using clinical UC colon samples. In vitro and in vivo models of acute intestinal inflammatory response were constructed using lipopolysaccharide (LPS) and dextran sulfate sodium (DSS), respectively. The effect of HIF-1α on ferroptosis in UC was determined by establishing HIF-1α overexpression (HIF1A-OE) or knockdown (shHIF1A) IEC lines, and the mechanism by which HIF-1α mediated the transcription of glutathione peroxidase 4 (GPX4) was explored by combining Co-immunoprecipitation (Co-IP) and Chromatin immunoprecipitation-qPCR (ChIP-qPCR). The results indicated that ferroptosis was present in IECs from UC patients and colitis mice. Elevated expression of HIF-1α ameliorated the secretion of inflammatory cytokines and ferroptosis in IECs in vitro. HIF-1α inhibited ferroptosis by transcriptional activation of the *GPX4* gene in inflammatory IECs. HIF-1α ameliorated the general conditions of mice and intestinal barrier dysfunction and by suppressing ferroptosis in IECs of mice through upregulating the expression of GPX4. In conclusion, ferroptosis occurred in the IECs of UC patients and colitis mice. HIF-1α may improve UC by suppressing ferroptosis in IECs through regulating the transcription of GPX4.

## Introduction

Inflammatory bowel disease (IBD) refers to a group of chronic, non-specific inflammatory diseases of the intestinal tract whose etiology remains unclear, mainly including Crohn’s disease (CD) and ulcerative colitis (UC) [1]. Genetic background, environmental and intraluminal factors, and mucosal immune dysregulation have been suggested to be involved in UC pathogenesis [2]. Recent studies have emphasized the critical role of ferroptosis in the pathogenesis of UC [3].

Ferroptosis is a metabolism-related regulatory cell death (RCD) induced by iron-dependent lipid peroxidation [4]. The main features of ferroptosis are mitochondrial atrophy, lipid peroxidation, iron accumulation, glutathione consumption, and GPX4 inactivation, among others [5]. Mounting evidence suggests that ferroptosis is involved in a wide range of systemic diseases, including neurological disorders, ischemia/reperfusion injury, kidney failure, heart disease, and cancer [6–8]. Recent research has shown that ferroptosis is associated with intestinal diseases. Intestinal epithelial cells (IECs) from CD patients show signs of impaired glutathione peroxidase 4 (GPX4) activity and lipid peroxidation [9]. The main characteristics of ferroptosis can be observed in the colonic tissue of UC patients or colitis mouse models, whereas some effective ferroptosis regulators (ferrostatin-1, liproxstatin-1, etc.) can resist lipid oxidation and promote the repair of UC intestinal damage [10–12]. Current evidence suggests that ferroptosis is a negative regulator of UC, and inhibition of ferroptosis is expected to be a new approach for UC treatment; however, the potential molecular mechanisms remain undefined.

Hypoxia inducible factor-1α (HIF-1α), a transcription factor that regulates various important biological functions, has been shown to play a significant role in the maintenance of the intestinal barrier and immune response [13]. It has been shown in previous studies that the absence of HIF-1α in IECs can lead to colitis [14, 15], while the above perspective remains controversial. Intracellular HIF-1α activation was also proved to effectively facilitate the progression of colitis [16, 17]. Therefore, it remains to be further clarified what role HIF-1α actually plays in the progression of UC.

The available evidence suggested that HIF-1α mediated ferroptosis differently in various diseases or cells. HIF-1α was a negative regulator of Erastin- or RSL3-induced ferroptosis in human fibrosarcoma cells HT1080 and non-small cell lung cancer cells Calu-1 [18]. However, it has been reported that PER1/HIF-1α can promote ferroptosis and inhibit the tumor progression of oral squamous cell carcinoma [19]. Given the situation that research of HIF-1α on the ferroptosis of UC remains understudied and the working mechanism has yet to be clarified, we aimed to determine whether HIF-1α affects UC by regulating ferroptosis.

In the present study, we hypothesize that HIF-1α participates in the ferroptosis of UC. We provide evidence that HIF-1α was elevated in acute colitis, and HIF-1α might suppress inflammation and ferroptosis in UC. Specifically, HIF-1α regulated the transcription of GPX4 in colonic epithelial cells to inhibit ferroptosis, suggesting that ferroptosis and HIF-1α might act as an optimal target for the prevention and treatment of UC.

## Materials and methods

### Reagents

The primary antibodies were listed as follows: For Western blot: HIF-1α (1:1000, ab179483, abcam), FTH (1:1000, ab183781, abcam), FTL (1:10000, ab69090, abcam), zonulin (1:1000, ab256454, abcam), ZO-1 (1:1000, ab276131, abcam), Occludin (1:1000, ab216327, abcam), GPX4 (1:1000, ab125066, abcam), ACSL4 (1:10000, ab155282, abcam), SLC7A11 (1:1000, ab175186, abcam), LPCAT3 (5 µg/ml, ab239585, abcam), TXNRD1 (1:5000, ab124954, abcam), β-actin (1:1000, ab8226, abcam). For immunohistochemistry and immunofluorescence staining: 4-HNE (1:1000, ab48506, abcam), GPX4 (1:100, ab125066, abcam), ACSL4 (1:200, ab155282, abcam), SLC7A11 (1:500, ab175186, abcam), LPCAT3 (10 µg/ml, ab239585, abcam), TXNRD1 (1:200, ab124954, abcam), HIF-1α (1:200, AF1009, Affinity), CK18 (10 µg/ml, ab668, abcam), FTH (1:400, ab183781, abcam), MPO (1:1000, ab208670, abcam), ZO-1 (1:200, ab276131, abcam), Occludin (1:200, ab216327, abcam), For flow cytometry: C11-BODIPY^TM^ 581/591 (D3861, Invitrogen); PE-conjugated anti-E-Cadherin (1 ug/ml, 567052, BD Biosciences); FITC-conjugated anti-EpCAM (1 ug/ml, 11-5791-82, eBioscience).

### Patient specimens

Clinical fresh colonic mucosal specimens from 8 patients with active UC (Mayo endoscopic score ≥ 2) and 8 healthy controls, and paraffin-embedded colonic mucosal specimens from 30 patients with active UC (Mayo endoscopic score ≥ 2) and 20 healthy controls were obtained from The Second Affiliated Hospital of Fujian Medical University. The pathologies of the tissues were confirmed by experienced pathologists. The study was approved by the Institutional Ethics Committee of Fujian Medical University (approval No. 2023–488) and was performed according to the principles of the Declaration of Helsinki. All participants provided informed written consent.

### Cell culture and lentivirus infection

The human normal colon epithelial cell (NCM460) and HEK 293T cell were acquired from ATCC (Manassas, USA). All cells were authenticated by STR analysis and tested negative for mycoplasma contamination. The cells were grown in RPMI-1640 medium supplemented with 10% fetal bovine serum (Invitrogen, USA) and 1% penicillin-streptomycin (Gibco, USA) at 37 °C in a 5% CO_2_ atmosphere. The medium was replaced during incubation based on the cellular demand. To induce cellular inflammation, cells were treated with 10 μg/ml lipopolysaccharide (LPS, # HY-D1056, MCE) for 24 h after plating. Cell culture dishes and conical-bottom centrifuge tubes were purchased from Bioland (China).

For cell infection experiment, HIF-1A short hairpin RNA (sh-RNA) lentivirus and overexpression lentivirus manufactured by Genechem Co., LTD (Shanghai, China) were infected into NCM460 cells in the presence of 5 μg/ml polybrene with 10 multiples of infection (MOI). After infection for 16 h, the medium containing virus particles was removed and changed to complete medium. Three days post-infection, YFP expression was observed in three randomly selected fields using a fluorescence microscope. Approximately 90% incubated cells observed YFP staining were considered to be feasible for the following procedure. Optimal concentration of puromycin (Sigma, St. Louis, MO, USA) was confirmed in preliminary experiment, and the final concentration was determined as 4 μg/ml. Infection efficiency was guaranteed by RT-qPCR and Western blot. The sequences of sh-RNA were listed in Table S1.

### Immunohistochemistry (IHC) and Hematoxylin-eosin (H&E) staining

Immunohistochemical staining was conducted on formalin-fixed paraffin-embedded colonic specimens. Antigen retrieval was performed in 10 mM citrate buffer (pH = 6.0) for 10 min after deparaffinization. Tissues were incubated with the primary antibodies overnight at 4 °C. Subsequently, HRP-labeled broad-spectrum secondary antibody was applied for 30 min at room temperature. After peroxidase substrate DAB staining, slices were counterstained with hematoxylin for 3 min, and final images were captured by inverted microscope. The histopathological changes of UC tissues were assessed by HE staining. Tissues were fixed in 4% paraformaldehyde. After being dehydrated and embedded in paraffin, 4 µm sections were stained with hematoxylin and eosin. Colon tissue pathology assessment was scored according to the degree of damage to the mucosal layer of the colon and the degree of inflammatory cell infiltration in the lamina propria [20].

### RNA extraction and RT-qPCR

Total RNA was isolated using TRIzol® Reagent (Life Technologies, USA) according to manufacturers’ instructions. Extracted RNA was transcribed into cDNA using the PrimeScript™ RT reagent kit with gDNA eraser (Takara, Japan), and RT-qPCR was performed using the 7500 Real-Time PCR detection system (Applied Biosystems, USA). Relative mRNA expression was calculated using 2^−ΔΔCt^ method and normalized to β-actin expression. The primer sequences for RT-qPCR were listed in Table S2.

### Western blot

Western blot was performed as previously described. Tissue and cell lysates were prepared using RIPA lysis buffer (Beyotime, China). The samples were resolved by SDS-polyacrylamide gel electrophoresis and blotted on PVDF membranes (Millipore, USA). The membranes were blocked with a 5% nonfat milk solution in TBST for 2 h at room temperature. Primary antibodies were incubated at 4 °C overnight. The membranes were incubated with an HRP-conjugated secondary antibody at room temperature for 1 h. The immunoblots were visualized utilizing ImageQuant LAS 4000 (GE Healthcare, UK) with β-actin serving as an endogenous control, and the intensity of the western bands was quantified by ImageJ software (V1.8.0.112, NIH, Madison, WI, USA).

### Intestinal permeability assay

Intestinal epithelial integrity was assessed by FITC-dextran permeability assay and transepithelial electrical resistance (TEER). Passive diffusion of 4-kDa FITC-dextran (Sigma-Aldrich) was added at a concentration of 5 μM to the apical chamber in 100 μl of PBS, while the basolateral chamber contained 500 μl of PBS. The experiments were carried out under normal cell culture conditions at 37 °C, 5% CO_2_. Diffusion from the apical to basolateral side was measured by fluorescence reading in PBS on the basolateral side of the transwell system using a fluorescence microplate reader (GloMax® Discover Microplate Reader, Promega). Fluorescence reading was normalized to the control.

TEER of the NCM460 cell layers was measured on the first and 14th days of culture using an EVOM epithelial Volt/Ohm meter (World Precision Instruments Inc., Sarasota, FL, USA). Since the TEER was affected by the pore size and density of the insert membranes, the measurement procedure included measuring the blank resistance of the membrane alone (without cells) and measuring the resistance across the cell layer on the insert membrane. TEER values were typically reported in units of Ω·cm^2^ and calculated as follows: TEER value = (mean of the resistance of each well − mean of the resistance of blank) × membrane area.

### Lipid peroxidant assessment and iron assay

Malondialdehyde (MDA) was measured using a Malondialdehyde Content Test Kit (BC0025, Solarbio, China). After preparing the working solution and treating the cells according to the manufacturer’s instructions, the absorbance of each sample was measured at 532 nm and 600 nm, and the MDA content was calculated as described in the manual.

Lipid reactive oxygen species (ROS) level was detected by C11-BODIPY (581/591) (Invitrogen) staining. After intervention, cells were washed with PBS and incubated with 10 μM C11-BODIPY for 30 min. For flow cytometry, excess dye was removed, and labeled cells were trypsinised, resuspended in PBS with 5% FBS. The fluorescence was analyzed using a flow cytometer (Agilent, Carpinteria, CA). Additionally, cells were also observed with confocal laser microscopy (Nikon C1 Laser Scanning Confocal) after staining with DAPI.

The iron concentration was measured using an iron assay kit (#ab83366, abcam) as the manufacturer’s instructions described. Cells were homogenized in iron assay buffer and centrifuged at 10,000 × *g* for 10 min to remove insoluble materials. The supernatants were added to 96-well plate and adjusted to the volume of 100 μl with assay buffer. After incubation at 37 °C for 30 min, 100 μl iron probe was added and the reaction was incubated at 37 °C for 60 min. The absorbance at 593 nm was measured on a colorimetric microplate reader.

### Transmission electron microscopy (TEM)

TEM was performed on isolated intestinal segments to visualize the mitochondrial damage. Selected samples were prepared by rinsing in 0.1 M phosphate buffer and fixed with a mixture of 2% paraformaldehyde and 2.5% glutaraldehyde overnight. Subsequently, tissues and cells were dehydrated and embedded in resin according to standard procedures. Embedded samples were analyzed by a JEOL 1010 electron microscope (Tokyo, Japan). The level of ultrastructural damage to mitochondria was assessed using the Flameng scoring method [21].

### Immunofluorescence staining

Paraffin tissue slices were deparaffinized and rehydrated, and antigens were retrieved in citrate buffer using a pressure boiler for 10 min under a slightly boiling state. For paraformaldehyde-fixed cell climbing slides samples, cells were permeabilized by 0.5% Triton X-100 for 5 min and blocked by goat serum for 2 h. Tissue samples or cell slide samples were incubated with primary antibody at 4 °C overnight, followed by the HRP-conjugated secondary antibodies and fluorescent CF 488 or 549 Tyramide, AF 647 Tyramide, and Cy3 Tyramide. Tissues or cells were treated with DAPI for 15 min and mounted with coverslips with using a permanent mounting medium. Photos were acquired by using a Nikon fluorescence microscope (Nikon, Japan).

### CCK-8 assay and propidium iodide (PI) staining assay

CCK-8 (Beyotime, China) was applied to assess cell viability. Cells were seeded in 96-well plates and cultured overnight. After incubation for 0, 2, 4, 6, 12, and 24 h, 100 µl mixture of CCK-8 and serum-free medium at a volume ratio of 1:10 was added and incubated for additional 1 h at 37 °C. The absorbance value (OD) was measured at 450 nm.

For the detection of necrotic cells in the colon, cells were incubated with a mixed solution of Hoechst and PI (#P0137, Beyotime) for 25 min at room temperature and photographed under a fluorescence microscope.

### Subcellular localization analysis

The fluorescence in situ hybridization (FISH) assay and subcellular fractionation assay were performed to detect the subcellular localization of HIF-1α. For FISH assay, 18S rRNA probe was used as positive control. HIF-1α and 18S rRNA were captured with Cy3-labeled probe. Cells were fixed by fixative and dehydrated with gradient ethanol. After prehybridization, HIF-1α and 18S rRNA probes were hybridized in prepared hybridization buffer in NCM460 cells. Cell slides were washed with 2×SSC and 0.1% Triton X-100 at 55 °C for 1 h. Nuclei were marked by staining with DAPI. Confocal microscopy was used to better visualize the presence of HIF-1α and 18S rRNA.

Subcellular fractionation assay was conducted with the employment of PARIS^TM^ kits (Cat. AM1921, Thermo Fisher Scientific, Inc.). Cells were digested and collected in 500 μl fractionation buffer. The mixtures were incubated at 4 °C for 20 min, followed by centrifugation. The supernatants (cytoplasmic fractions) were collected for RNA extraction. The pellets (nuclear fractions) were treated using disruption buffer and used for RNA isolation. The expression of HIF-1α from the cytoplasmic and nuclear fractions was determined by PCR. GAPDH and U6 were separately treated as the cytoplasmic control and the nuclear control.

### Co-immunoprecipitation (Co-IP)

NCM460 cells pretreated as indicated were collected and placed into 1.5 ml tubes (NEST Biotechnology, Wuxi, China) with 500 μl Cell Lysis Buffer (Thermo Fisher Scientific) and 10 μg primary antibody of anti-HIF1α (ab51608, Abcam) or anti-GPX4 (MABF1969, Sigma) at 4 °C overnight. The cell lysis/antibody mixture was poured into a new 1.5 ml tube containing pre-washed Protein A/G Magnetic Beads (Thermo Fisher Scientific) and incubated at room temperature for 1 h. After overnight incubation, the immunocomplexes were washed twice with PBST (pH = 7.4 PBS with 0.1% Triton X-100). Bead-bound proteins were eluted by boiling with 2 × SDS loading buffer before being resolved by SDS-PAGE.

### Dual luciferase reporter assay

The promoter region of GPX4 was amplified by PCR and cloned into pGL3 basic vector (Promega, Madison, USA). HEK 293T cells were transfected with pGL3-GPX4-promoter, pRL-TK, and pcDNA3.1-HIF-1α using Lipofectamine 3000 transfection reagent (Invitrogen). Dual luciferase reporter assay was performed using Dual Luciferase Reporter Assay System (Promega). Renilla luciferase activity was used to normalize transfection efficiency.

### Chromatin immunoprecipitation-qPCR (ChIP-qPCR)

ChIP-qPCR was performed as described previously [22]. NCM460 cells were fixed in 1.5% formaldehyde for 15 min at room temperature and quenched with 125 mM glycine. After cell lysis, the chromatin was fragmented into 100–500 bp by Bioruptor Sonicator (Diagenode), and protein-DNA complexes were immunoprecipitated by 5 μg HIF-1α antibody or 2 μg anti-IgG antibody conjugated with Dynabeads Protein G (Invitrogen) mix on a rotator at 4 °C overnight. After washing, reversal of crosslink and DNA purification, equal amounts of IP (by HIF-1α antibody or IgG control) and input DNA were used as a template for conventional PCR assay using specific primers targeting a region within 100 bp of the putative binding site. The primer sequences for the GPX4 promoter were listed in Table S3.

### Isolation of mouse intestinal epithelial cells (IECs)

Isolation of murine colon epithelial cells was performed as described previously [23]. Briefly, C57BL/6 mouse intestines were opened longitudinally, rinsed with PBS, and cut into 2–3 mm lengths. The epithelial integrity was disrupted by 10 ml digestive solution (300U/ml collagenase XI (Merck), 0.1 mg/ml dispase (Merck), in HBSS) on a shaker at 37 °C for 30 min. Liberated IECs were collected and separated by Percoll gradient centrifugation (Sigma Aldrich). Interface cells were collected and further applied as IECs. Extracted IECs were identified via FACS analysis with antibodies PE-conjugated anti-E-Cadherin and FITC-conjugated anti-EpCAM. Isolated IECs purity and survival rate were both >94%.

### Animal experiments

All procedures involving mice and experimental protocols were approved by the Animal Experiment Committee of Fujian Medical University. C57BL/6 male mice aged 6 to 8 weeks were obtained from the SLAC Laboratory Animal Company (Shanghai, China). All animals were housed in a specific pathogen-free facility and maintained on a 12 h light/12 h dark schedule.

Mice were randomized into groups using a computer-generated randomization sequence (6 mice per group). To induce acute colitis, male C57BL/6 mice were treated with 3% dextran sulfate sodium (DSS; MP Biomedicals) in daily drinking water for 7 days. To stabilize HIF-1α, 8 mg dimethyloxallyl glycine (DMOG; MCE; Cat. HY-15893) was administered intraperitoneally on alternate days. To inhibit the activity of HIF-1α, 2-methoxyestradiol (2-ME2; MCE; Cat. HY-12033) was injected intraperitoneally at 15 mg/kg per mouse. To suppress ferroptosis, ferrostatin-1 (Fer-1; MCE; Cat. HY-100579) was administered at a concentration of 5 mg/kg. To induce ferroptosis and inhibit GPX4, mice were injected with 100 mg/kg RSL3(MCE; Cat. HY-100218A) once daily for 7 consecutive days. The corresponding control mice were injected intraperitoneally with normal saline. For the experimental procedures, mice were observed and recorded every day to assess the disease activity index (DAI) score on a scale of 1–4 as the sum of weight loss, stool consistency and fecal occult blood index (Hemoccult fecal occult blood test, Beckman Coulter, Fullerton, CA), the detailed criteria were described previously [24]. After intervention, mice were sacrificed for the following histological assessment. Serum concentrations of IL-1β, IL-6, and TNF-α were quantified using murine IL-1β, IL-6, and TNF-α ELISA kits (MultiSciences, China).

### Statistical analysis

All experimental data were expressed as mean ± SEM of triplicate measurements in a representative experiment. GraphPad Prism 8.0 software (San Diego, CA, United States) was applied for data analysis. Student’s *t*-test was used to compare two groups, and multiple groups were analyzed by one-way ANOVA with Bonferroni test. *P* < 0.05 was considered as statistically significant.

## Results

### Ferroptosis was induced in UC

**Fig. 1:**
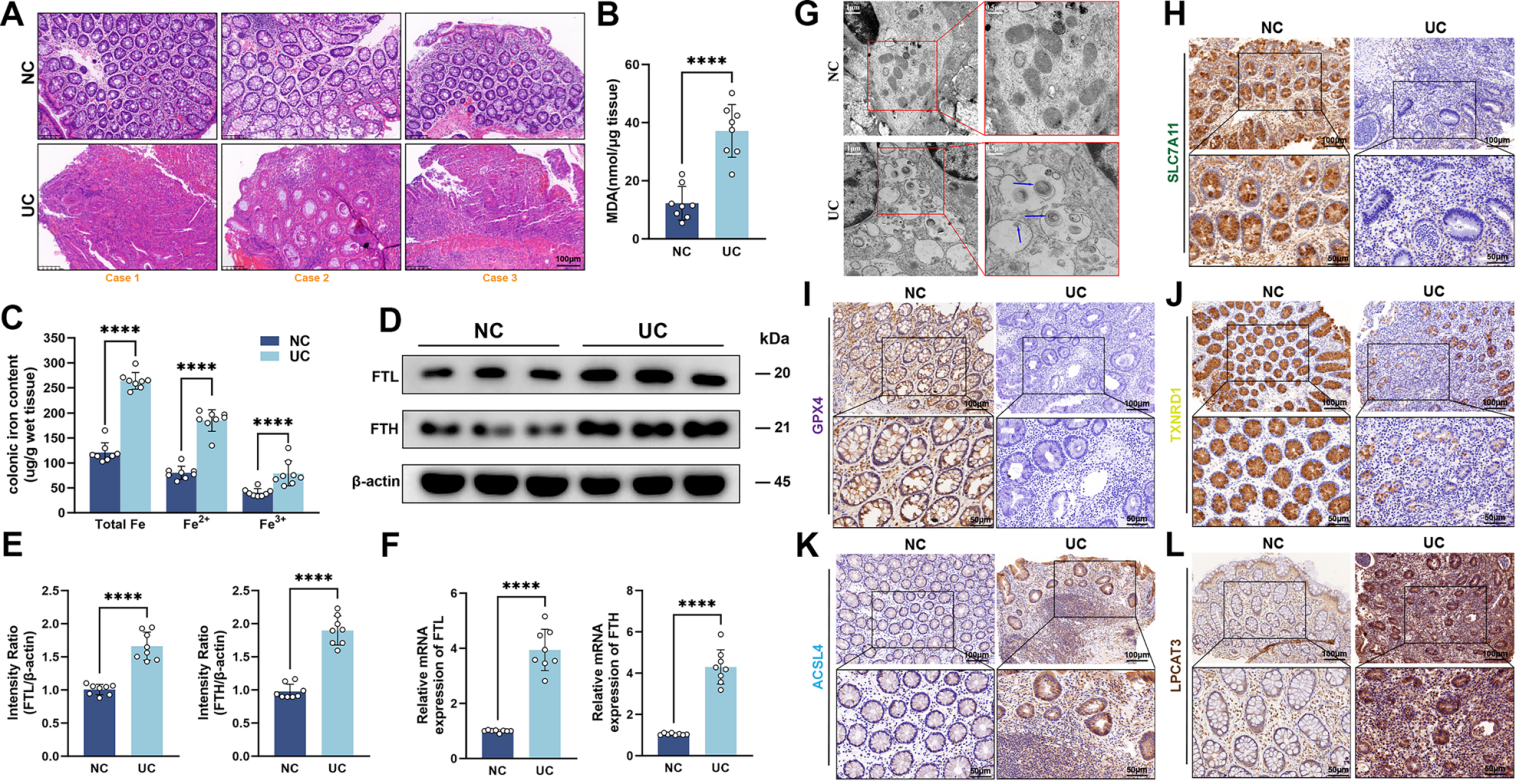
Ferroptosis was induced in ulcerative colitis. **A** Representative H&E staining images of colon tissues from normal controls (NC) and UC patients (Scale: 100 μm). **B** Detection of MDA levels in human intestinal tissues using MDA assay kit. **C** Iron levels of colonic biopsy tissue were determined by iron assay kit. **D**–**F** The protein and mRNA expression levels of FTH and FTL in colon tissues assessed by Western blot and RT-qPCR. **G** Transmission electron micrographs of colon epithelial cells from UC and NC samples (Scale: 1 μm, 0.5 μm). Blue arrows indicate mitochondrial atrophy. **H**–**L** Immunohistochemical staining of SLC7A11, GPX4, TXNRD1, ACSL4, LPCAT3 in UC and NC samples (Scale: 100 μm, 50 μm). ****P < 0.0001.

Accumulating evidence has revealed that ferroptosis participated in the pathogenesis of UC. To assess the propensity for the occurrence of ferroptosis, 8 pairs of UC and healthy control intestinal specimens were collected. Histology was conducted on the biopsy sample. The result indicated that the histological phenotype of retrieved samples primarily manifested as inflammatory cell infiltration, mucosal ulceration, and crypt destruction, which was histologically consistent with UC tissue (Fig. 1A).

Iron accumulation and lipid peroxidation are critical features of ferroptosis [25]. The MDA content is considered as an indicator of lipid peroxidation [26]. Therefore, MDA levels and iron content in the intestine were assessed to verify intestinal ferroptosis. As shown in Fig. 1B, the MDA content in UC tissue was significantly higher than that in healthy controls. In addition, the iron content in UC tissue was increased, especially ferrous iron (Fe^2+^) (Fig. 1C). Ferritin is composed of ferritin heavy chain (FTH) and ferritin light chain (FTL). Ferritin transcription and translation are upregulated when there is an increase in intracellular unstable iron levels [26–28]. Similarly, we found the consistent results in UC colon tissues (Fig. 1D–F). TEM revealed atrophy of the mitochondria in the UC samples (Figs. 1G and S1A), which is consistent with the characteristics of ferroptosis. Moreover, ferroptosis-associated genes were multi-validated by immunohistochemistry. We observed that ferroptosis-related genes exhibited a dysregulated level. *ACSL4* and *LPCAT3*, the positive regulators of ferroptosis, were significantly upregulated in translation, while *GPX4, SLC7A11*, and *TXNRD1*, as negative regulators of ferroptosis, were downregulated (Figs. 1H–L and S1B). These ferroptosis-associated genes also had significant diagnostic effects in other UC RNA-seq datasets (Fig. S1C). The 4-hydroxy-2-nonenal (4-HNE), as one of the indicators of lipid peroxidation [29], was also observed to be elevated along with FTH in UC epithelial cells, suggesting that ferroptosis might occur in UC epithelial cells (Fig. S1D). The above observations indicated that ferroptosis might be activated in UC tissues.

### HIF-1α was involved in ferroptosis of UC

**Fig. 2:**
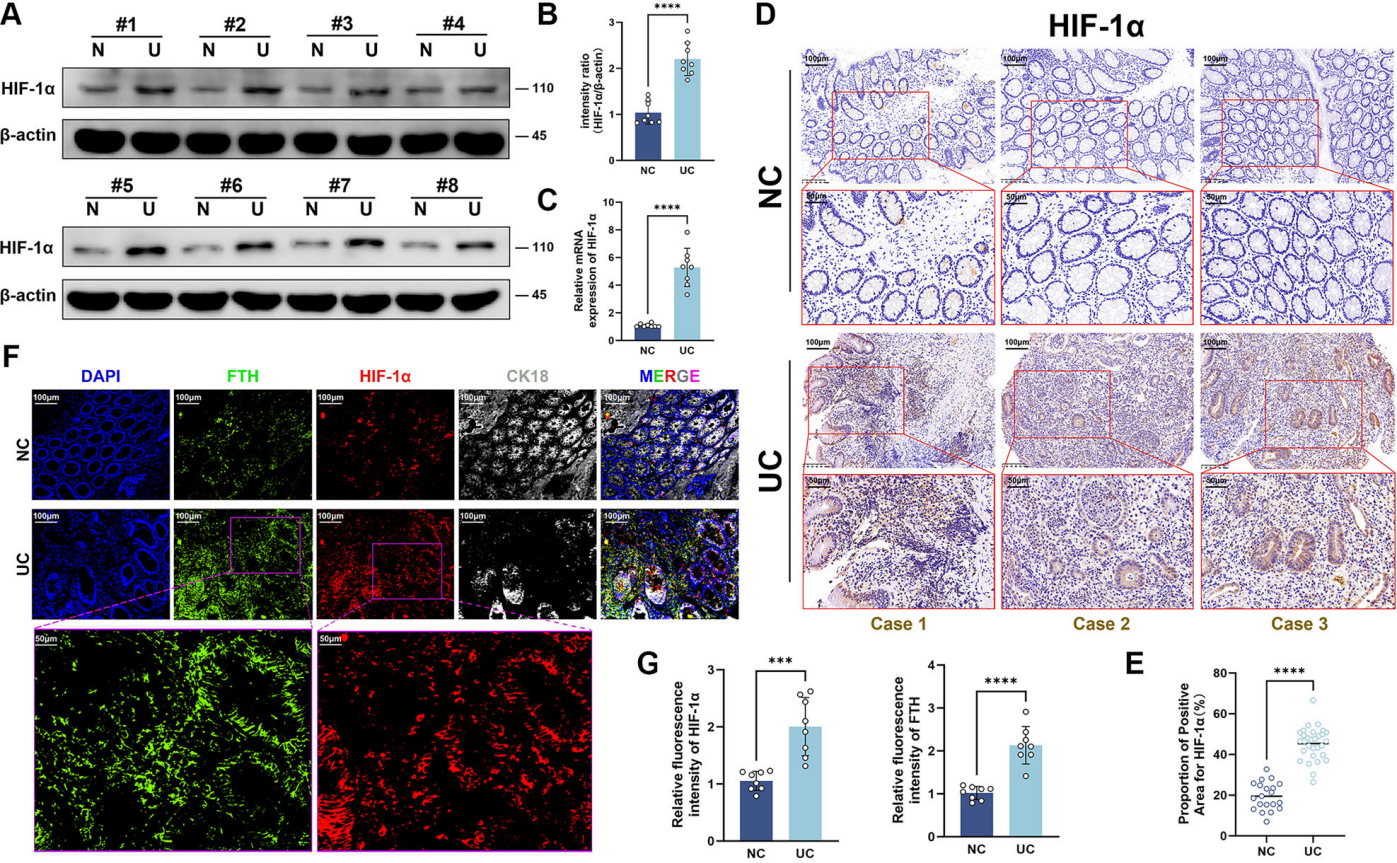
HIF-1α was elevated in UC tissues. **A**–**C** The protein and mRNA expression levels of HIF-1α in colon tissues assessed by Western blot and RT-qPCR, N: normal control, U: ulcerative colitis. **D** Immunohistochemical staining of HIF-1α in UC and normal control (NC) samples (Scale: 100 μm, 50 μm). **E** Percentage of positive area in immunohistochemistry. **F**, **G** Immunofluorescence multilabel staining of HIF-1α, FTH, and cytokeratin 18 (CK18) on colon sections from control and UC patients. Nuclei were stained with DAPI in blue, HIF-1α localization was indicated in red, FTH localization was indicated in green, CK18 staining was indicated in gray (Scale: 100 μm, 50 μm). ***P < 0.001, ****P < 0.0001.

We applied bioinformatics analysis to screen and identify UC-related ferroptosis target *HIF1A*, namely HIF-1α (Fig. S2A). To verify the biological process of HIF-1α in UC tissues, we initially identified the mRNA and protein expressions of HIF-1α in 8 pairs of UC tissues and healthy control tissues. The results revealed that HIF-1α was increased in active UC tissues compared to control groups (Fig. 2A–C). Then we further detected HIF-1α expression and localization in 30 UC tissues and 20 healthy colon tissues with IHC assay. Representative IHC images showed that HIF-1α was mainly distributed in the epithelial cells of active UC (Fig. 2D). Expression of HIF-1α was identified to be significantly elevated (Fig. 2E). Immunofluorescence analysis showed that the increase in HIF-1α and FTH was mainly apparent in epithelial cells (Fig. 2F, G), indicating that HIF-1α might mediate ferroptosis mainly in epithelial cells. In addition, our analysis of mRNA datasets revealed a positive or negative correlation between *HIF1A* and these ferroptosis-associated genes (Fig. S2B). UC single-cell sequencing data also showed that HIF1A and ferroptosis-related genes had more homogenous abundance distributions in epithelial cells than in immune cells (Fig. S3A, B). The above results indicated that HIF-1α might play a role in UC by regulating ferroptosis in epithelial cells.

### HIF-1α ameliorated ferroptosis in inflamed colonic epithelial cells

**Fig. 3:**
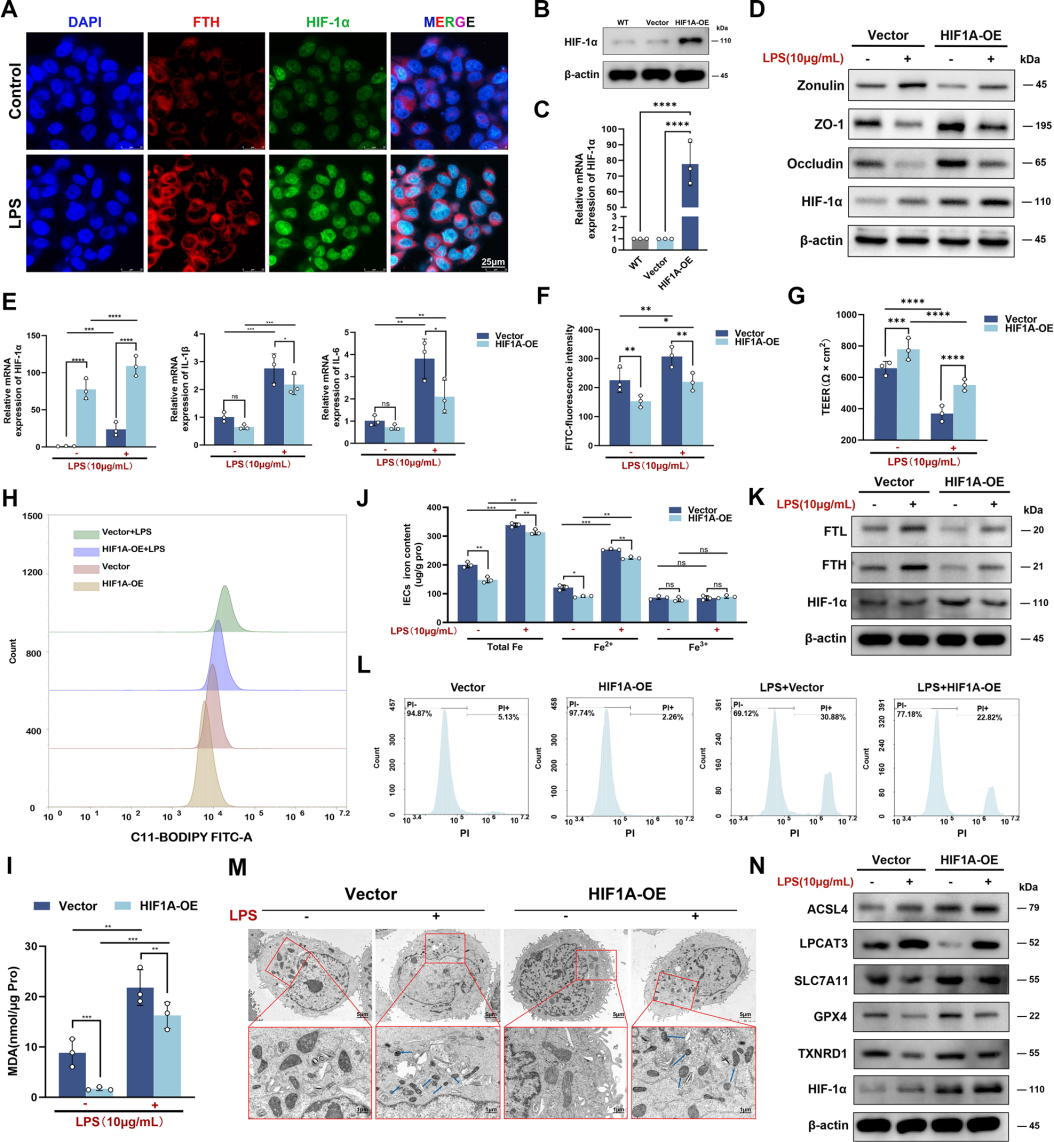
Overexpression of HIF-1α improves ferroptosis in inflammatory colon epithelial cells. **A** Immunofluorescence detection of HIF-1α and FTH in NCM460 cells. Nuclei were stained with DAPI in blue, HIF-1α localization was indicated in green, and FTH localization was indicated in red (Scale: 25 μm). **B**, **C** Western blot and RT-qPCR analysis of HIF-1α in wild type (WT), empty control (Vector), and overexpression group (HIF1A-OE), with β-actin as internal reference. **D** Western blot detection of tight junction proteins (Occludin and ZO-1), zonulin, and HIF-1α expression in intestinal epithelial cells (IECs) of different groups. **E** The mRNA levels of HIF-1α, IL-6, and IL-1β in cells from each group with and without LPS intervention. **F**, **G** Evaluation of intestinal epithelial cell integrity by FITC-dextran permeability assay and transepithelial electrical resistance (TEER). **H** Flow cytometry detection of C11-BODIPY581/591 levels in IECs with or without LPS intervention. **I** MDA levels in human IECs using MDA kit. **J** Iron levels of human IECs in each group were determined by iron assay kit. **K** Western blot detection of iron storage proteins (FTL and FTH) and HIF-1α expression in IECs. **L** Flow cytometric detection of PI-positive cells to analysis necrotic cell death in IECs. **M** Transmission electron microscopy of human IECs under different intervention conditions (Scale: 1 μm, 5 μm). **N** The protein expression levels of ACSL4, LPCAT3, SLC7A11, GPX4, TXNRD1, and HIF-1α in human IECs under different treatments as assessed by Western blot. ns *P* > 0.05, *P < 0.05, **P < 0.01, ***P < 0.001, ****P < 0.0001.

To explore the potential association between HIF-1α and ferroptosis in ulcerative colitis, we first subjected NCM460 cells to LPS stimulation to establish inflammatory colonic epithelial models. Concomitantly, immunofluorescence analysis was performed using specific antibodies against HIF-1α and FTH. The results revealed significant co-localization of HIF-1α and FTH within the cells (Fig. 3A). The results indicated a potential spatial-temporal association between HIF-1α activation and ferroptosis response in inflammatory colonic epithelial cells. To further validate the correlation in vitr*o*, we established stable colonic epithelial cell lines with HIF-1α overexpression or knockdown (Figs. 3B, C and S4A, B). The results showed that the inflammatory cytokines IL-1β and IL-6 in HIF-1α overexpressing colon epithelial cells were lower than those in the control group, and the levels of tight junction proteins ZO-1 and Occludin were higher than those in the control group, while zonulin was lower than that in the control group (Fig. 3D, E). The opposite results were observed in the HIF-1α knockdown group (Fig. S4C). We also investigated the intestinal injury utilizing FITC-dextran and TEER. The results indicated that, in contrast to the untransfected cells within the monolayer, a discernible reduction in intestinal epithelial leakage was observed in the cells subjected to the treatment of HIF-1α overexpression (Fig. 3F, G). Lipid peroxidation levels were assessed by MDA assay and C11-BODIPY581/591 flow cytometry. Under LPS intervention, the lipid peroxidation level of human colon epithelial cells was significantly increased, while HIF-1α overexpression downregulated the lipid peroxidation level (Fig. 3H, I). Iron content assay presented significantly higher iron content (predominantly Fe^2+^) in human colon epithelial cells with LPS induction, and lower iron content in HIF-1α overexpressing cells compared to the control group (Fig. 3J). Western blot analysis suggested that LPS-induced iron storage protein (FTL and FTH) expressions were upregulated, and that HIF-1α overexpressing colonic epithelial cells had lower expression of iron storage proteins (Fig. 3K). PI flow cytometry showed that the proportion of necrotic cells in LPS-induced inflammatory colon epithelial cells was increased, whereas HIF-1α overexpression notably decreased the proportion of necrotic cells (Fig. 3L). TEM showed that LPS-induced inflammatory colon epithelial cells exhibited microscopic morphological characteristics of ferroptosis, such as mitochondrial condensation and mitochondrial cristae reduction, while HIF-1α overexpression impaired the mitochondrial damage in the colonic epithelial cells (Fig. 3M). In contrast, the opposite phenotype of lipid peroxidation, iron imbalance, and mitochondrial structural damage was observed after the silencing of HIF-1α (Fig. S4D–I). Western blot analysis showed that in inflammatory colonic epithelial cells, the expression of ferroptosis-driving molecules (ACSL4 and LPCAT3) was upregulated, while the expression of ferroptosis-protective molecules (GPX4, SLC7A11, and TXNRD1) was downregulated. Moreover, the expressions of GPX4, SLC7A11, TXNRD1, and ACSL4 were increased, but LPCAT3 was decreased after HIF-1α overexpression (Fig. 3N). Contralaterally, the genetic alterations of ferroptosis essential molecules displayed an inverse trend after HIF-1α silence (Fig. S4I). The above results all confirmed that ferroptosis occurred in inflamed colonic epithelial cells, and that HIF-1α suppressed ferroptosis and inflammation in human colonic epithelial cells.

### HIF-1α improved ferroptosis by regulating the transcription of GPX4 in inflammatory colonic epithelial cells

**Fig. 4:**
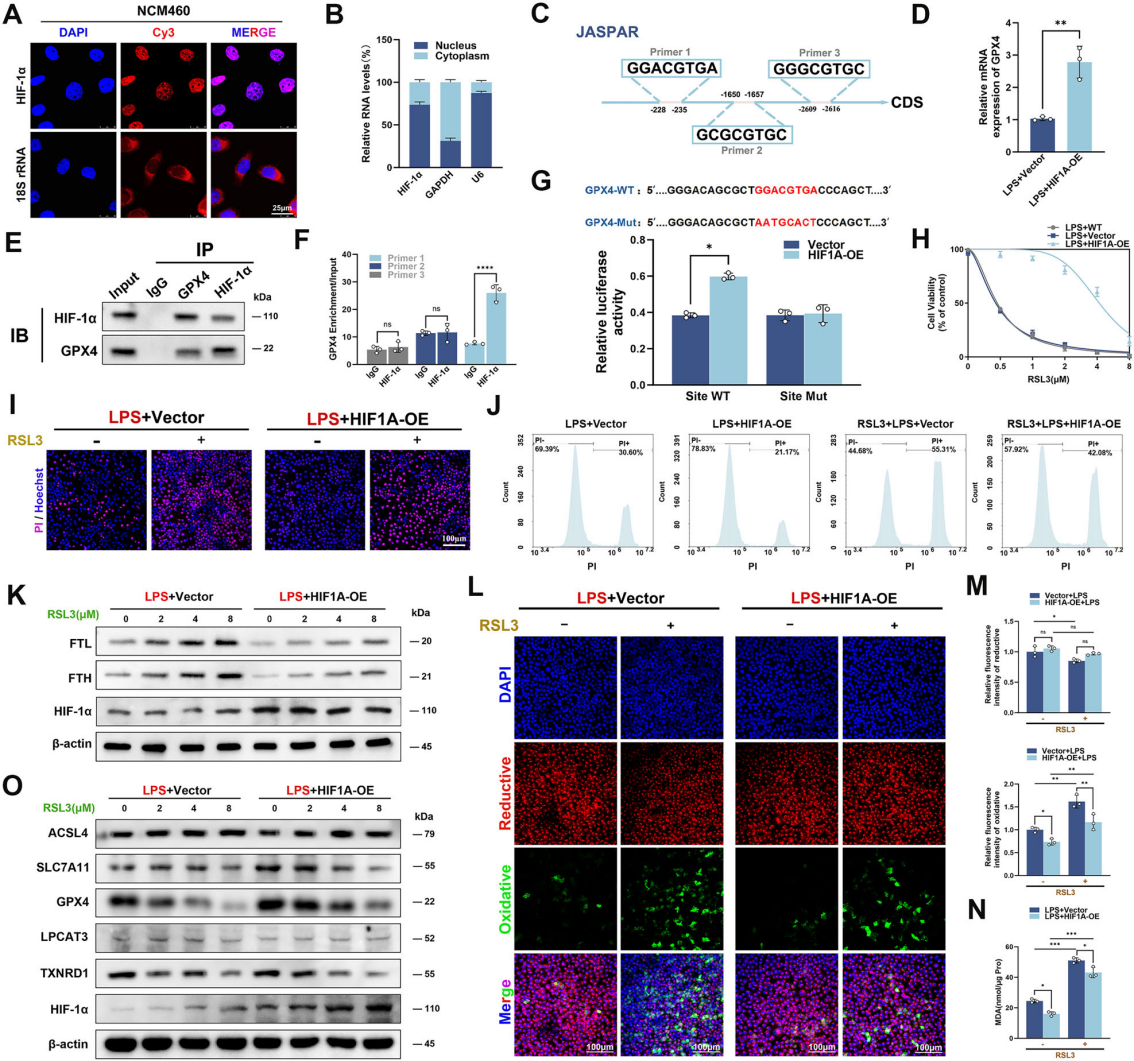
HIF-1α alleviated ferroptosis via targeting GPX4 transcription in inflammatory intestinal epithelial cells (IECs). **A**, **B** Fluorescence in situ hybridization (FISH) and subcellular fractionation assays showing the main localization of HIF-1α within NCM460 cell nucleus. 18S rRNA, GAPDH, and U6 served as cytoplasmic and nuclear controls, respectively (Scale: 25 μm). **C** JASPAR database predicted HIF-1α and GPX4 promoter binding site schematic. **D** RT-qPCR detected the mRNA level of GPX4 in inflammatory IECs with HIF-1α overexpression. **E** Co-IP indicated that HIF-1α might bind to GPX4 to form the HIF-1α/GPX4 complex in inflammatory IECs. **F** ChIP-qPCR confirmed HIF-1α binding to GPX4 promoter. **G** Dual-luciferase reporter assay was performed after transfection with GPX4 promoter wild-type (WT) or mutant luciferase reporter plasmids in HEK 293 T cells. **H** CCK-8 cell assay to assess cell survival under different conditions. **I** Fluorescent detection of cell death by PI staining of RSL3 (4 μM) for 24 h on colon epithelial cells. **J** Flow cytometric detection of PI-positive cells to analyze necrotic cell death after RSL3 and LPS intervention. **K** Western blot analysis of FTL, FTH, and HIF-1α with β-actin as a control. **L**, **M** Fluorescent histogram of lipid peroxidation level in colon epithelial cells detected by C11 BODIPY581/591 fluorescent probe (Scale: 100 μm). **N** MDA levels in human colon epithelial cells with and without RSL3 (4 μM) intervention. **O** Protein expression levels of ferroptosis essential genes (*ACSL4, SLC7A11, GPX4, LPCAT3*, and *TXNRD1*) and HIF-1α detected by Western blot in IECs at different RSL3 concentrations. ns *P* > 0.05, *P < 0.05, **P < 0.01, ***P < 0.001, ****P < 0.0001.

As HIF-1α is a transcription factor that regulates many important biological functions, we initially performed FISH and subcellular fractionation assays to examine the subcellular distribution of HIF-1α. The results showed that HIF-1α was mainly located in the nucleus (Fig. 4A, B). We predicted its ferroptosis target genes through the JASPAR database (https://jaspar.elixir.no/). Interestingly, it was found that only the promoter region of GPX4 had multiple binding sites for HIF-1α (Fig. 4C). Overexpression of HIF-1α in inflamed human colonic epithelial cells resulted in increased GPX4 protein and mRNA levels (Figs. 3N and 4D). Co-IP showed that HIF-1α could directly interact with GPX4 in inflamed human colonic epithelial cells (Fig. 4E). ChIP-qPCR was further performed using GPX4 specific primers corresponding to different promoter regions, and the result illustrated that HIF-1α regulated transcription by binding to the GPX4 promoter (Fig. 4F). Subsequently, the sequence of GPX4 promoter with a putative HIF-1α binding site or a mutated binding site was cloned into pGL3 vector for luciferase reporter assay. The result revealed that HIF-1α specifically targeted the promoter of GPX4 to positively regulate the luciferase activity (Fig. 4G). To further clarify whether HIF-1α improves ferroptosis in human colonic epithelial cells by regulating GPX4, we additionally added the GPX4 inhibitor RSL3 to inflammatory colonic epithelial cells. CCK-8 assay showed that after RSL3 exposure for 72 h, the cell viability of colonic epithelial cells showed dose-dependent reduction with increasing RSL3 concentration, and HIF-1α might protect the cells against RSL3-induced cell death. Considering the cytotoxicity, we chose 4 μM RSL3 as an optimum concentration for ensuing experiments (Fig. 4H). PI fluorescence staining and flow cytometry were applied to evaluate the proportion of necrotic cells, and the results both manifested the increment in percentage of necrotic cells after RSL3 intervention, while overexpression of HIF-1α suppressed acute cell death, indicating that HIF-1α partially protected against epithelial cell death with RSL3 intervention (Figs. 4I, J and S4J). The protein and mRNA expression levels of FTL and FTH in inflammatory intestinal epithelial cells were also statistically increased with RSL3 intervention, but notably decreased in HIF-1α high expression group compared with control group at the same concentration of RSL3 (Figs. 4K and S4K). MDA and C11-BODIPY581/591 fluorescent probes were used to assess lipid peroxidation levels in inflammatory colonic epithelial cells, and the results indicated that the lipid peroxidation level in HIF-1α overexpression group was lower than control group under RSL3 intervention (Fig. 4L–N). Western blot and RT-qPCR demonstrated that among the ferroptosis-related molecules, GPX4 was the only one whose protein and mRNA levels decreased with increasing RSL3 concentration, and the decrease in the HIF1A-OE group was lower than that in the vector group, indicating that overexpression of HIF-1α increased the expression of GPX4 (Figs. 4O and S4L). Overall, HIF-1α could bind to the promoter of GPX4 and stimulate its transcription to reduce ferroptosis in inflammatory colonic epithelial cells.

### HIF-1α ameliorated DSS-induced acute colitis in mice

**Fig. 5:**
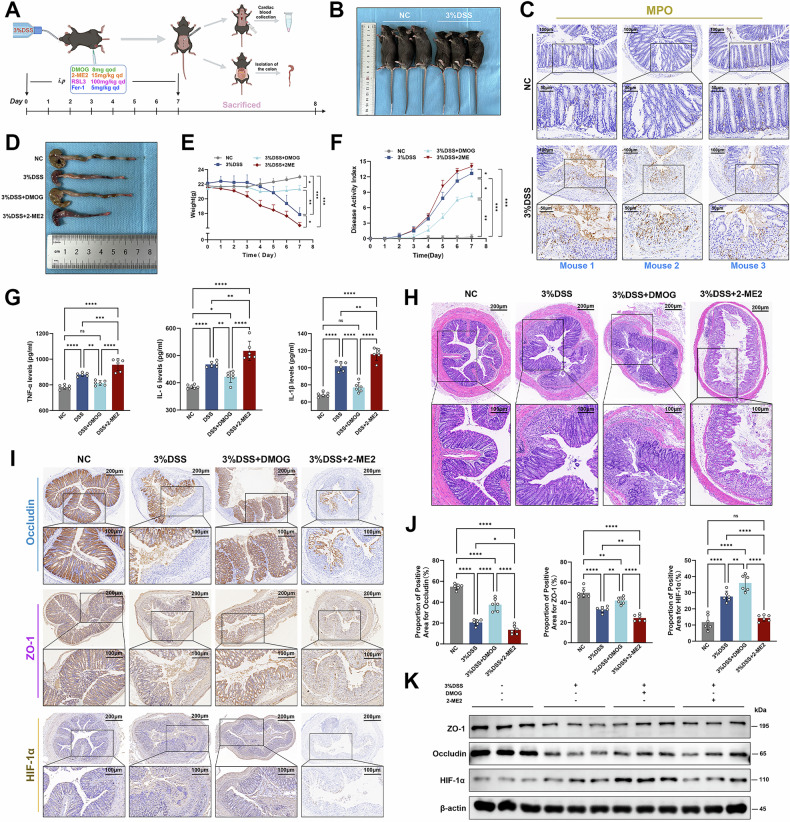
HIF-1α alleviated DSS-induced acute colitis in mice. **A** Intervention methods and cycles of DMOG, 2-ME2, RSL3, and Fer-1 in C57BL/6 male mice. **B** Appearance characteristics of mice after normal control (NC) group and 3% DSS intervention. Red arrow indicates blood residue after defecation. **C** Immunohistochemical staining of MPO in colonic sections (Scale: 100 μm, 50 μm). **D**, **E** Changes of murine colon length and weight after different intervention. **F** Disease activity index (DAI) scores of mice. **G** Expression levels of inflammatory factors IL-1β, IL-6, and TNF-α were detected in mice serum by ELISA kit. **H** H&E staining of colon tissues of mice (Scale: 200 μm, 100 μm). **I**, **J** Immunohistochemical staining of Occludin, ZO-1, and HIF-1α in different groups (Scale: 200 μm, 100 μm). **K** Western blot analysis of ZO-1, Occludin, and HIF-1α in the mice colon tissues. ns *P* > 0.05, *P < 0.05, **P < 0.01, ***P < 0.001, ****P < 0.0001.

To investigate the role of HIF-1α in mice with acute colitis, the mice were intraperitoneally injected with a HIF-1α stabilizer (Dimethyloxallyl Glycine, DMOG) or a HIF-1α inhibitor (2-Methoxyestradiol, 2-ME2) to assess the general conditions and intestinal barrier function (Fig. 5A). The acute colitis mice with 3% DSS administration presented with reduced body size, with pale skin, lusterless hairs, and the presence of hemorrhages in the intestinal tract (Fig. 5B), MPO staining revealed peripheral neutrophil infiltration particularly in the colitis tissues (Fig. 5C). We identified that the shortened intestine in DSS-induced colitis mice was improved with the intervention of DMOG, but was exacerbated after 2-ME2 intervention (Figs. 5D and S5A). Similarly, we also found that DMOG significantly ameliorated weight loss and reduced the disease activity index (DAI) of mice with DSS-induced colitis, while 2-ME2 had the opposite effect (Fig. 5E, F). We also measured the expression levels of serum inflammatory cytokines (IL-1β, IL-6, and TNF-α) in each group of mice. The results suggested that DMOG intervention reduced the secretion of inflammatory cytokines in mice with DSS-induced colitis, whereas the decreased level was significantly reversed by 2-ME2 intervention (Fig. 5G). Further histological analysis showed that DMOG ameliorated the pathological symptoms of colitis, including mucosal erosion and inflammatory infiltration, while 2-ME2 aggravated tissue damage in colitis (Figs. 5H and S5B). ZO-1 and Occludin, as signature tight junction proteins, were involved in the integrity of the intestinal mucosal barrier. Immunohistochemical staining and Western blot showed that the positive areas of ZO-1 and Occludin in the colon tissue of normal control (NC) mice were arrayed compactly and orderly, while DSS and 2-ME2 intervention induced disturbances in alignment of tight junction protein, but DMOG alleviated the disrupted tight junction proteins (Figs. 5I–K and S5C–F). Likewise, we observed the same results at the mRNA level (Fig. S5G). The above results indicated that elevated HIF-1α ameliorated DSS-induced colitis in mice.

### Inhibition of ferroptosis alleviated DSS-induced acute colitis in mice

**Fig. 6:**
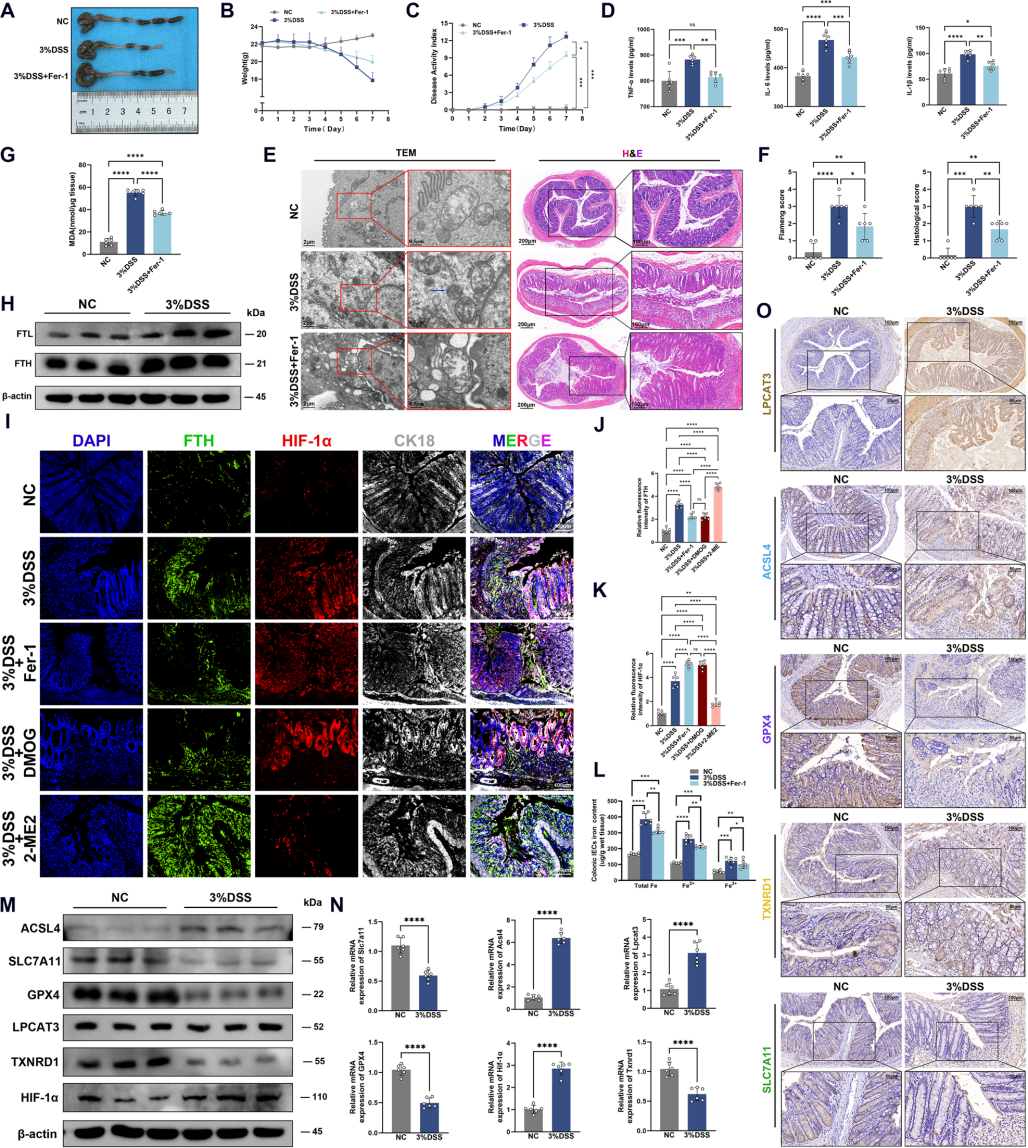
Inhibition of ferroptosis alleviated DSS-induced acute colitis in mice. **A**, **B** Changes of murine colon length and weight after DSS and Fer-1 intervention. **C** Disease activity index (DAI) scores of mice. **D** Expression levels of inflammatory factors IL-1β, IL-6, and TNF-α were detected in mice serum by ELISA kit. **E**, **F** Transmission electron micrographs (TEM) and H&E staining of murine colon tissues in different groups. **G** MDA levels in the colonic tissues of mice in each group using the MDA kit. **H** Western blot analysis of FTL and FTH with β-actin as control. **I–K** Immunofluorescence staining of HIF-1α, FTH, and CK18. Nuclei were stained with DAPI in blue, HIF-1α localization was indicated in red, FTH localization was indicated in green, CK18 staining was indicated in gray (Scale: 100 μm). **L** Determination of iron content of intestinal epithelial cells in mouse colon tissues using iron assay kit. **M**, **N** The protein and mRNA levels of ferroptosis key genes (*ACSL4, SLC7A11, GPX4, LPCAT3*, and *TXNRD1*) and HIF-1α in colon tissues of NC and DSS-induced acute colitis mice. **O** Immunohistochemical staining of LPCAT3, ACSL4, GPX4, TXNRD1, and SLC7A11 in colon tissues of normal control (NC) and DSS-induced acute colitis mice. ns *P*> 0.05, *P < 0.05, **P < 0.01, ***P < 0.001, ****P < 0.0001.

To determine the role of ferroptosis in acute colitis in mice, we additionally added Fer-1, a ferroptosis inhibitor, to 3% DSS-induced acute colitis mice. We observed that the degree of colon shortening and body weight loss in mice with acute colitis were improved under Fer-1 intervention (Figs. 6A, B and S6D), whereas the DAI score and the levels of serum inflammatory cytokines were decreased in Fer-1-intervened mice (Fig. 6C, D). The effect of Fer-1 on the intestinal structure was further observed by H&E staining and TEM. H&E staining confirmed the ameliorative effect of Fer-1 on colitis, including the improvement of intestinal structural damage and the reduction of inflammatory infiltration; TEM observation indicated that Fer-1 improved the mitochondrial damage in the colonic tissue of colitis (Fig. 6E, F). To detect the PI staining in IECs, the primary murine IECs were isolated and identified by flow cytometry (Fig. S6A, B). The PI-positive cells were then further detected by flow cytometry. We found that the number of PI-positive IECs was decreased after Fer-1 treatment (Fig. S6C). The MDA assay showed that the lipid peroxidation level in the colon tissue of colitis mice was elevated, while Fer-1 reversed this effect (Fig. 6G). RT-qPCR and Western blot showed that the expression levels of the iron storage proteins in the colon tissue of colitis mice were significantly increased (Figs. 6H and S6E, F). Immunofluorescence staining showed the consistent trend, and FTH was primarily expressed in the epithelium. In addition, intervention with Fer-1 or DMOG could reverse the elevated FTH, while 2-ME2 increased FTH levels (Fig. 6I–K). The iron assay showed that the iron content in the colonic epithelial cells of colitis mice was significantly increased (especially Fe^2+^), which was reversed by Fer-1 (Fig. 6L). Similar results were observed at the protein and mRNA levels, that is, the expression level of ACSL4 was elevated, but SLC7A11, GPX4, and TXNRD1 were decreased in the colonic tissue of acute colitis mice (Fig. 6M, N). Interestingly, IHC staining results revealed that, except for TXNRD1, the expression levels of ACSL4 and LPCAT3 in colitis mice were higher than those in the normal control group, while SLC7A11 and GPX4 displayed the opposite consequence (Figs. 6O and S6G). The results above suggested that DSS-induced colitis mice exhibited ferroptosis, and inhibition of ferroptosis could improve acute colitis.

### The HIF-1α/GPX4 pathway was involved in ferroptosis in DSS-induced colitis mice

**Fig. 7:**
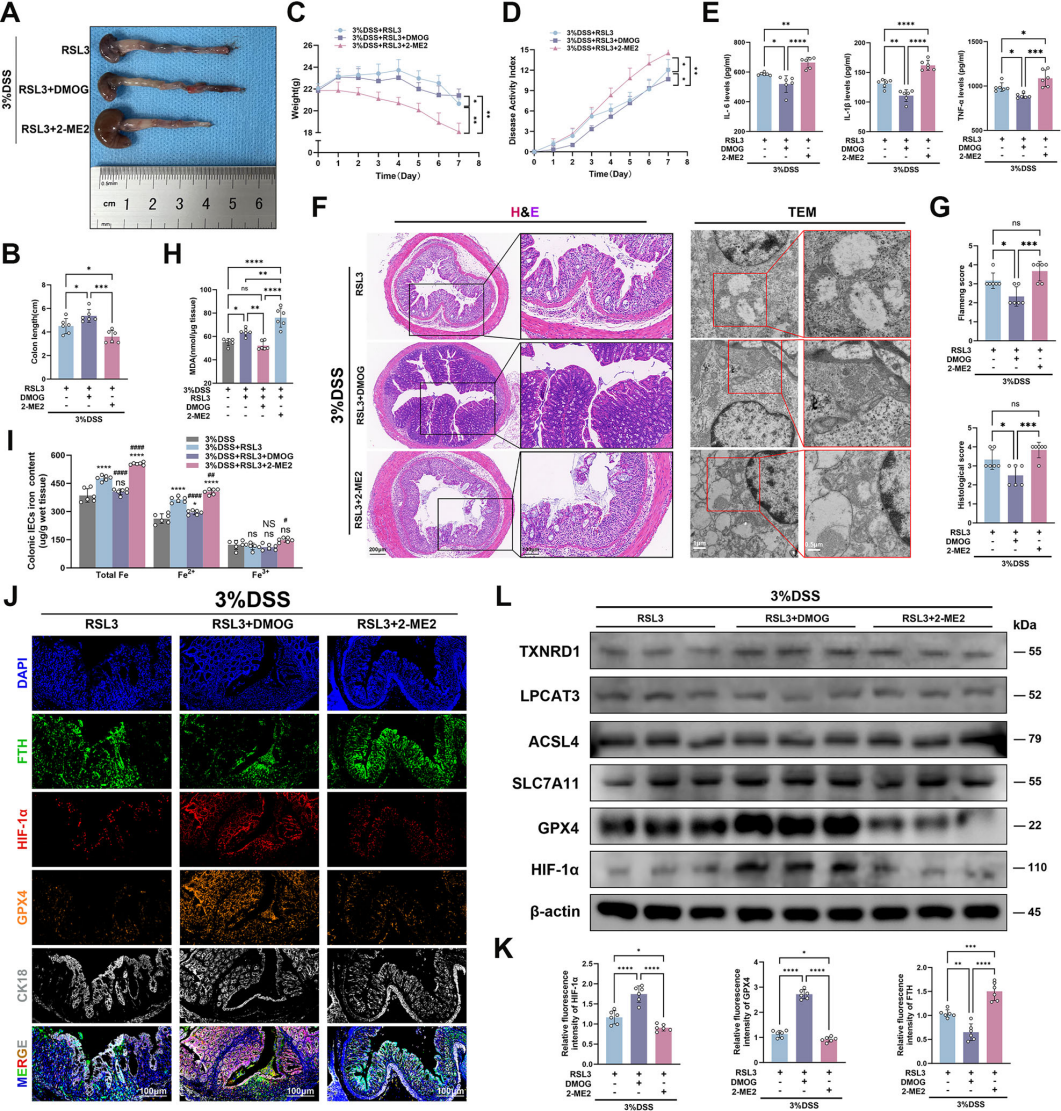
The HIF-1α/GPX4 pathway may ameliorate DSS-induced colitis in mice by suppressing ferroptosis in colonic epithelial cells. **A**–**C** Changes of murine colon length and body weight after RSL3, DMOG, and 2-ME2 intervention. **D** Disease activity index (DAI) scores of mice. **E** Expression levels of inflammatory factors IL-1β, IL-6, and TNF-α were detected in mice serum by ELISA kit. **F**, **G** H&E staining and transmission electron micrographs (TEM) observation of the murine colon tissues after RSL3, DMOG, and 2-ME2 intervention. **H** MDA levels in the colonic tissues of mice using the MDA kit. **I** Determination of iron content of intestinal epithelial cells in mouse colon tissues using iron assay kit. **J**, **K** Multicolor immunofluorescence staining of HIF-1α, GPX4, FTH, and CK18 on mice colonic sections. Nuclei were stained with DAPI in blue, HIF-1α localization was indicated in red, GPX4 localization was indicated in orange, FTH localization was indicated in green, and CK18 staining was indicated in gray. **L** Protein expression levels of ferroptosis key genes (*TXNRD1, LPCAT3, ACSL4, SLC7A11, GPX4*) and HIF-1α in colon tissues of mice with acute colitis under the intervention of RSL3. ns *P* > 0.05, *P < 0.05, **P < 0.01, ***P < 0.001, ****P < 0.0001; NS *P* ＞ 0.05, #*P* < 0.05, ##*P* < 0.01, ####*P* < 0.0001 vs. 3%DSS + RSL3 group.

We have confirmed in vitro that HIF-1α may bind to GPX4 to form a positive feedback loop that affects ferroptosis in inflammatory colonic epithelial cells. Therefore, to further confirm this result in vivo, we regulated the level of HIF-1α while simultaneously administering the GPX4 inhibitor RSL3 to DSS-induced acute colitis mice to observe changes in the mice’s general condition, intestinal barrier function, and ferroptosis. Acute colitis mice treated with RSL3 alone showed significant colon shortening, weight loss, an increased DAI score, and inflammatory cytokines; these conditions were reversed with RSL3 + DMOG combination treatment, while the RSL3 + 2-ME2 group experienced further deterioration (Fig. 7A–E). H&E staining confirmed the ameliorative effect of DMOG on colitis under RSL3 intervention, including the amelioration of intestinal structural damage and the reduction of inflammatory infiltration; TEM revealed that DMOG ameliorated mitochondrial damage in colonic tissue from colitis, while 2-ME2 aggravated all these situations (Fig. 7F, G). Flow cytometry showed that the number of PI-positive IECs was significantly increased in mice with colitis intervened with RSL3, while the number of PI-positive IECs was decreased after DMOG intervention, with 2-ME2 having the opposite effect (Fig. S7A, B). The MDA assay showed that the RSL3 intervention further increased lipid peroxidation in the colonic tissue of mice with colitis, while DMOG attenuated this effect and 2-ME2 aggravated it (Fig. 7H). The iron assay kit showed that the iron content (especially Fe^2+^) in the colon epithelial cells of mice with colitis under RSL3 intervention was significantly increased, and DMOG could reverse this situation (Fig. 7I). The results of immunofluorescence and Western blot showed that in the colon tissue of mice with acute colitis intervened with RSL3, the expression of GPX4 was positively correlated with HIF-1α, while the expression of the other ferroptosis genes did not change significantly (Fig. 7J–L). The same results were observed at the mRNA levels (Fig. S7C). In addition, the expression level of the iron storage protein FTH was negatively correlated with HIF-1α (Fig. 7J, K). Collectively, it is suggested that enhancing the expression of HIF-1α can upregulate the level of GPX4 to inhibit ferroptosis in colonic epithelial cells, thereby improving colitis in mice.

## Discussion

UC refers to an inflammatory disease characterized by persistent damage to colonic epithelial cells [30]. To date, the pathogenesis of UC remains unclear. Existing evidence has shown that ferroptosis is associated with the progression of UC [31]. Herein, we demonstrated that ferroptosis was induced in the colon tissue of UC, and HIF-1α might serve as the crucial target of ferroptosis in UC. Especially, we revealed that HIF-1α activated GPX4 transcription in colonic epithelial cells by binding to the GPX4 promoter to inhibit ferroptosis in UC.

HIF-1α belongs to the hypoxia-inducible factor family and has been shown to be a key regulator of barrier integrity during colonic mucosal injury, and deficiency of HIF-1α in IECs would exacerbate DSS-induced colitis [32, 33]. In contrast, other studies argued that activation of HIF-1α in immune cells may lead to colitis in mice [16, 17]. Consistently, our results revealed that the overexpression of HIF-1α could dampen the inflammatory response of IECs and regulate the intestinal cell barrier function, indicating that HIF-1α exhibited as a protective molecule in the epithelial cells during UC.

In the IECs of healthy population, the HIF signaling pathway is activated to maintain cell function due to transient hypoxia caused by changes in oxygen demand and blood flow [34]. Under normoxic conditions, prolyl hydroxylases (PHDs) were utilized, resulting in HIF-1α degradation and maintaining a relatively balanced state. However, in patients with UC, there is a vicious circle of persistent hypoxia due to the death of IECs and damage to the barrier, increased antigen leakage, and inflammation in the intestinal lumen. At this time, HIF-1α is incapable of being effectively degraded, and is pathologically elevated, but fails to exert a protective effect [35–37]. Why the elevation of HIF-1α in the intestinal tissues of UC patients did not play a protective role and alleviate colitis, we speculate that the pathologically elevated level of HIF-1α may not be sufficient to play a protective effect by inducing the expression of relevant protective genes, therefore, intervention with PHDs inhibitor (DMOG) in this study resulted in increasingly stable expression levels of HIF-1α in the intestines of colitis mice, contributing to the protection of intestinal barrier function by inducing the expression of protective genes.

It is widely recognized that the cystine/glutamate antiporter (system Xc⁻, primarily composed of SLC7A11 and SLC3A2), which imports cystine, as well as the reduction of cystine to cysteine via glutathione and/or TXNRD1 dependency, and the GPX4-mediated reduction of phospholipid hydroperoxides (PL-OOH) to the corresponding alcohols (P-LOH), all help protect cells from death induced by oxidative stress conditions, thereby playing crucial roles in protecting against ferroptosis [38–40]. ACSL4 and LPCAT3 are considered important drivers of ferroptosis by inducing lipid peroxidation under various pathophysiological conditions [5, 41]. We found that overexpression of HIF-1α could attenuate lipid peroxidation and iron levels in inflammatory IECs, as well as upregulate the expression levels of protective ferroptosis key genes (*SLC7A11, GPX4*) and downregulate the expression levels of driver ferroptosis key genes (*LPCAT3*). Our results all suggested that HIF-1α was a negative regulator of ferroptosis, which alleviated IECs inflammation by inhibiting ferroptosis. Likewise, Li et al. found that activation of HIF-1α might inhibit ferroptosis induced by erastin and RSL3 in gastric cancer cells [42]. However, in vascular smooth muscle cells, defects in histone acetyltransferase P300 can promote ferroptosis by activating the HIF-1α/HMOX1 axis [43]. The reasons for the discrepancy of HIF-1α biological function in different diseases may be related to cell-specific gene regulation, differences in signaling pathways, and complex cell microenvironments.

**Fig. 8:**
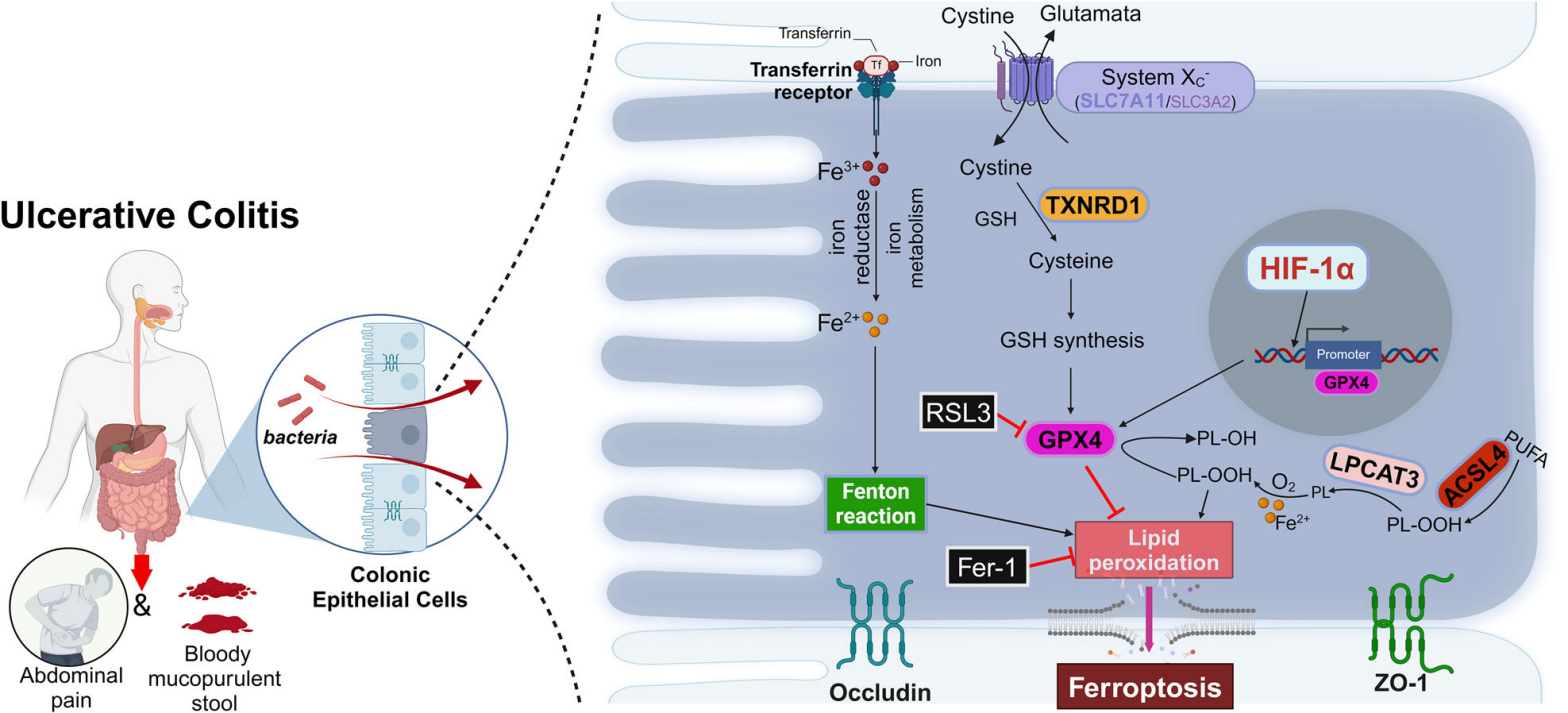
The schematic diagram illustrated the proposed mechanism of HIF-1α in UC. HIF-1α alleviated ferroptosis on intestinal epithelial cells by regulating the GPX4 transcription in UC (Created with BioRender.com).

HIF-1α is a transcription factor that regulates multiple important biological processes [44, 45]. Our research confirmed that HIF-1α mediated GPX4 expression through JASPAR database predictions and ChIP-qPCR validation. GPX4 is a selenoprotein initially discovered by Ursini et al. through biochemical purification, and it is the main enzyme in mammalian cells that catalyzes the reduction of PLOOHs, while reduction of GPX4 leads to the accumulation of PLOOHs, which causes rapid and irreversible damage to the plasma membrane, triggering lipid peroxidation and resulting in ferroptosis in cells [5, 46, 47]. Consequently, GPX4 is a key protective factor against ferroptosis [48]. Research has confirmed that hydroxysafflor yellow A (HSYA) can inhibit myocardial cell ferroptosis by activating HIF-1α, which ultimately increases the expression of SLC7A11 and GPX4, thereby reducing myocardial ischemia/reperfusion injury in mice [49]. Zou et al. found that HIF-1α can activate transcription by binding to the promoter of KCNQ1OT1, and that the μ opioid receptor (MOR) can alleviate ferroptosis in hepatocytes during hepatic ischemia-reperfusion injury through the HIF-1α/KCNQ1OT1 axis [50]. These evidences are concordant with our findings that HIF-1α inhibits ferroptosis via regulating the transcription of GPX4 in IECs (Fig. 8).

There are some deficiencies in the present study. First, the bioinformatics analysis in this study was based on online databases. Although the expressions of HIF-1α and key ferroptosis-related genes were validated in colonic tissues from UC patients, larger sample sizes are needed to for a better interpretation of results. Second, the mouse model of acute colitis was unable to fully mimic the complex pathology of UC, and in vivo models that are more compatible with the characteristics of UC should be developed in the future. Third, while our study focused on the role of HIF-1α in intestinal epithelial cells, we did not examine its effects on immune cells, which play a crucial role in UC pathogenesis. This may limit the comprehensiveness of our mechanistic interpretation. Additionally, the absence of HIF-1α knockout mice may limit the depth and accuracy of certain mechanistic investigations. Nevertheless, our study still provides theoretical support and experimental evidence for elucidating the pathogenesis of UC and developing clinical therapeutic strategies.

## Conclusion

In summary, this study enriched the understanding of the pathogenesis of UC and revealed for the first time that HIF-1α played a crucial target in ferroptosis of UC. HIF-1α may improve UC by regulating the transcription of GPX4 to inhibit ferroptosis in IECs.

## Supplementary information

supplementary material

Western blot originai image

## Data Availability

The datasets used and/or analyzed during the current study are available from the corresponding author upon reasonable request.
