# Supplementary material for: HIF-1α alleviates ferroptosis in ulcerative colitis by regulation of GPX4
Source: Cell Death Dis. 2025 Jul 22;16(1):542. doi: 10.1038/s41419-025-07883-8 (PMC12280037; doi:10.1038/s41419-025-07883-8)
Supplement: Supplementary file 1 — supplementary material [file 41419_2025_7883_MOESM1_ESM.pdf]

Supplementary materials

HIF-1α alleviates ferroptosis in ulcerative colitis by regulation of GPX4

Weitao Hu[†], Yanliang Cai[†], Daxing Cai, Zongchi Chen, Siying Huang, Su Zhang, Huie Zhuang, Taiyong Fang[*], Xiaoqing Chen[*]

Supplementary Figures

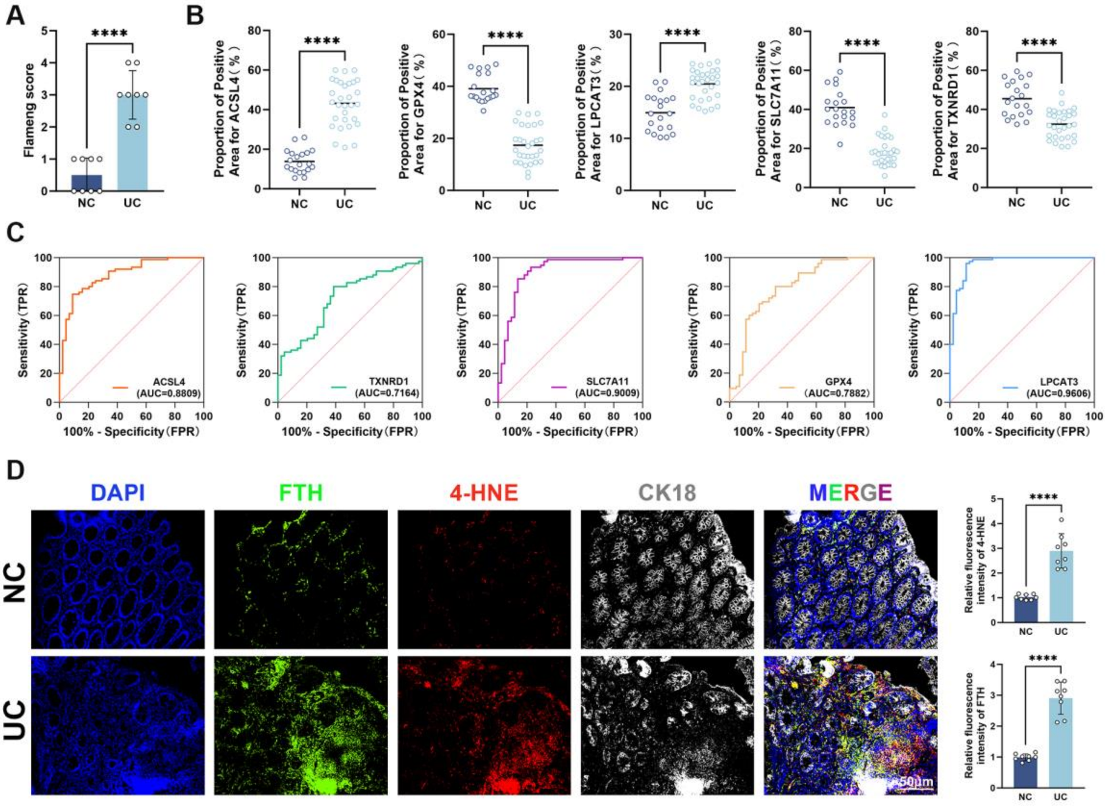

Figure S1. Ferroptosis participated in epithelial death in UC tissues. (A) The mitochondrial Flameng score was evaluated to assess the extent of mitochondrial damage in healthy controls and UC tissues. (B) Immunohistochemistry slides were evaluated to determine the percentage of positive areas for ACSL4, GPX4, LPCAT3, SLC7A11, and TXNRD1 using ImageJ software. (C) The prediction efficiency of ferroptosis genes in UC was determined based on the receiver operating characteristic (ROC) curve of the GSE206171 dataset. Area under the curve (AUC) values of more than 0.7 were considered satisfactory accuracy. (D) Multicolor immunofluorescence staining of FTH, 4-HNE, and CK18 on mice colonic sections. Nuclei were stained with DAPI in blue, FTH localization was indicated in green, 4-HNE localization was indicated in red, and CK18 staining was indicated in grey (Scale: 50μm). ****$P < 0.0001$.

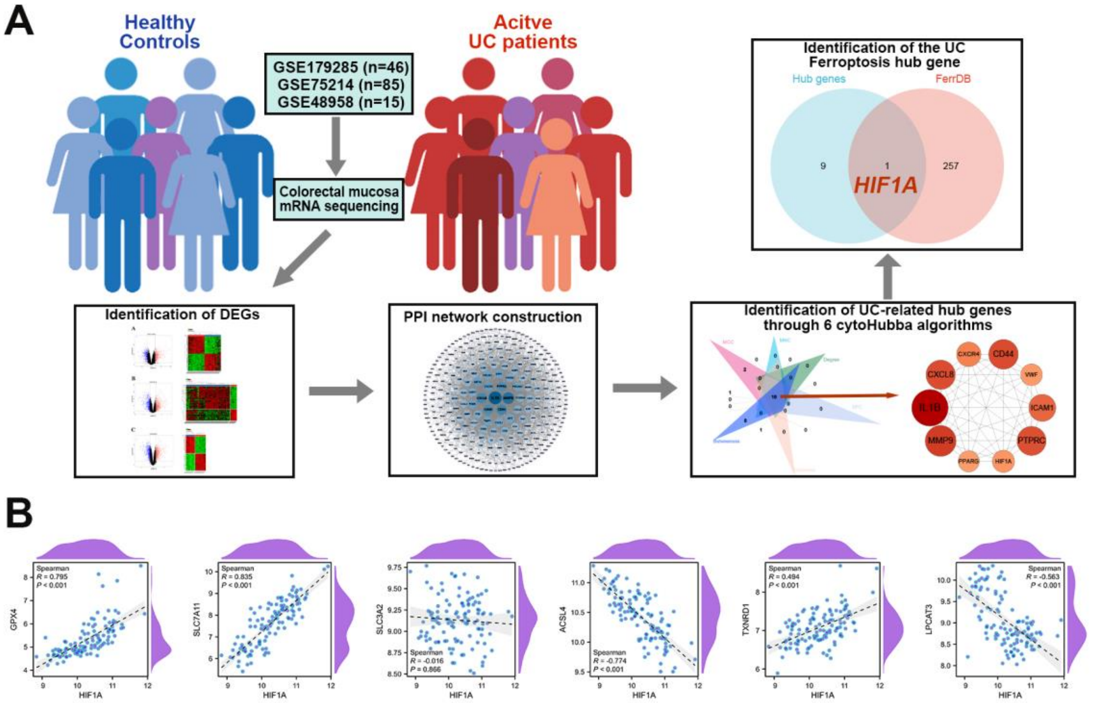

Figure S2. The detailed screening process of HIF1A gene signature and the association between HIF1A and ferroptosis in UC. (A) The intersection of the UC hub genes and ferroptosis hallmark gene sets (258 genes) was performed, and *HIF1A* was extracted as the final ferroptosis candidate gene via bioinformatics analysis. (B) Spearman's correlation analyses of HIF1A with ferroptosis-related genes (*GPX4, SLC7A11, SLC3A2, ACSL4, TXNRD1, LPCAT3*) were performed via GSE206171 dataset.

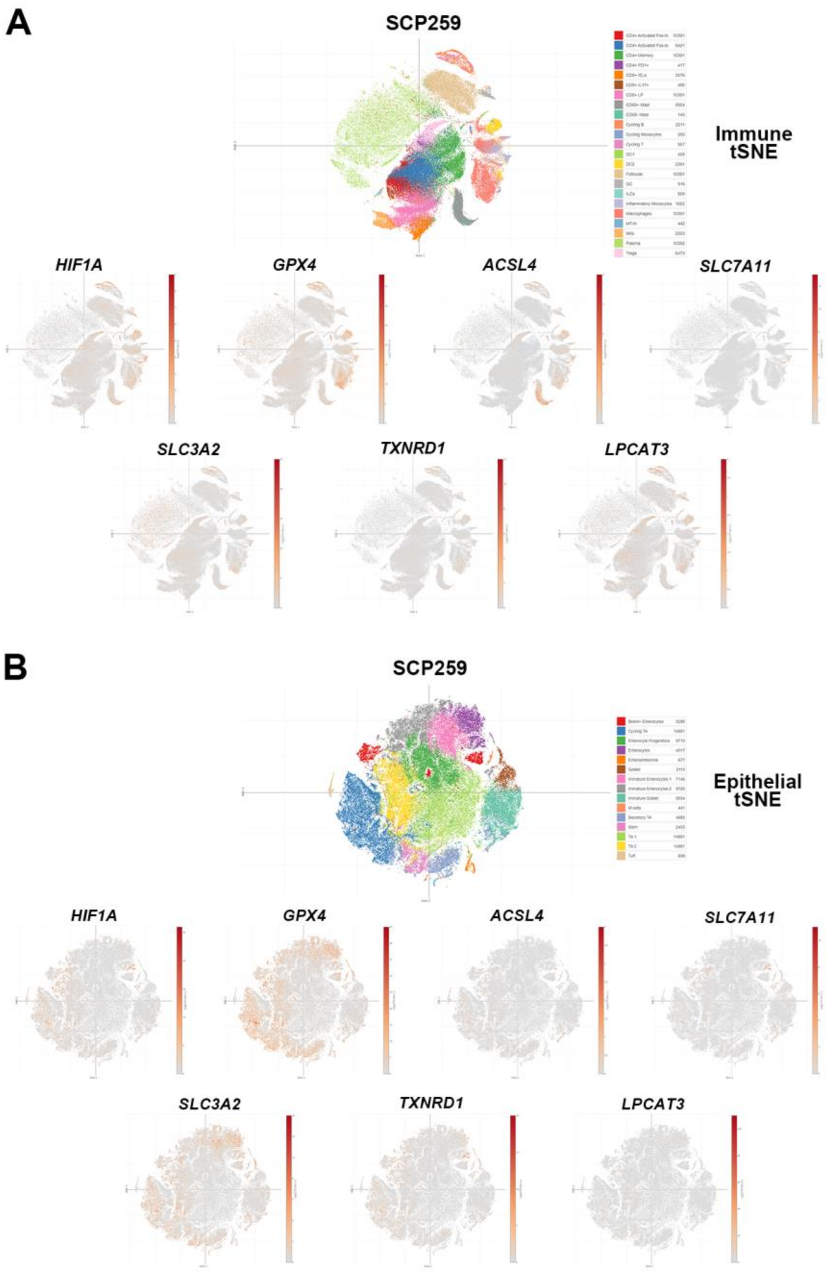

Figure S3. The t-SNE algorithm was performed to evaluate the distribution of HIF1A and ferroptosis related molecules in ulcerative colitis tissues. (A, B) Distribution of HIF1A and ferroptosis related molecules (GPX4, ACSL4, SLC7A11, SLC3A2, TXNRD1, LPCAT3) in immune or epithelial cell clusters based on SCP259 dataset.

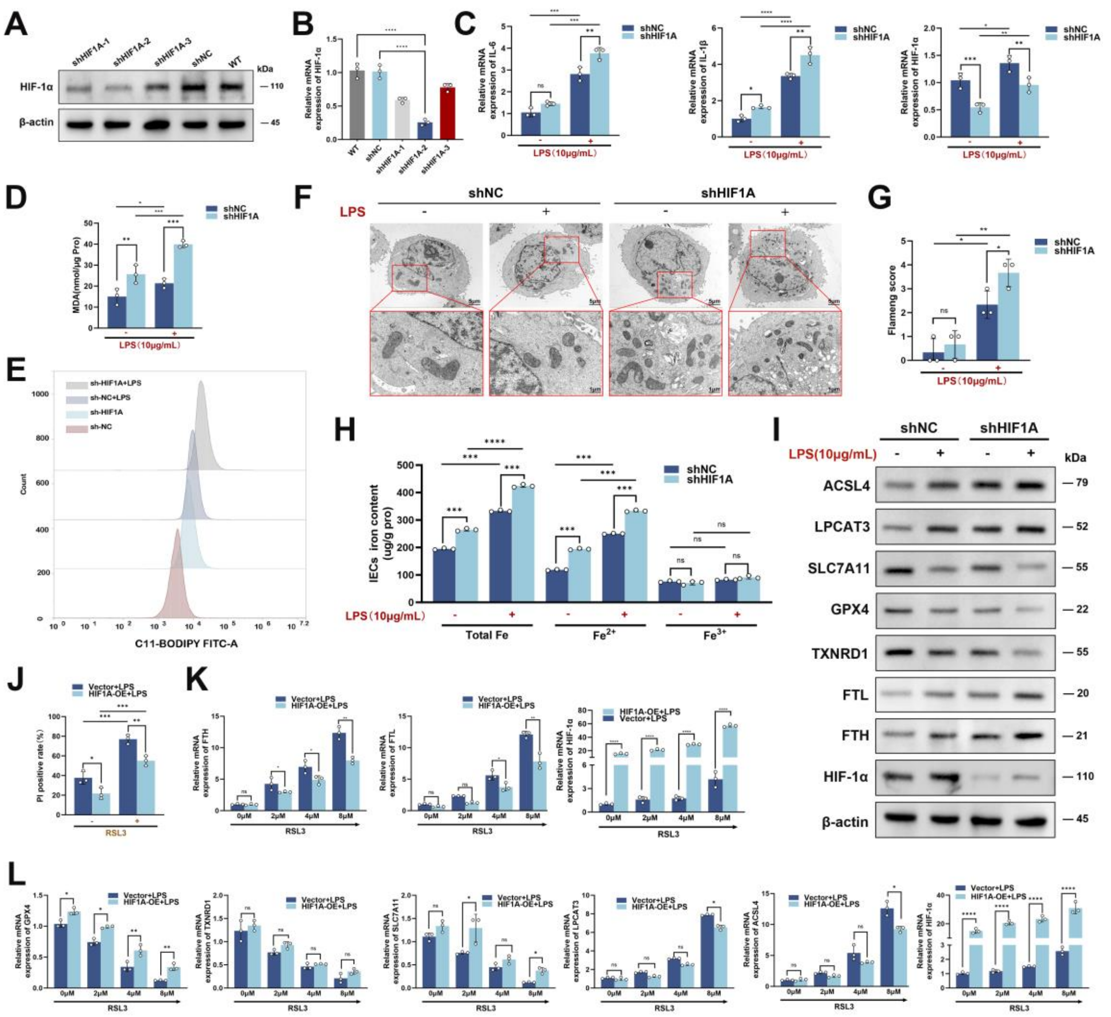

Figure S4. The effect of HIF-1α on ferroptosis in inflammatory colon epithelial cells. (A, B) Western blot and RT-qPCR analysis were performed to detect HIF-1α expression after transfection of HIF-1α shRNA in NCM460 cells. (C) The mRNA levels of HIF-1α, IL-6 and IL-1β in cells from each group with and without LPS intervention. (D) MDA levels in human IECs using MDA kit. (E) Flow cytometry detection of C11-BODIPY levels in IECs with or without LPS intervention. (F-G) Transmission electron microscopy images and mitochondrial damage scores of human IECs under different intervention conditions (Scale: 1μm, 5μm). (H) Iron levels of human IECs in each group were determined by iron assay kit. (I) Western blot detection of iron storage proteins (FTL and FTH), ACSL4, LPCAT3, SLC7A11, GPX4, TXNRD1 and HIF-1α expression in IECs. (J) Quantitative analysis of PI fluorescent staining. (K) The mRNA levels of HIF-1α, FTL and FTH in cells from each group with and without LPS intervention. (L) The mRNA levels of ACSL4, LPCAT3, SLC7A11, GPX4, TXNRD1 and HIF-1α in human IECs under different treatments were assessed by RT-qPCR. ns $P > 0.05$, *$P < 0.05$, **$P < 0.01$, ***$P < 0.001$, ****$P < 0.0001$.

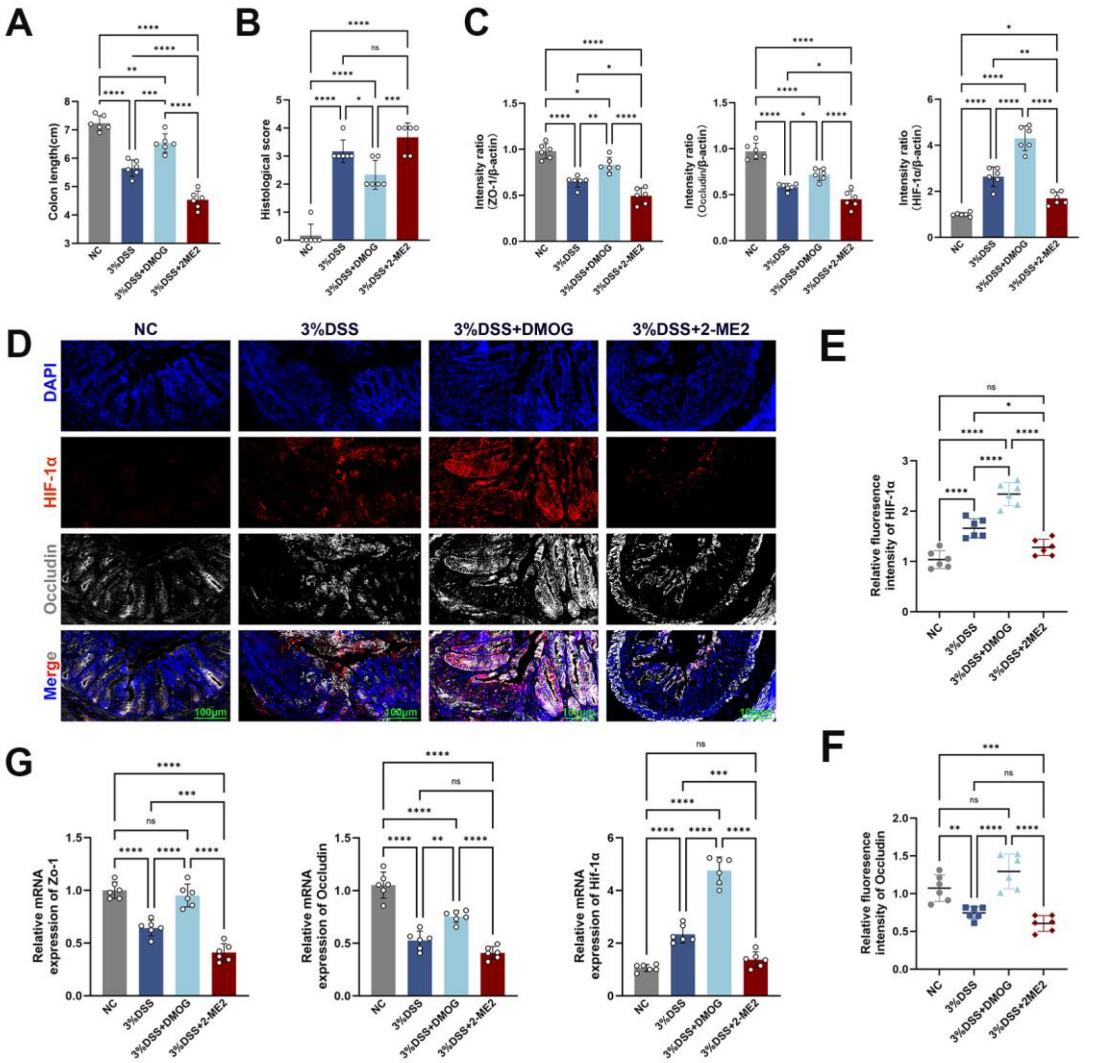

Figure S5. The impact of HIF-1α on essential markers of intestinal barrier in acute colitis mice. (A, B) Colon length measurement and histological scores of murine colon tissues after DSS, DMOG, and 2-ME2 intervention. (C) The quantitative analysis of the protein levels of ZO-1, Occludin, and HIF-1α. (D) Multicolor immunofluorescence staining of HIF-1α, and Occludin on mice colonic sections. Nuclei were stained with DAPI in blue, HIF-1α localization was indicated in red, Occludin staining was indicated in grey (Scale: 100μm). (E, F) Fluorescence intensity of HIF-1α, and Occludin on colon tissues under different intervention strategies. (G) The mRNA expression levels of ZO-1, Occludin and HIF-1α were detected by RT-qPCR in colon tissues. ns $P > 0.05$, *$P < 0.05$, **$P < 0.01$, ***$P < 0.001$, ****$P < 0.0001$.

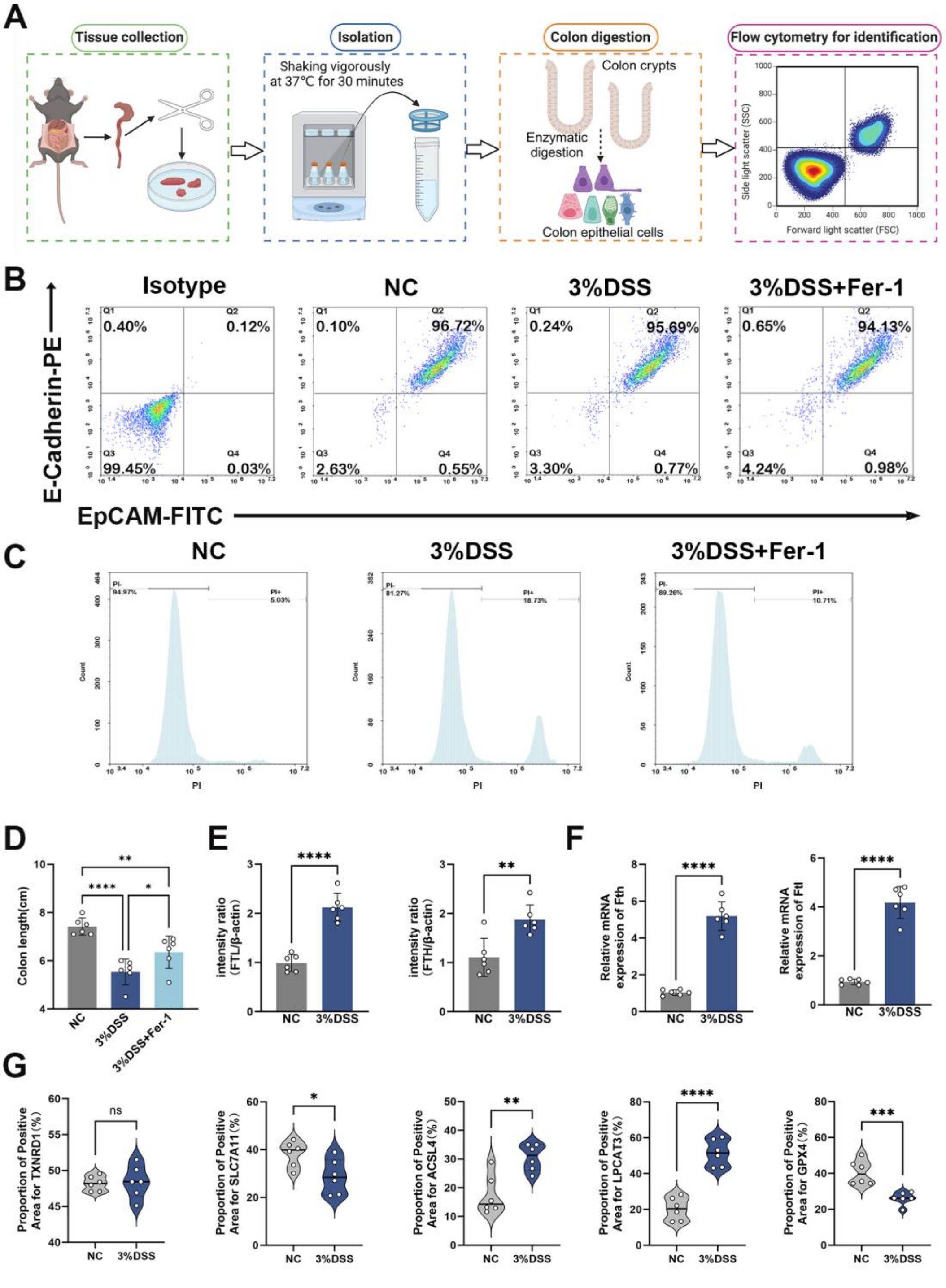

Figure S6. Ferroptosis was induced in murine acute colitis. (A) Schematic overview for the isolation of intestinal epithelial cells. (B) Flow cytometry analyzing the expression of differentiation markers EpCAM and E-Cadherin in the mouse IECs using isotype control antibodies, EpCAM-FITC and E-Cadherin-PE antibodies. (C) Flow cytometry detection of PI-positive cells to analyze necrotic cell death of colon epithelial tissues in mice. (D) Colon length measurement was performed after DSS, and Fer-1 intervention *in vivo*. (E) The quantitative analysis of the protein levels of FTL, and FTH.

(F) The mRNA expression levels of Fth, and Ftl in mouse colon tissues were assessed by RT-qPCR.

(G) Immunohistochemistry slides were evaluated to determine the percentage of positive areas for TXNRD1, SLC7A11, ACSL4, LPCAT3, and GPX4 in mouse acute colitis tissues using ImageJ software. ns $P > 0.05$, *$P < 0.05$, **$P < 0.01$, ***$P < 0.001$, ****$P < 0.0001$.

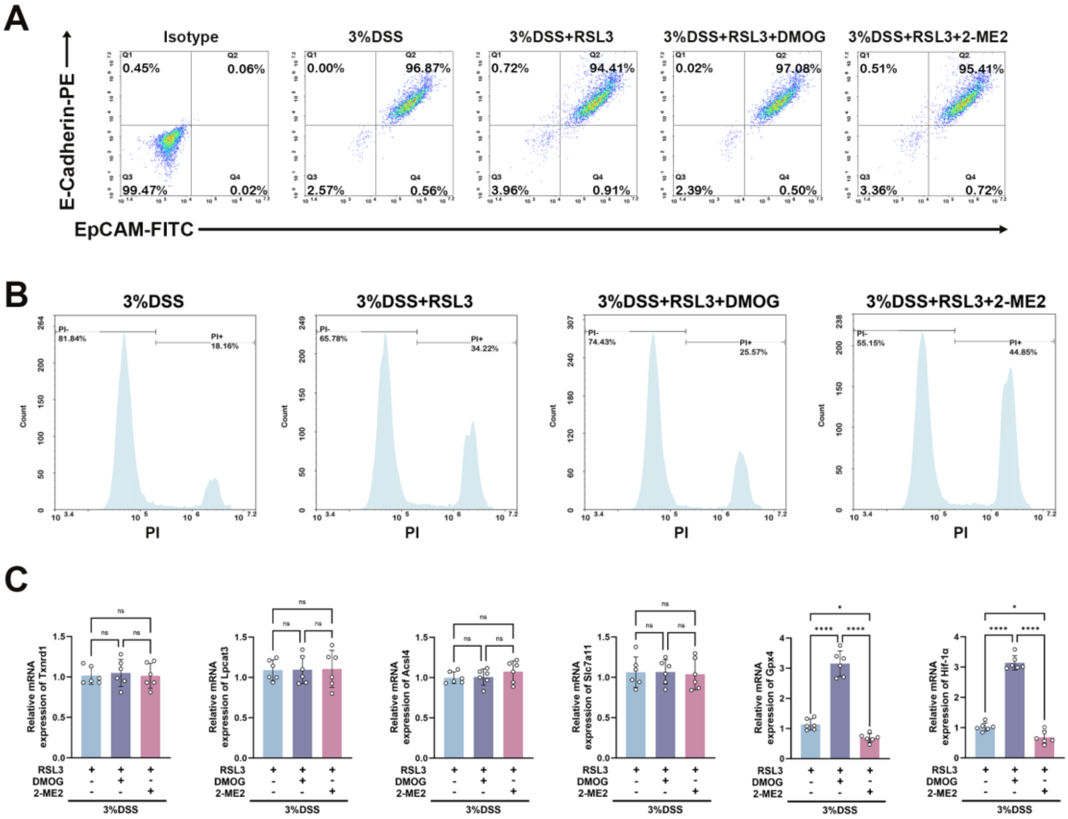

Figure S7. HIF-1α ameliorates colonic epithelial cell death in mice with colitis by upregulating GPX4 expression. (A) Flow cytometry analyzing the expression of differentiation markers EpCAM and E-Cadherin in the mouse IECs using isotype control antibodies, EpCAM-FITC and E-Cadherin-PE antibodies. (B) Flow cytometry detection of PI-positive cells to analyze necrotic cell death of colon epithelial tissues in mice. (C) The mRNA expression levels of Txnrd1, Lpcat3, Acsl4, Slc7a11, Gpx4, and Hif-1α in mouse colon tissues were assessed by RT-qPCR. ns $P > 0.05$, *$P < 0.05$, ****$P < 0.0001$.

Supplementary Tables

Table S1. The shRNA sequences for HIF-1A

sh-HIF1A	sense (5'-3')	anti-sense (5'-3')
sh-RNA 1	GATCCGCAGTGTGGCTACAAGAAAC CTTCAAGAGAGGTTTCTTGTAGCCA CACTGCTTTTTCTCGAGG	AATTCCTCGAGAAAAAGCAGTG TGGCTACAAGAAACCTCTCTTGA AGGTTTCTTGTAGCCACACTGCG
sh-RNA 2	GATCCGCATTGAAGTTAGAGTCAAG CTTCAAGAGAGCTTGACTCTAACTT CAATGCTTTTTCTCGAGG	AATTCCTCGAGAAAAAGCATTG AAGTTAGAGTCAAGCTCTCTTGA AGCTTGACTCTAACTTCAATGCG
sh-RNA 3	GATCCGCAGTGACGAAGGACAATAT ATTCAAGAGATATATTGTCCTTCGTC ACTGCTTTTTCTCGAGG	AATTCCTCGAGAAAAAGCAGTG ACGAAGGACAATATATCTCTTGAA TATATTGTCCTTCGTCACTGCG
sh-NC	GATCCGTTCTCCGAACGTGTCACGTA ATTCAAGAGATTACGTGACACGTTC GGAGAATTTTTC	AATTGAAAAAATTCTCCGAACGTG TCACGTAATCTCTTGAATTACGTG ACACGTTCGGAGAACG

Table S2. Primer sequences for RT-qPCR

Gene	Forward (5'-3')	Reverse (5'-3')
β-actin	CACCATTGGCAATGAGCGGTTC	AGGTCTTTGCGGATGTCCACGT
HIF-1α	TATGAGCCAGAAGAACTTTTAGGC	CACCTCTTTTGGCAAGCATCCTG
FTH	TGAAGCTGCAGAACCAACGAGG	GCACACTCCATTGCATTCAGCC
FTL	TACGAGCGTCTCCTGAAGATGC	GGTTCAGCTTTTTCTCCAGGGC
GPX4	ACAAGAACGGCTGCGTGGTGAA	GCCACACACTTGTGGAGCTAGA
SLC7A11	TCCTGCTTTGGCTCCATGAACG	AGAGGAGTGTGCTTGCGGACAT
TXNRD1	GTTACTTGGGCATCCCTGGTGA	CGCACTCCAAAGCGACATAGGA
LPCAT3	CAGGATACCTGGTCTGCTTCCA	TGAAGAGCCAGTGGATGGTCTG
ACSL4	GCTATCTCCTCAGACACACCGA	AGGTGCTCCAACTCTGCCAGTA
IL-1β	CCACAGACCTTCCAGGAGAATG	GTGCAGTTCAGTGATCGTACAGG
IL-6	AGACAGCCACTCACCTCTTCAG	TTCTGCCAGTGCCTCTTTGCTG
mus-β-actin	CATTGCTGACAGGATGCAGAAGG	TGCTGGAAGGTGGACAGTGAGG
mus-Hif-1α	CCTGCACTGAATCAAGAGGTTGC	CCATCAGAAGGACTTGCTGGCT
mus-Fth	GCCGAGAAACTGATGAAGCTGC	GCACACTCCATTGCATTCAGCC
mus-Ftl	CCTCGAGTTTCAGAACGATCGC	CCTGATTCAGGTTCTTCTCCATG
mus-Gpx4	CCTCTGCTGCAAGAGCCTCCC	CTTATCCAGGCAGACCATGTGC
mus-Slc7a11	CTTTGTTGCCCTCTCCTGCTTC	CAGAGGAGTGTGCTTGTGGACA
mus-Txnrd1	AGTCACATCGGCTCGCTGAACT	GATGAGGAACCGCTCTGCTGAA
mus-Lpcat3	CCATCTCTTCCACACCTTCACG	GGATGAGGAACTGAAGCACGAC
mus-Acsl4	CCTTTGGCTCATGTGCTGGAAC	GCCATAAGTGTGGGTTTCAGTAC
mus-Il-β	TGGACCTTCCAGGATGAGGACA	GTTCATCTCGGAGCCTGTAGTG
mus-Il-6	TACCACTTCACAAGTCGGAGGC	CTGCAAGTGCATCATCGTTGTTC
mus-Zo-1	GTTGGTACGGTGCCCTGAAAGA	GCTGACAGGTAGGACAGACGAT

mus-Occludin	TGGCAAGCGATCATACCCAGAG	CTGCCTGAAGTCATCCACACTC

Table S3. The primer sequences for the GPX4 promoter

GPX4	Forward (5'-3')	Reverse (5'-3')
Primer 1	GCTCGTGTAATCCCAGCTACTC	CCCAAGCCCCTACGCAGAAAGA
Primer 2	CCAACATGGTGAAAGCCCGTA	TTTTCACTTCGTGGCTTGGACA
Primer 3	CCAAACCATCCATGACGCCTCT	CCGCCTAGGTGCTTGGGATTTGT
