# Supplementary material for: HIF-1α alleviates ferroptosis in ulcerative colitis by regulation of GPX4
Source: Cell Death Dis. 2025 Jul 22;16(1):542. doi: 10.1038/s41419-025-07883-8 (PMC12280037; doi:10.1038/s41419-025-07883-8)

Western blot original images

**Figure 1D**

FTL (NC1-3; UC1-3)

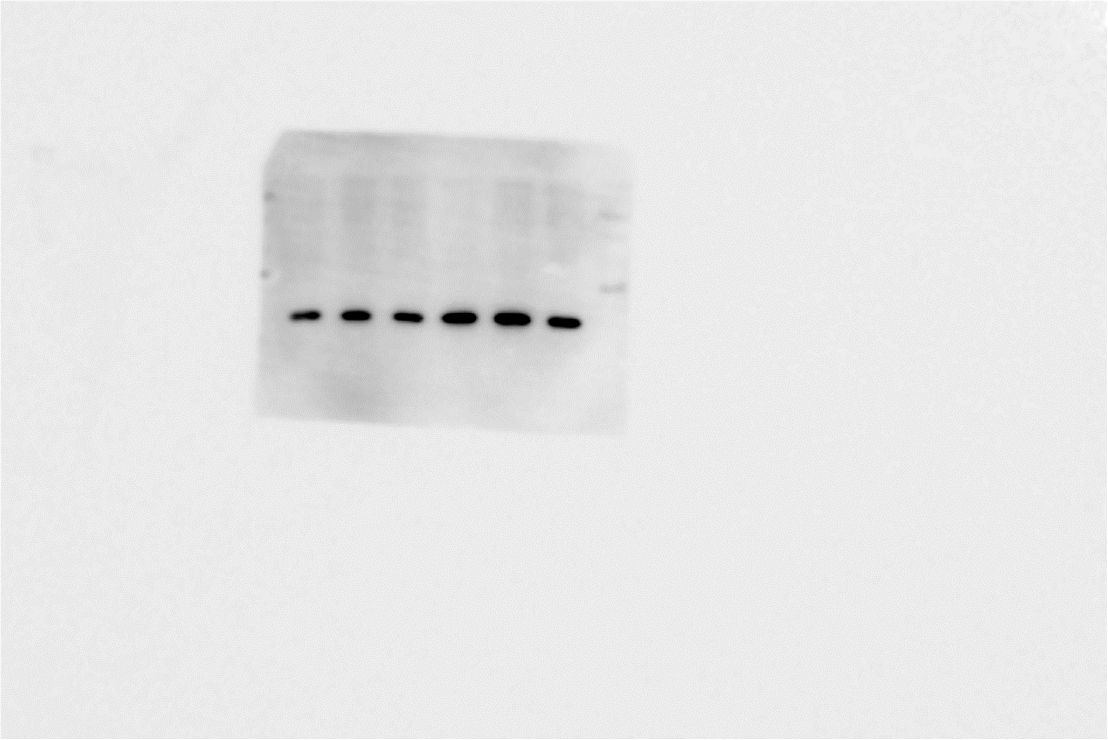

FTH (NC1-3; UC1-3)

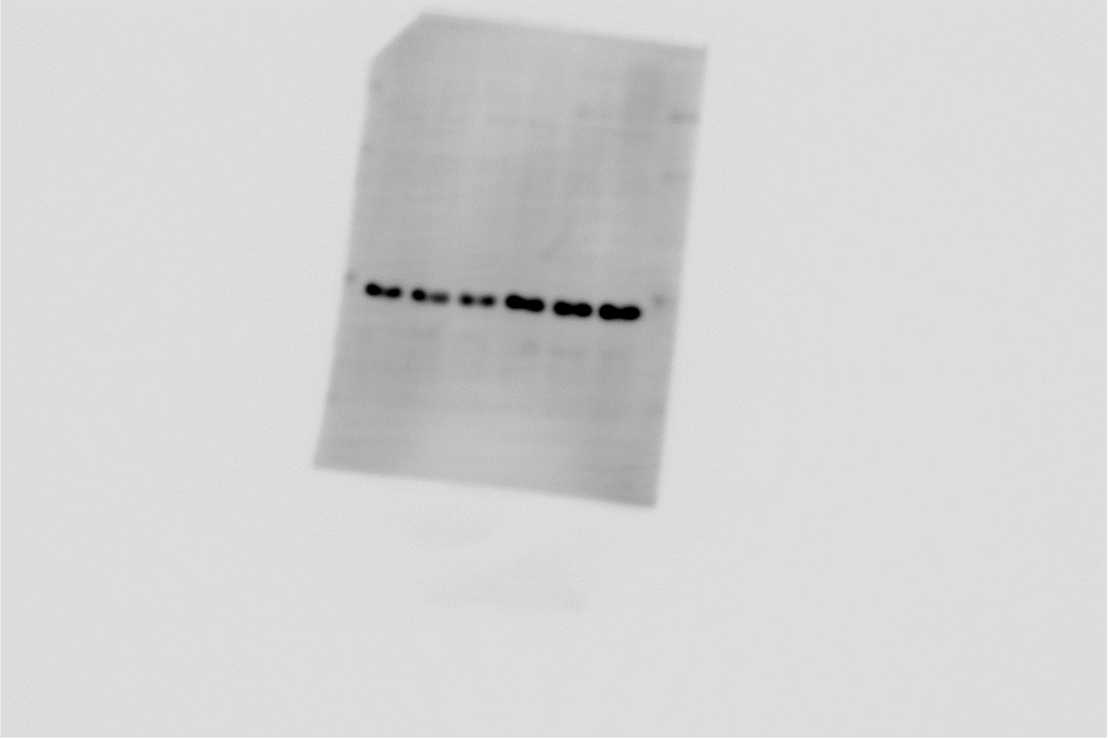

**Figure 2A**

HIF-1α (NC: UC 1-4#)

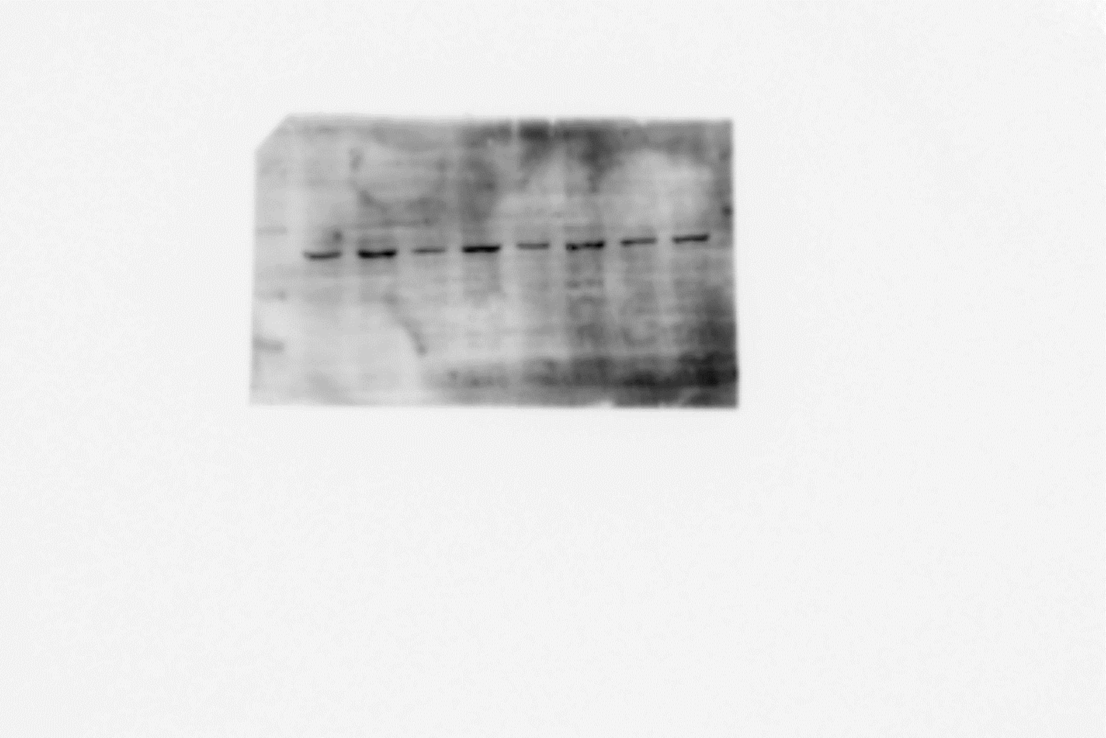

β-actin (NC: UC 1-4#)

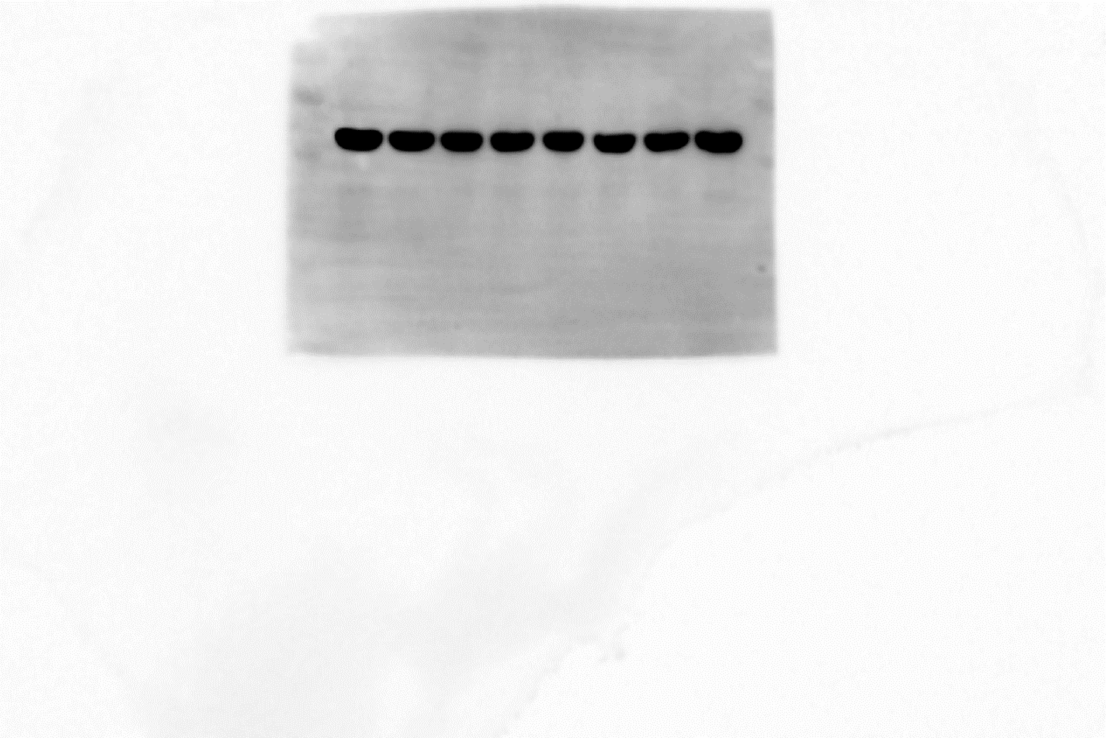

HIF-1α (NC: UC 5-8#)

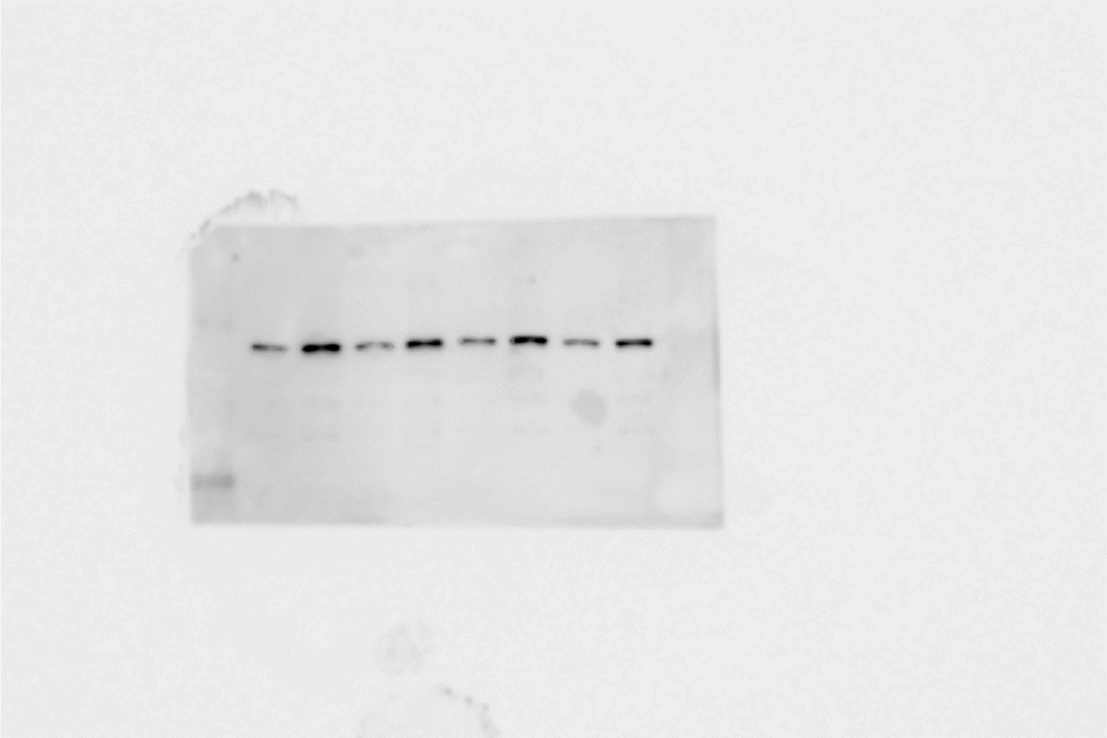

β-actin (NC: UC 5-8#)

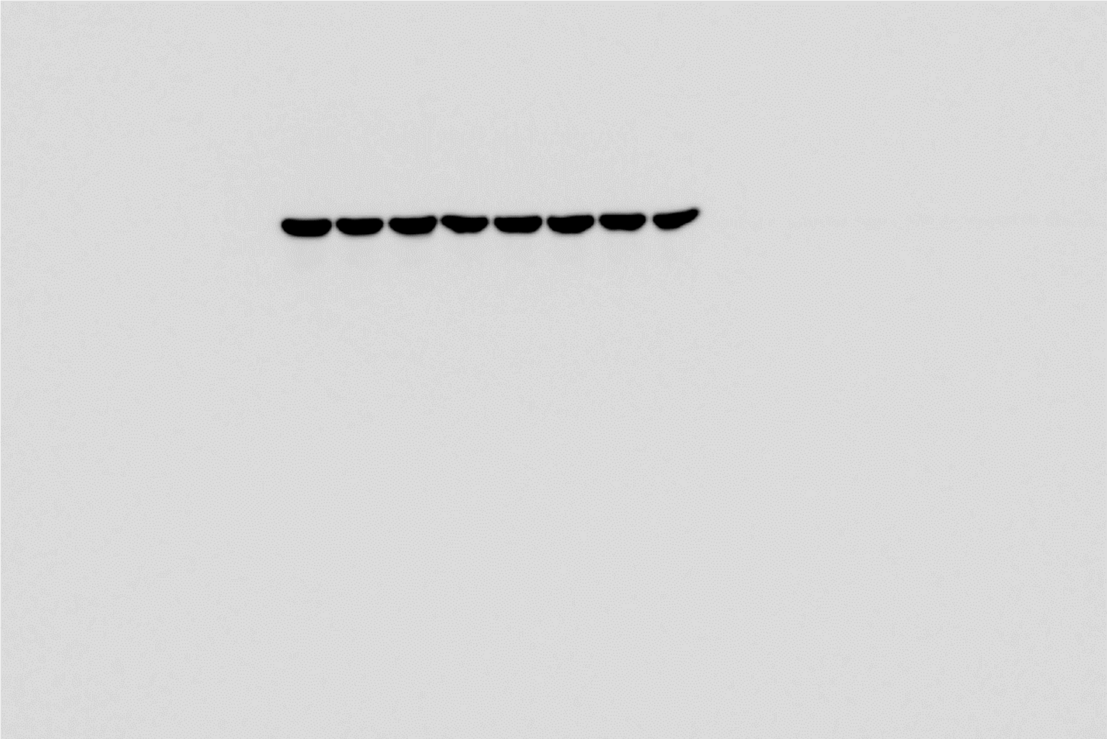

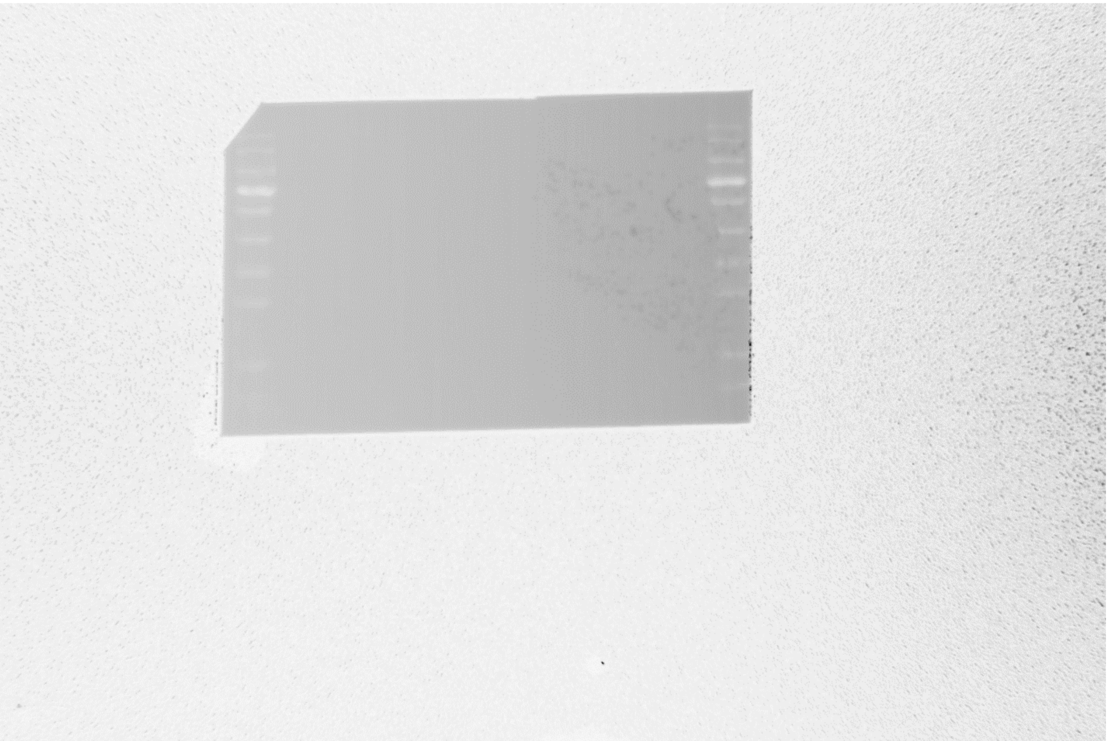

**Figure 3B**

HIF-1α (WT/Vector/HIF1A-OE)

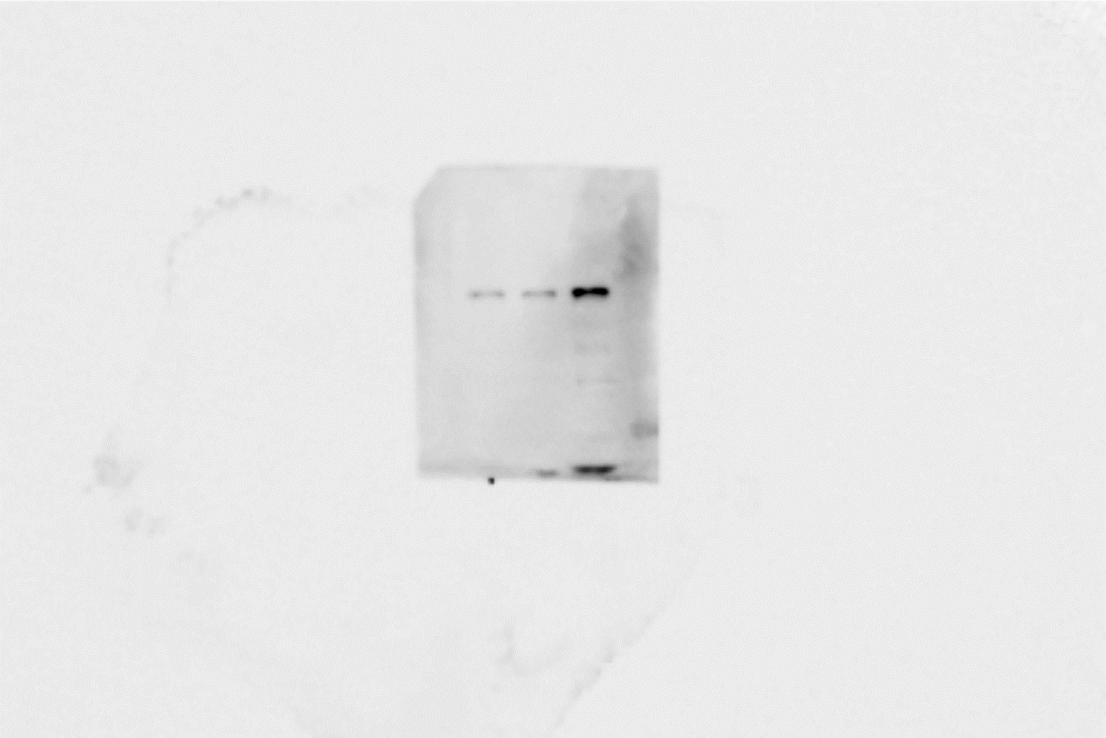

β-actin (WT/Vector/HIF1A-OE)

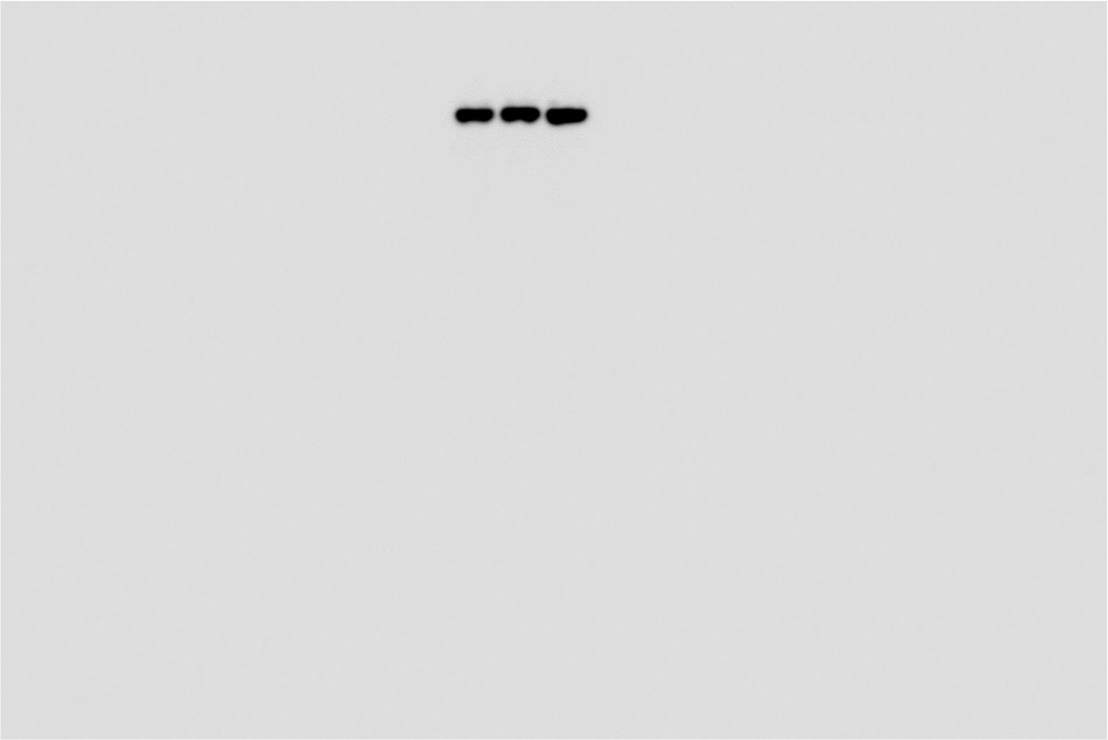

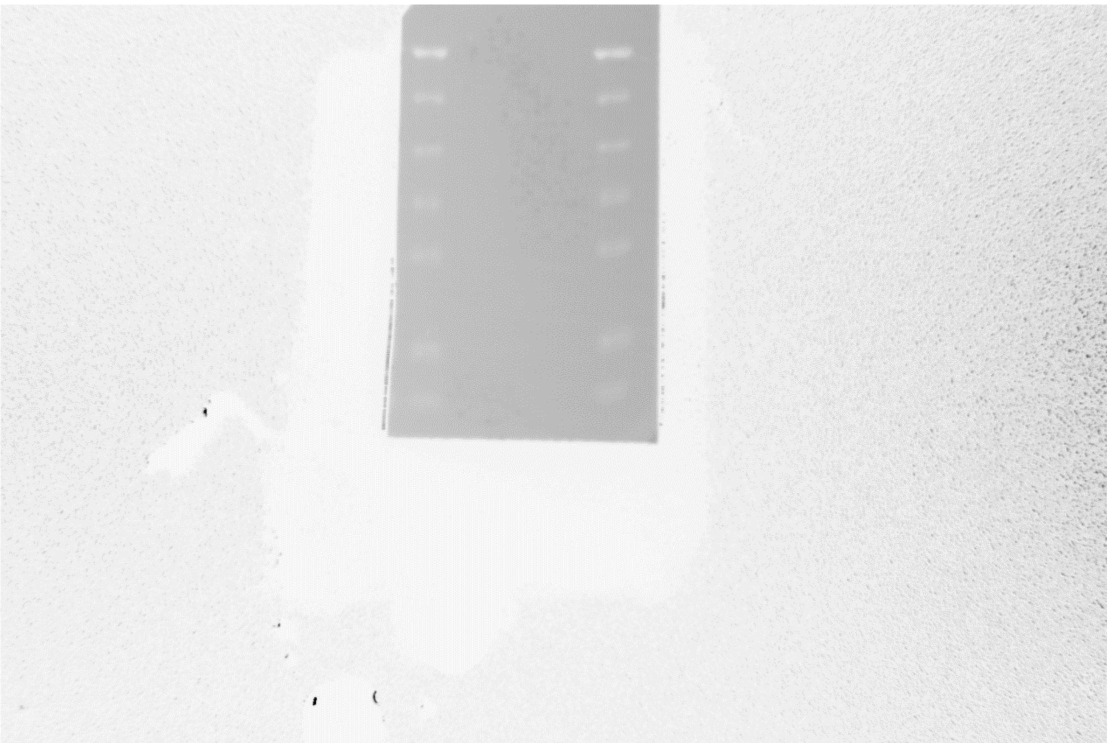

**Figure 3D**

Zonulin(Vector /LPS- +; HIF1A-OE/ LPS- +)

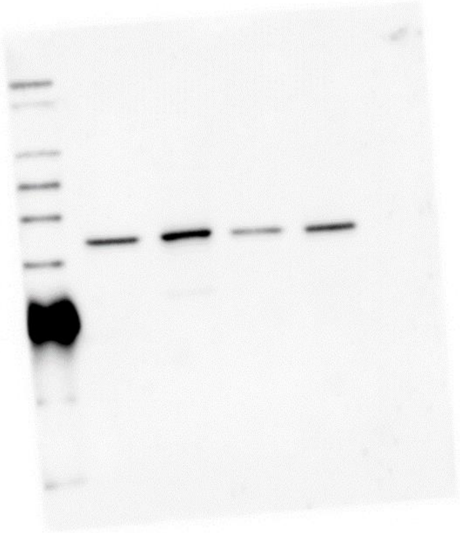

Occludin (Vector /LPS- +; HIF1A-OE/ LPS- +)

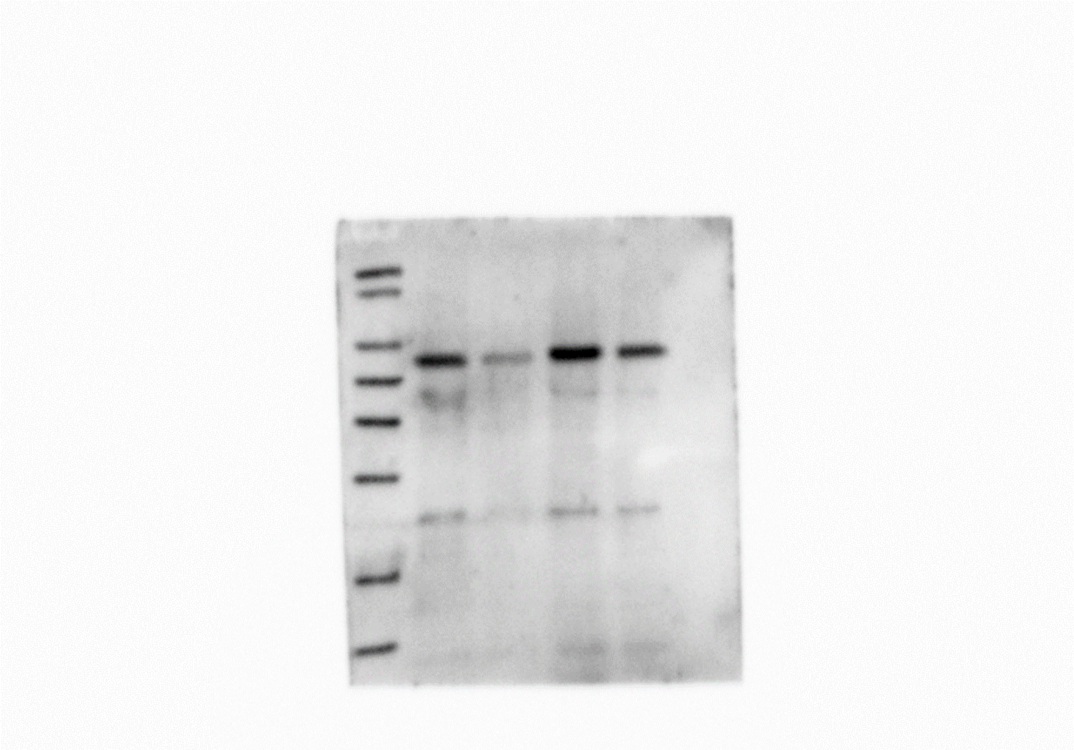

ZO-1 (Vector /LPS- +; HIF1A-OE/ LPS- +)

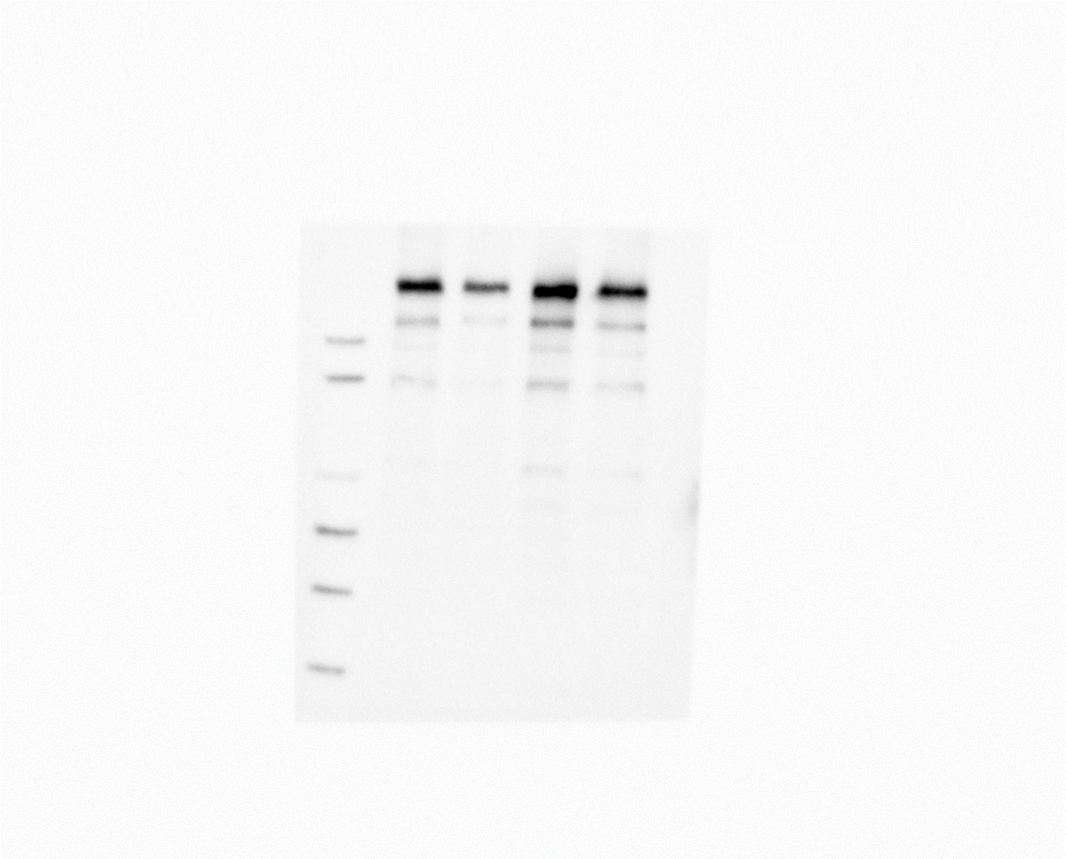

HIF-1α (Vector /LPS- +; HIF1A-OE/ LPS- +)

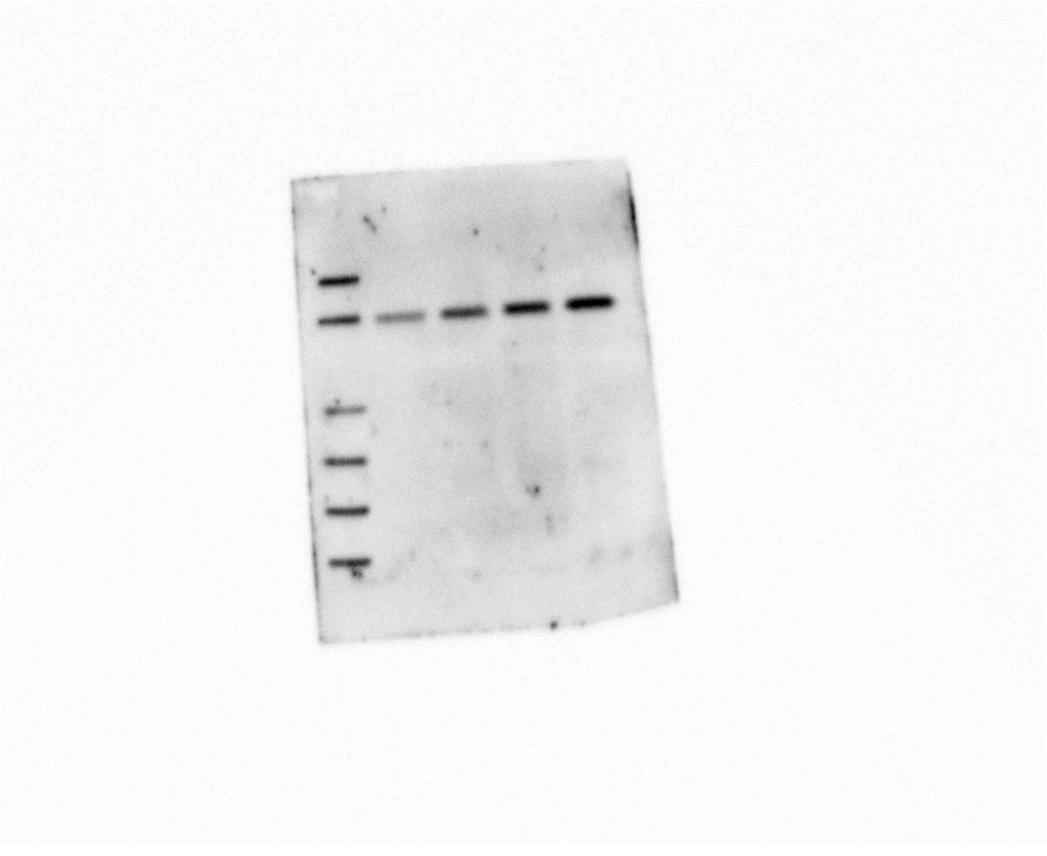

β-actin (Vector /LPS- +; HIF1A-OE/ LPS- +)

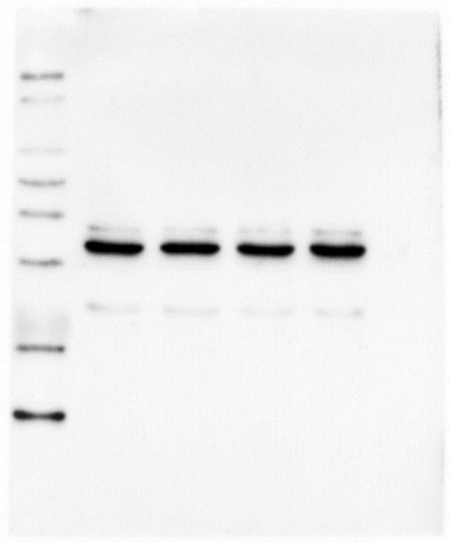

**Figure 3K**

FTL (Vector /LPS- +; HIF1A-OE/ LPS- +)

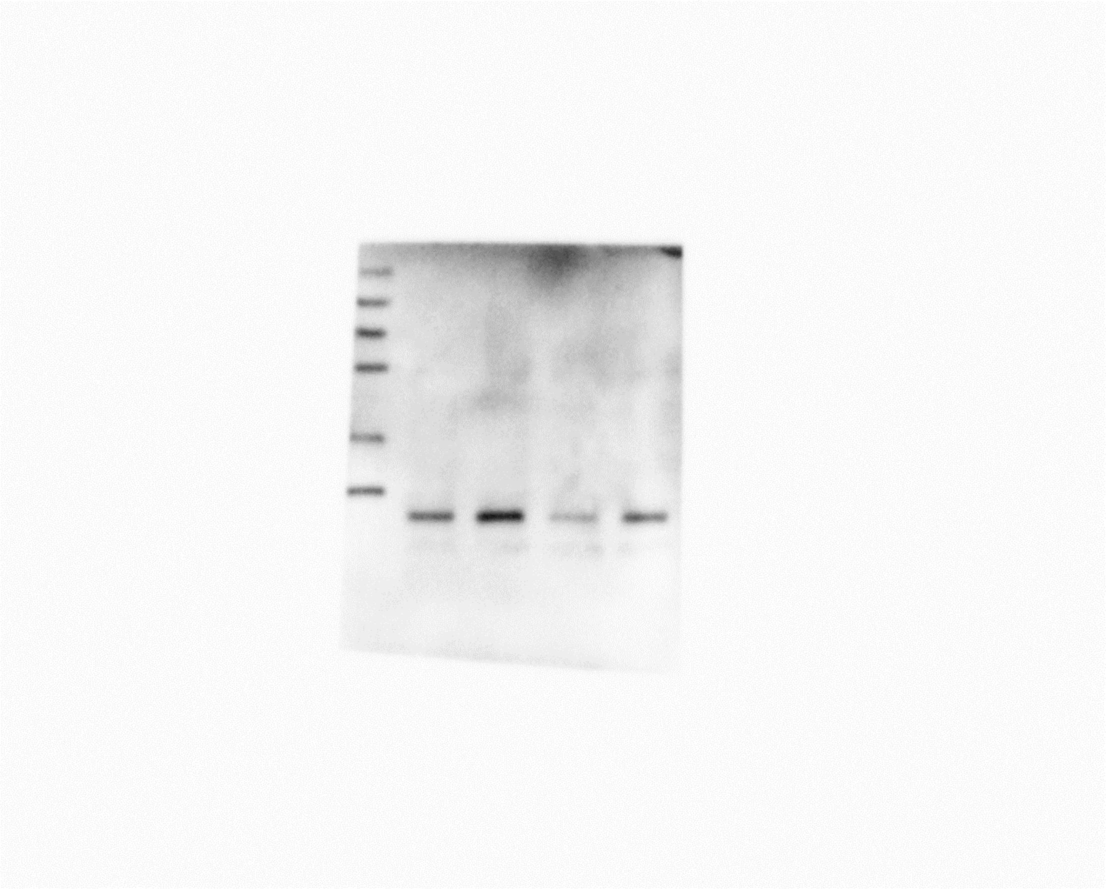

FTH (Vector /LPS- +; HIF1A-OE/ LPS- +)

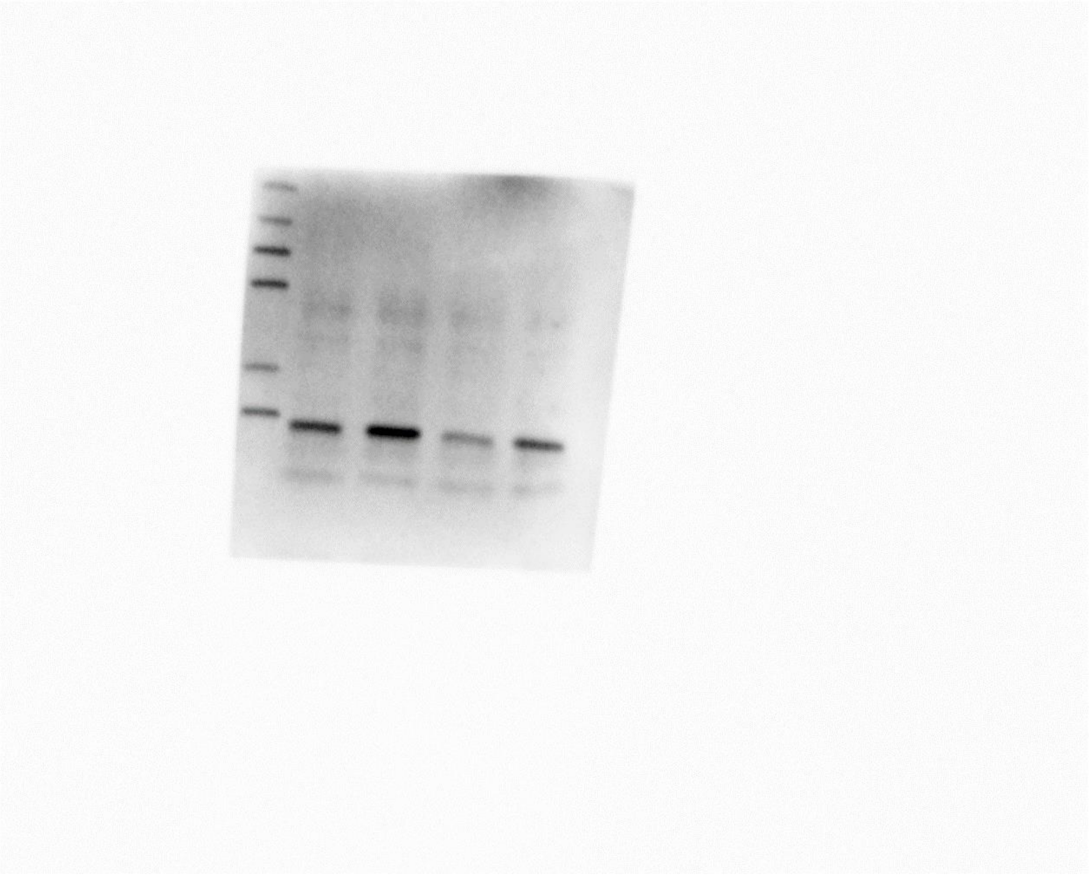

HIF-1α (Vector /LPS- +; HIF1A-OE/ LPS- +)

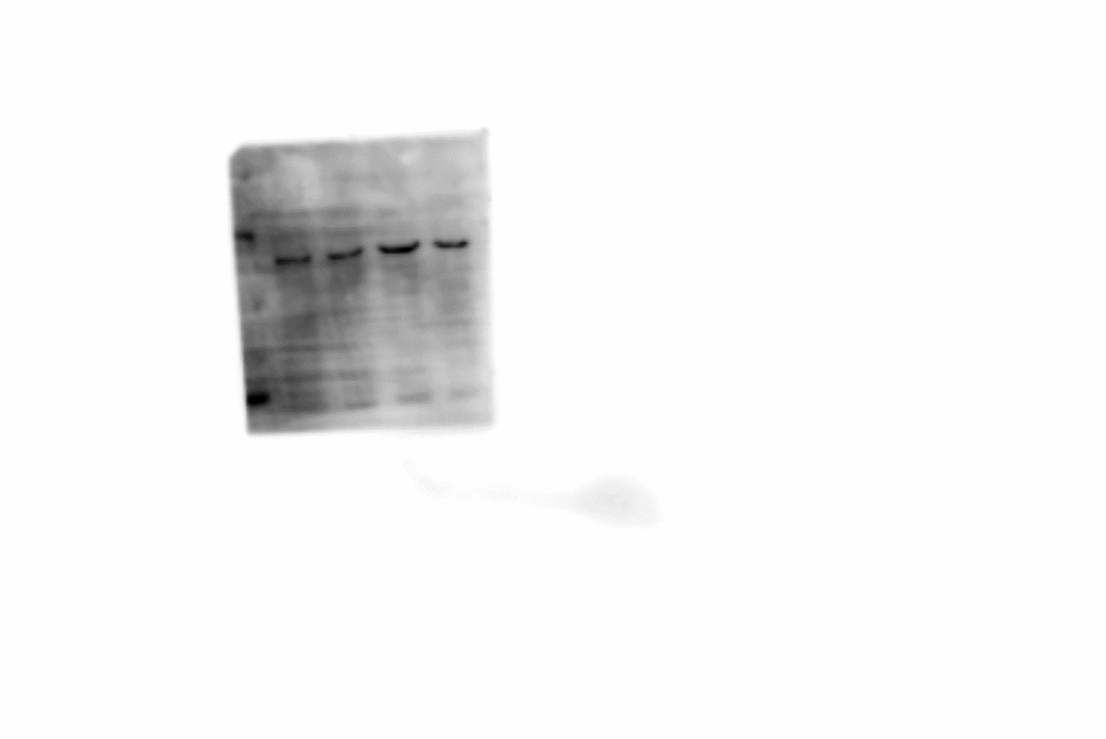

β-actin (Vector /LPS- +; HIF1A-OE/ LPS- +)

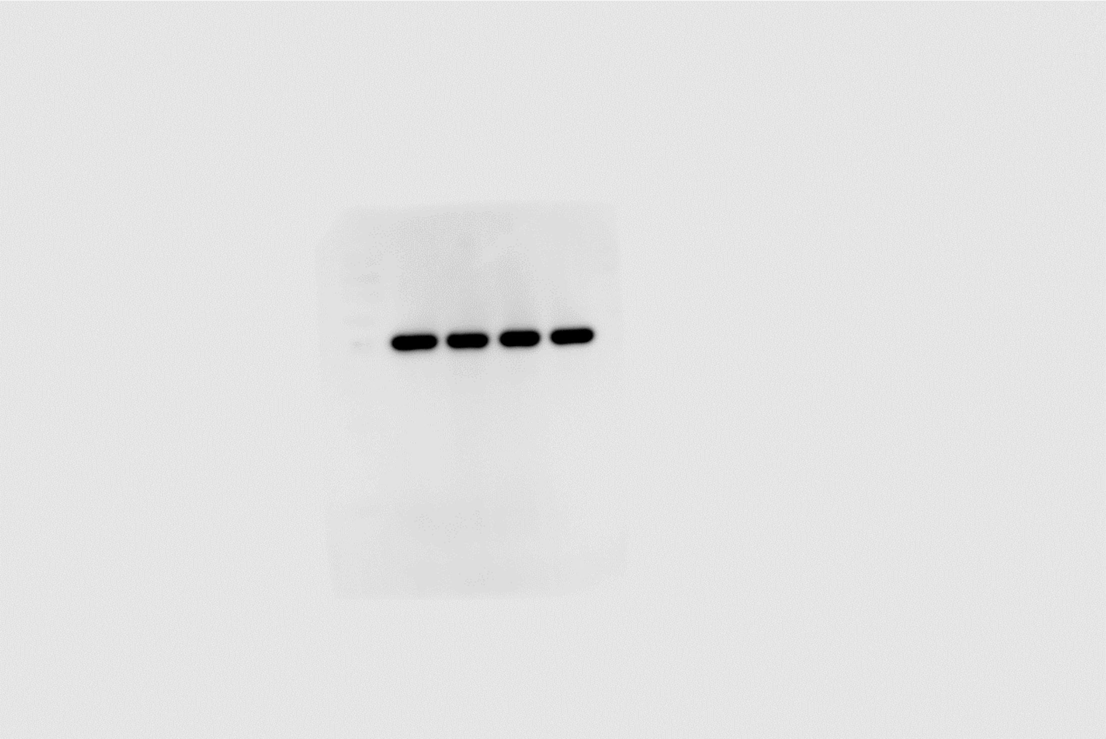

**Figure 3N**

ACSL4 (Vector /LPS- +; HIF1A-OE/ LPS- +)

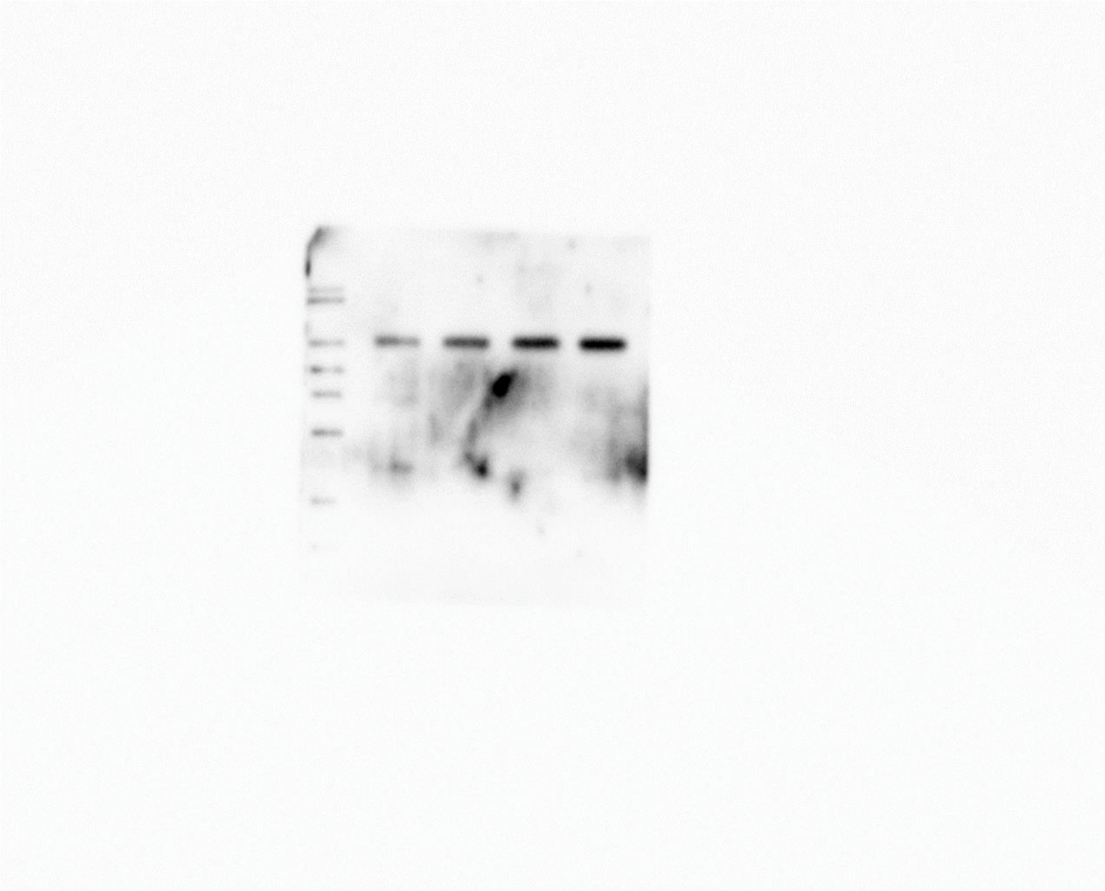

LPCAT3 (Vector /LPS- +; HIF1A-OE/ LPS- +)

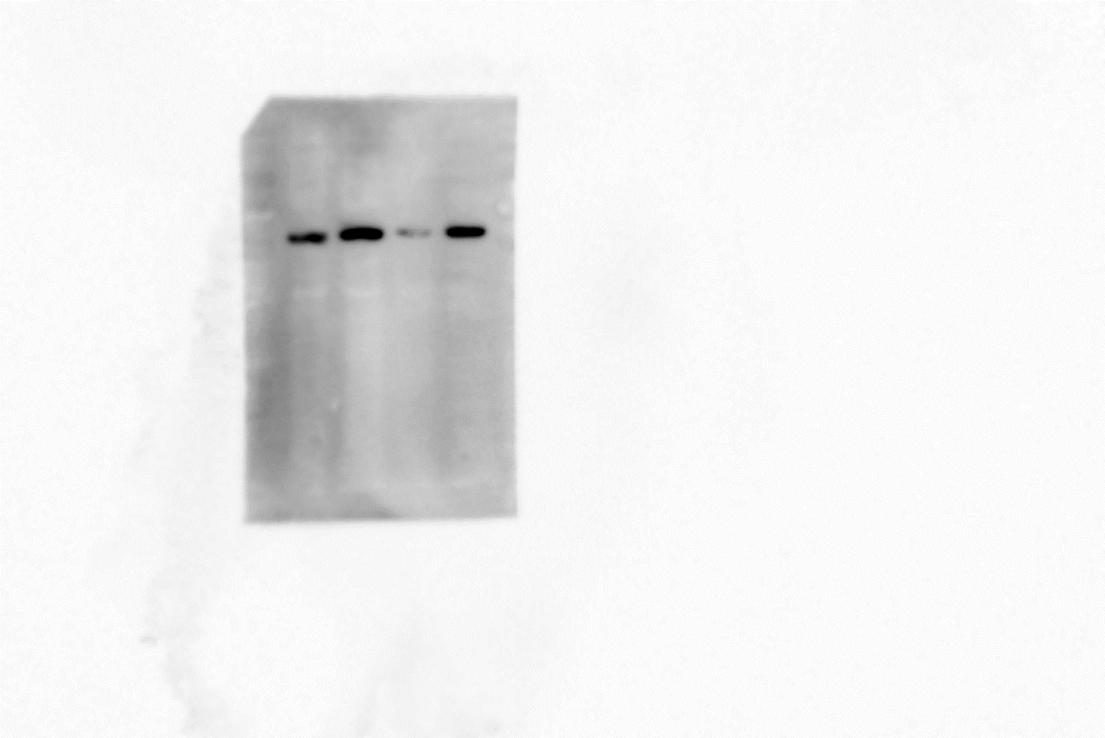

SLC7A11 (Vector /LPS- +; HIF1A-OE/ LPS- +)

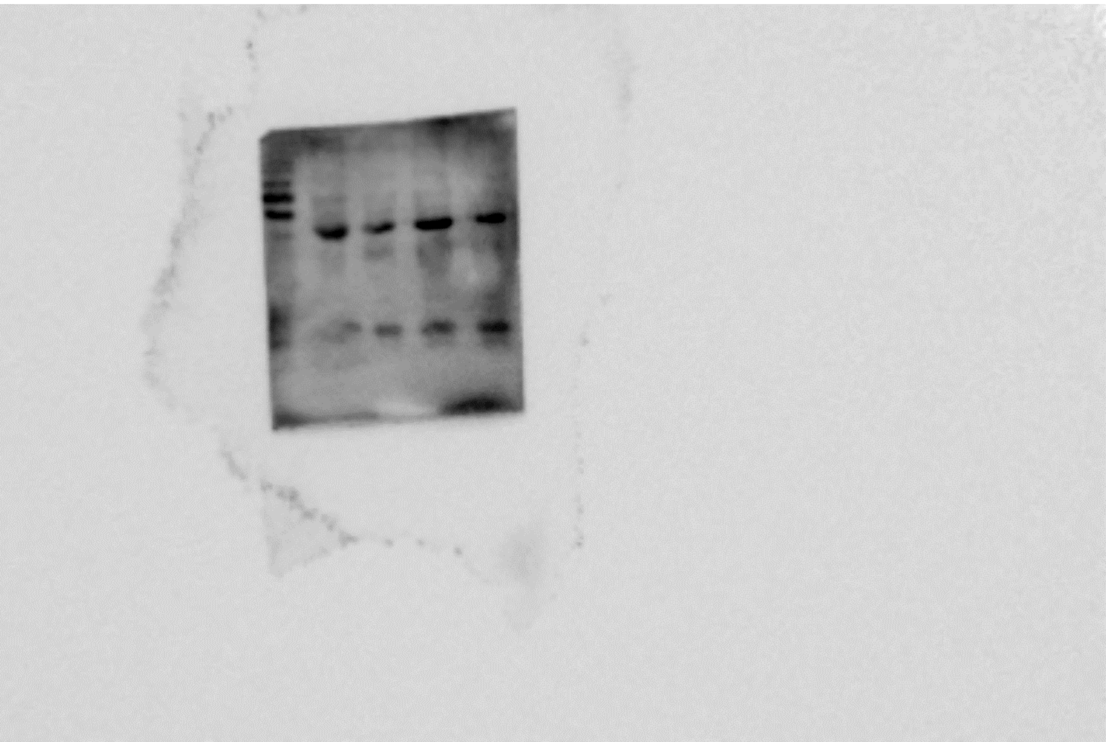

GPX4 (Vector /LPS- +; HIF1A-OE/ LPS- +)

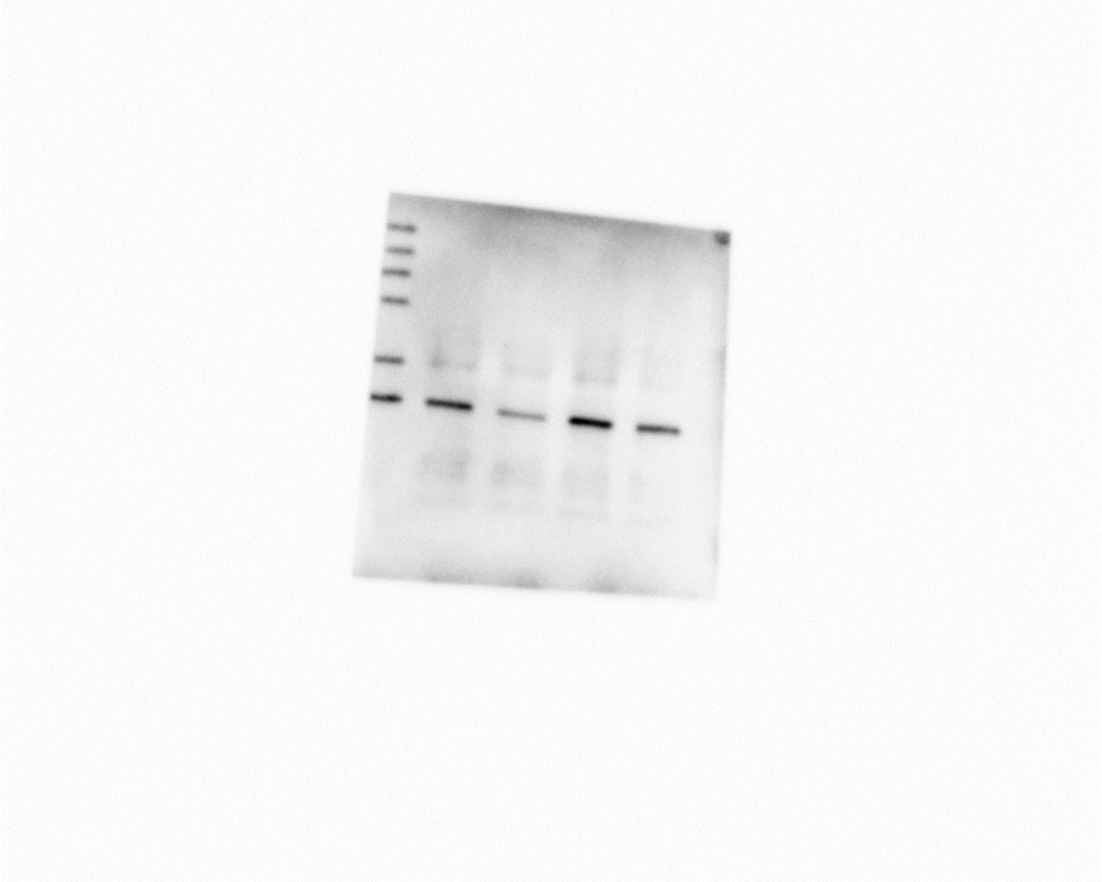

TXNRD1 (Vector /LPS- +; HIF1A-OE/ LPS- +)

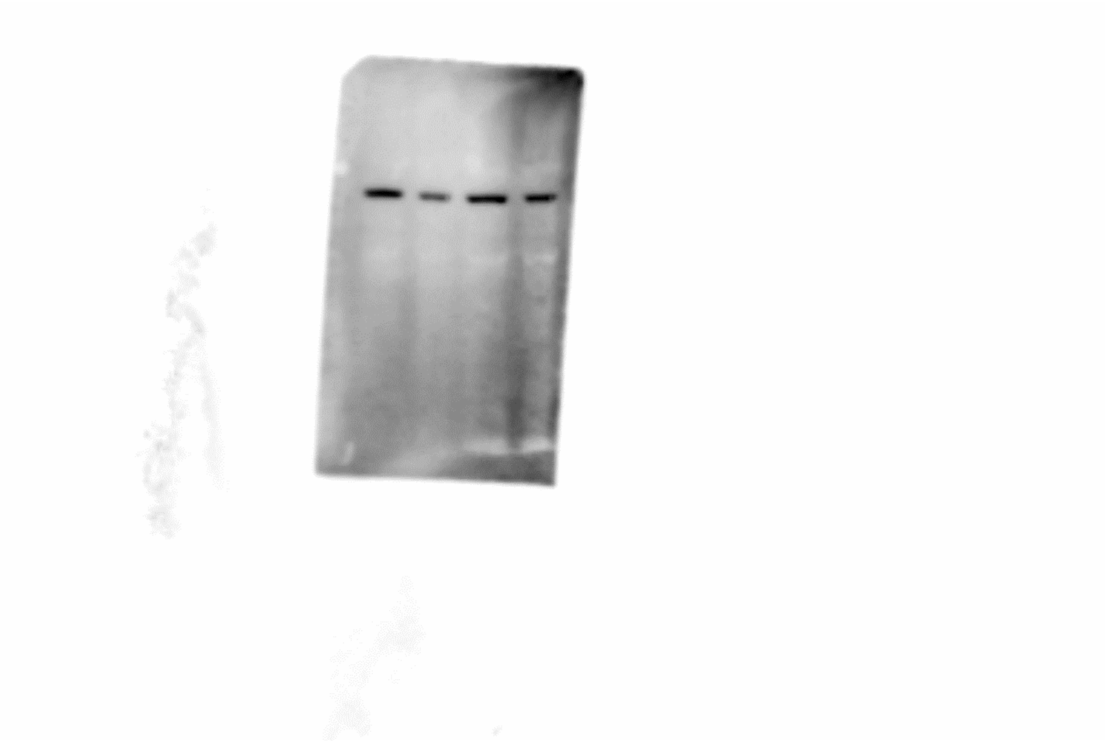

HIF-1α (Vector /LPS- +; HIF1A-OE/ LPS- +)

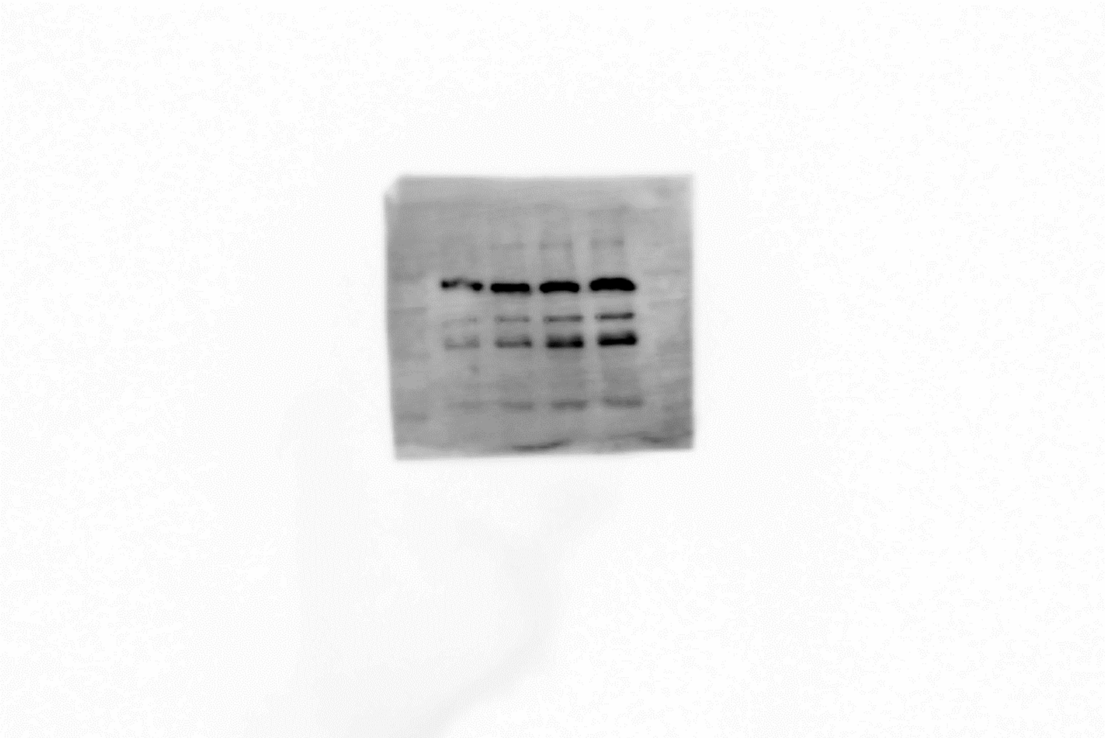

β-actin (Vector /LPS- +; HIF1A-OE/ LPS- +)

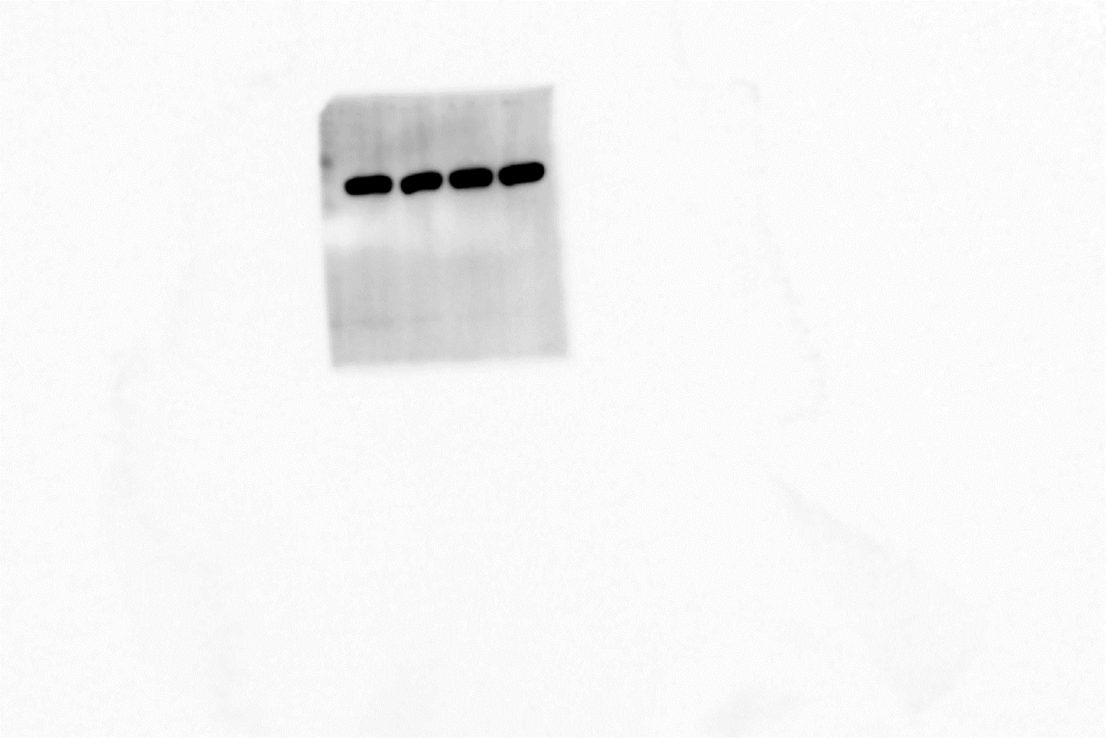

**Figure 4K**

FTL (LPS+Vector/0 2 4 8μM; LPS+HIF1A-OE/0 2 4 8μM)

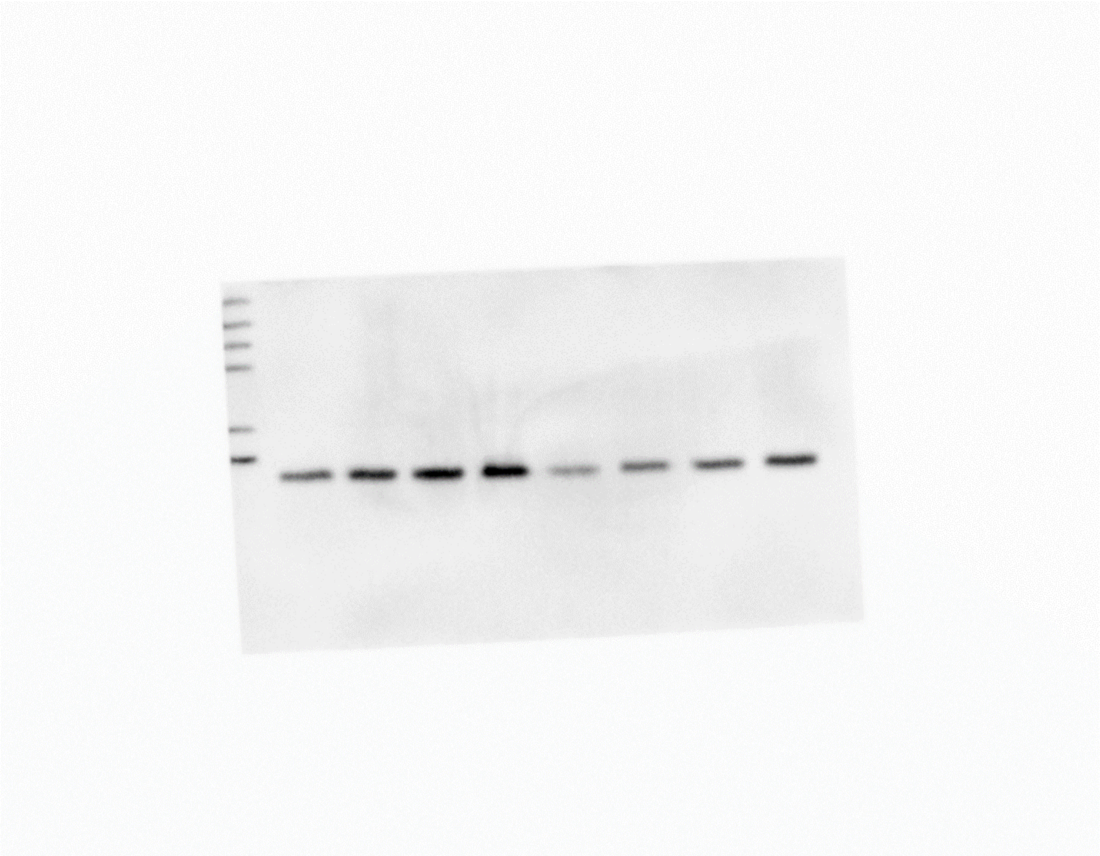

FTH (LPS+Vector/0 2 4 8μM; LPS+HIF1A-OE/0 2 4 8μM)

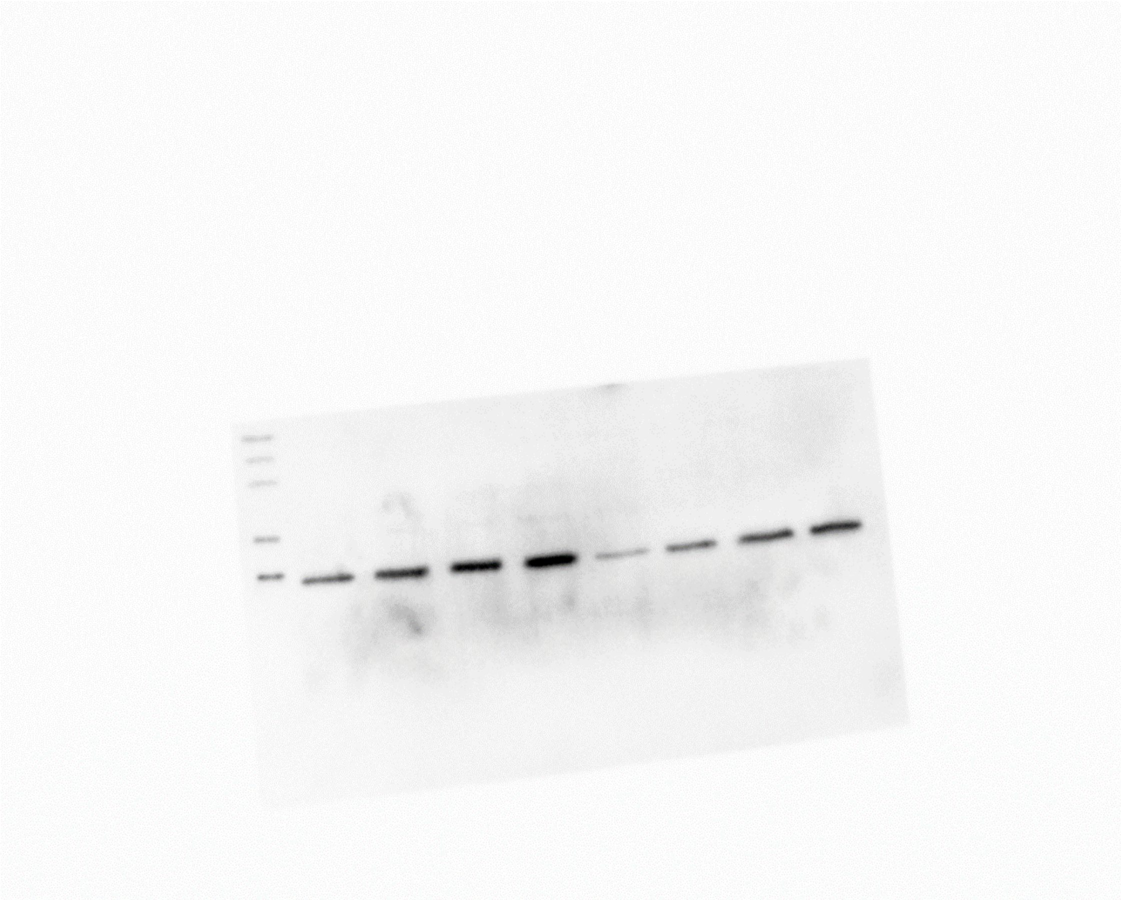

HIF-1α (LPS+Vector/0 2 4 8μM; LPS+HIF1A-OE/0 2 4 8μM)

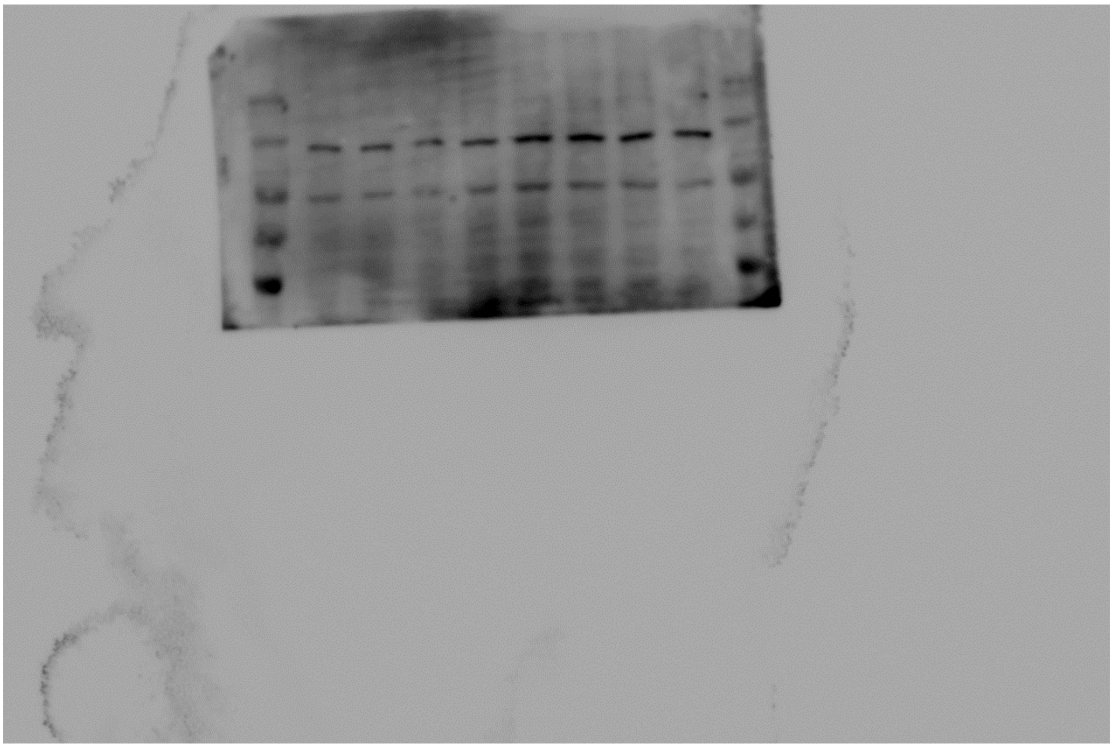

β-actin (LPS+Vector/0 2 4 8μM; LPS+HIF1A-OE/0 2 4 8μM)

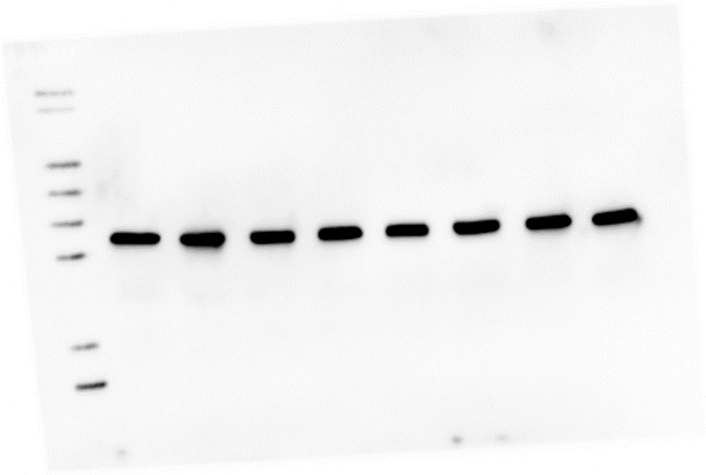

**Figure 4O**

ACSL4 (LPS+Vector/0 2 4 8μM; LPS+HIF1A-OE/0 2 4 8μM)

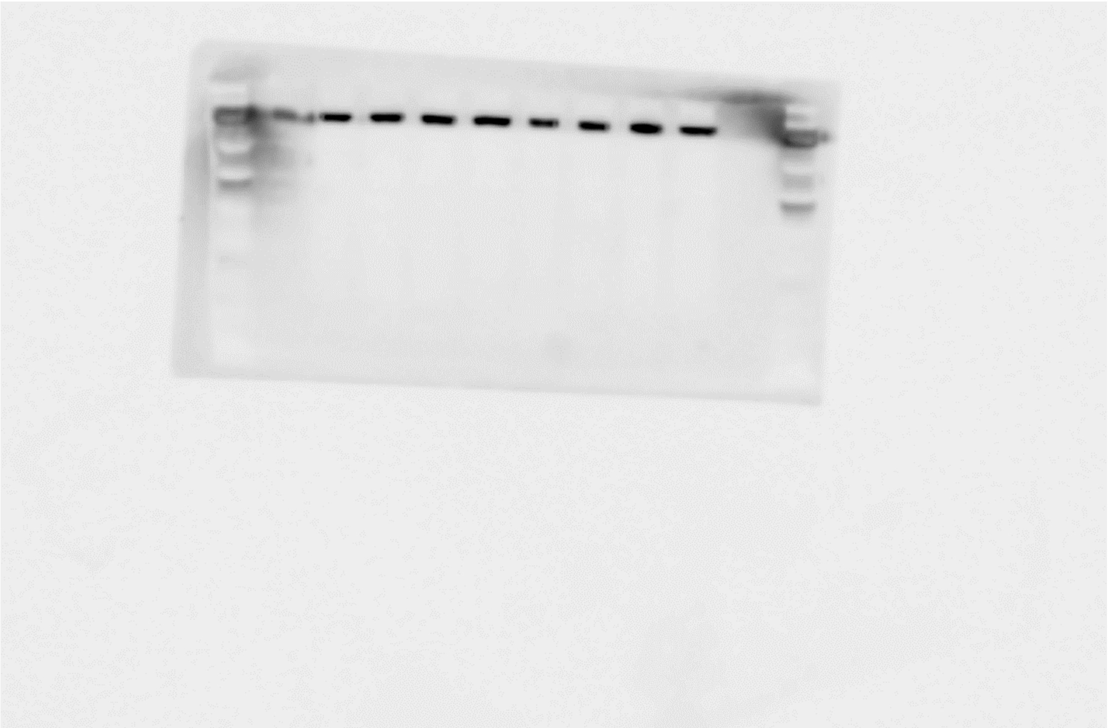

SLC7A11 (LPS+Vector/0 2 4 8μM; LPS+HIF1A-OE/0 2 4 8μM)

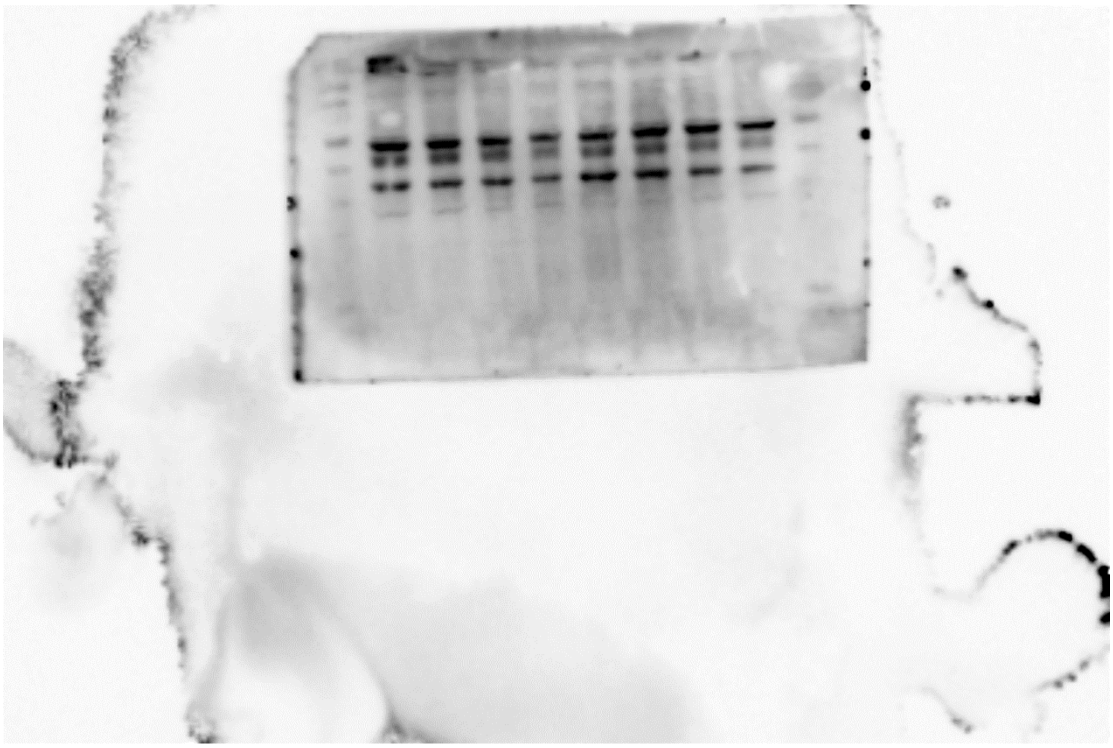

GPX4 (LPS+Vector/0 2 4 8μM; LPS+HIF1A-OE/0 2 4 8μM)

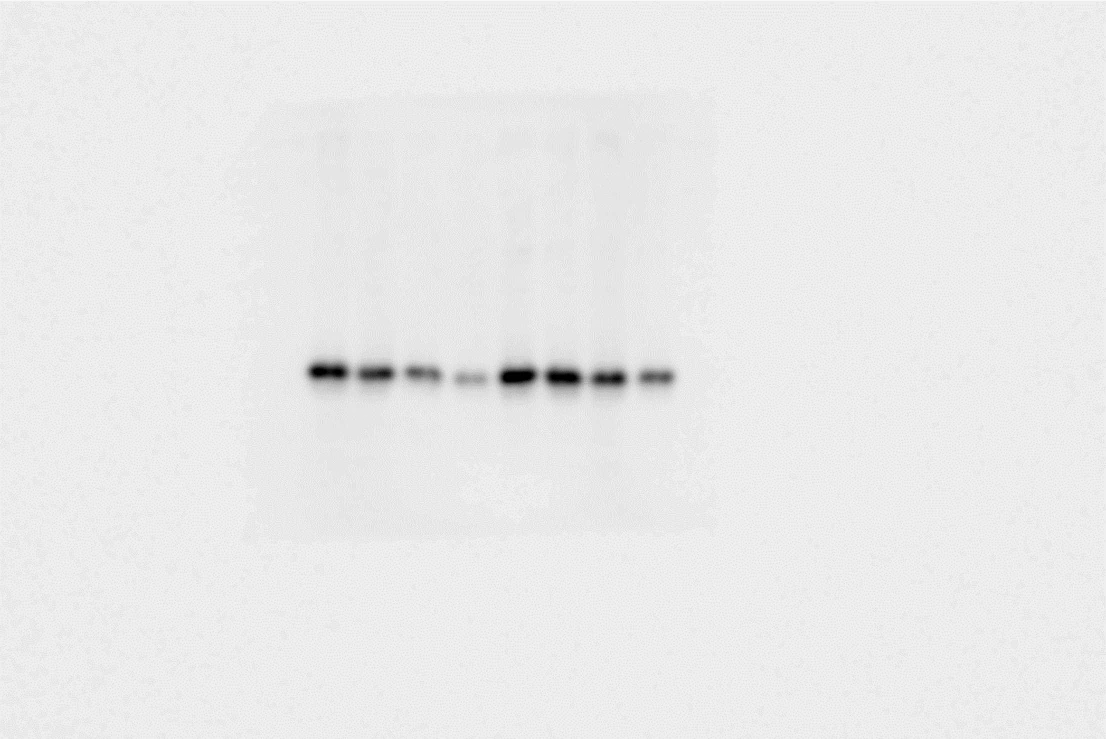

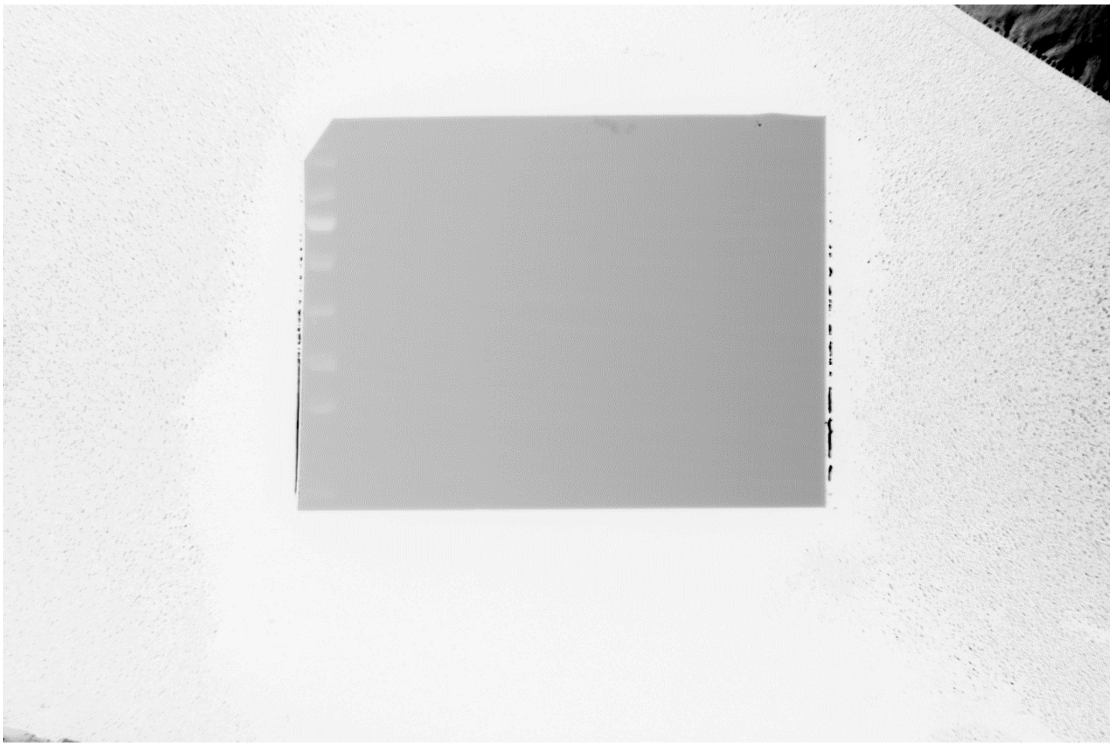

LPCAT3 (LPS+Vector/0 2 4 8μM; LPS+HIF1A-OE/0 2 4 8μM)

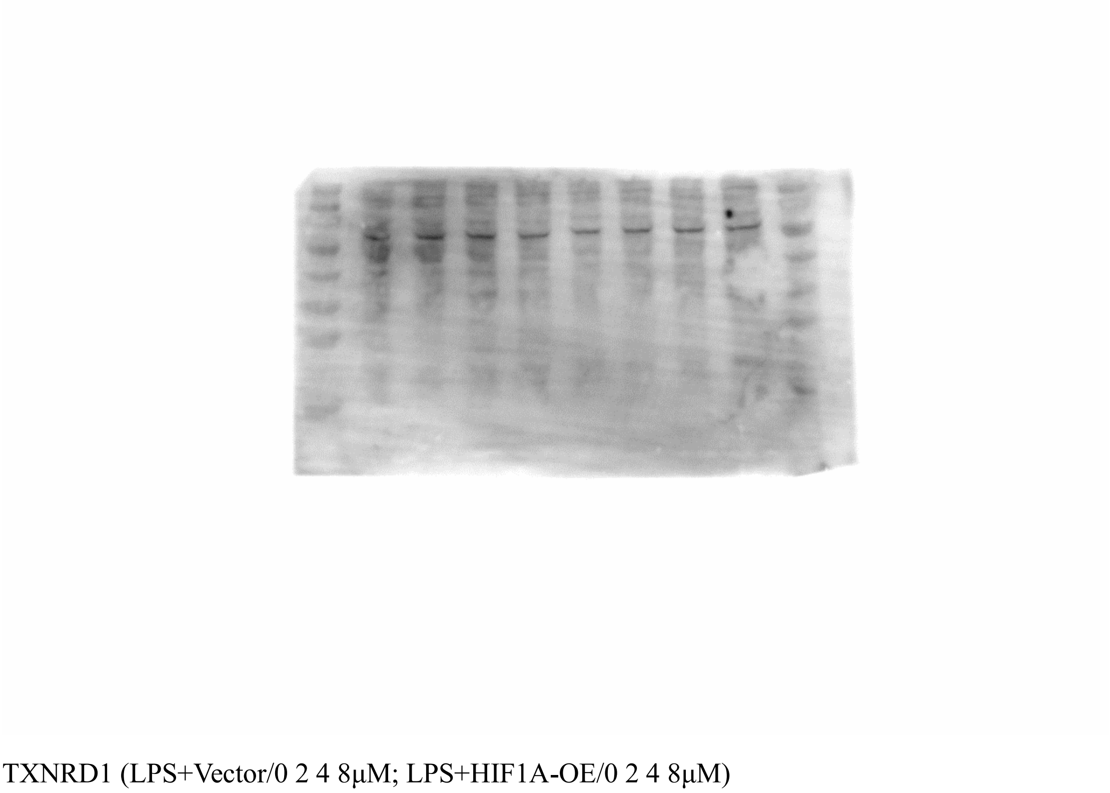

TXNRD1 (LPS+Vector/0 2 4 8μM; LPS+HIF1A-OE/0 2 4 8μM)

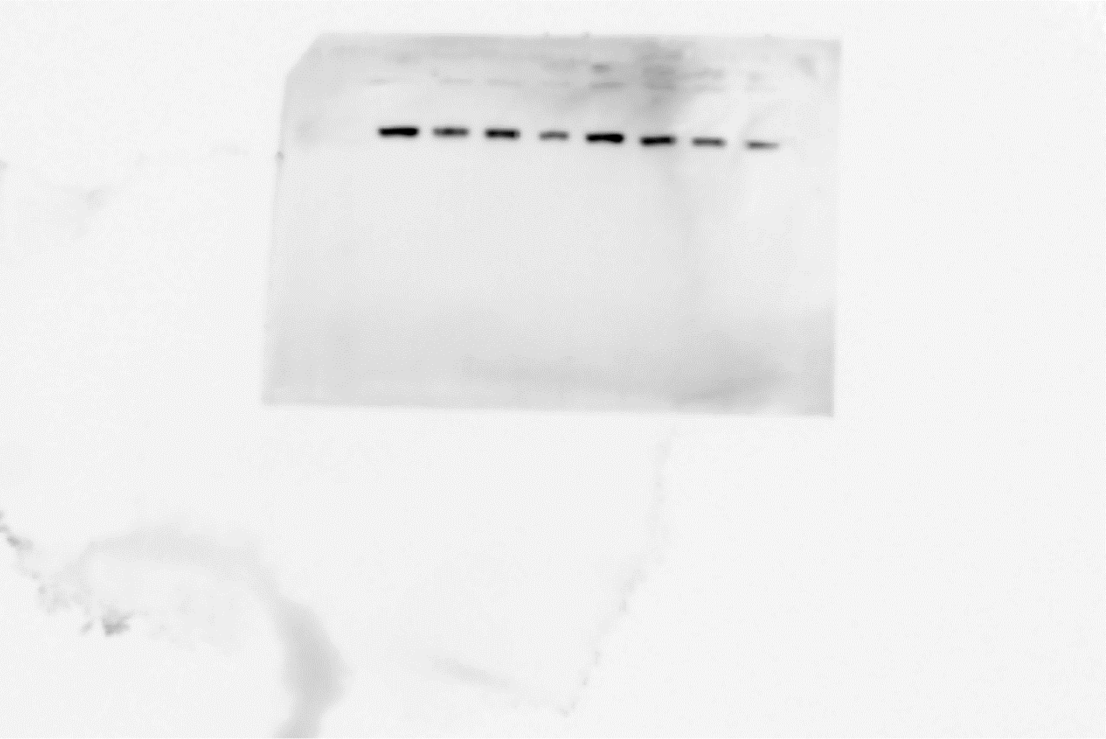

HIF-1α (LPS+Vector/0 2 4 8μM; LPS+HIF1A-OE/0 2 4 8μM)

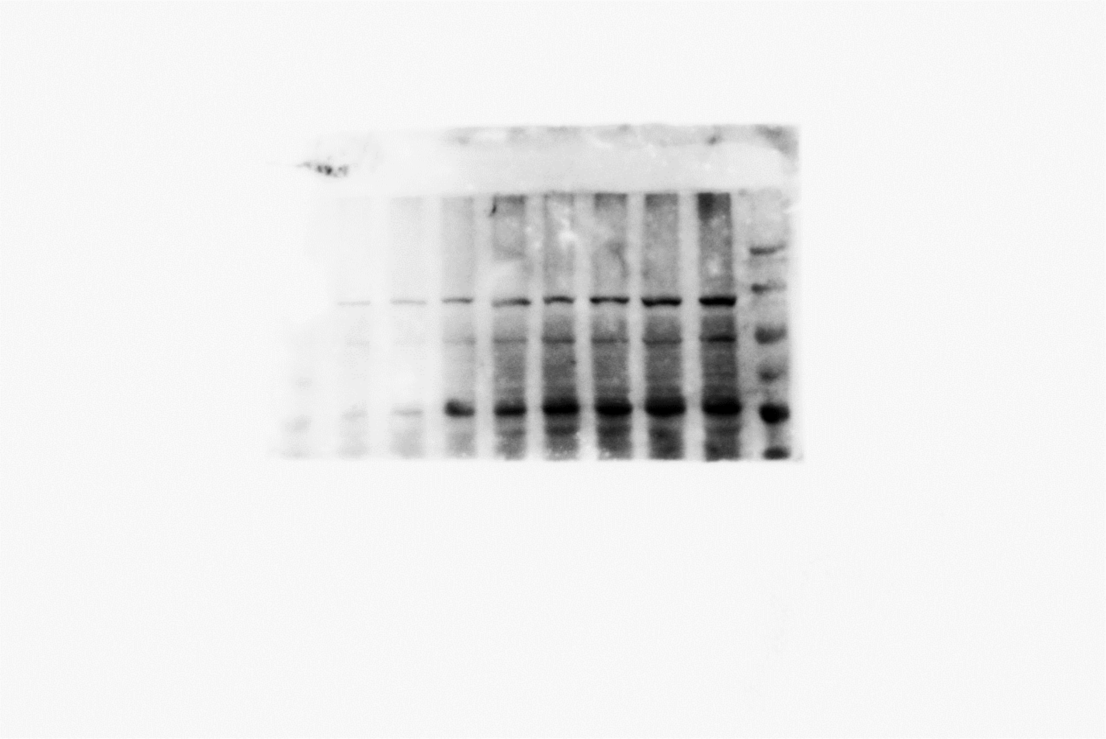

β-actin (LPS+Vector/0 2 4 8μM; LPS+HIF1A-OE/0 2 4 8μM)

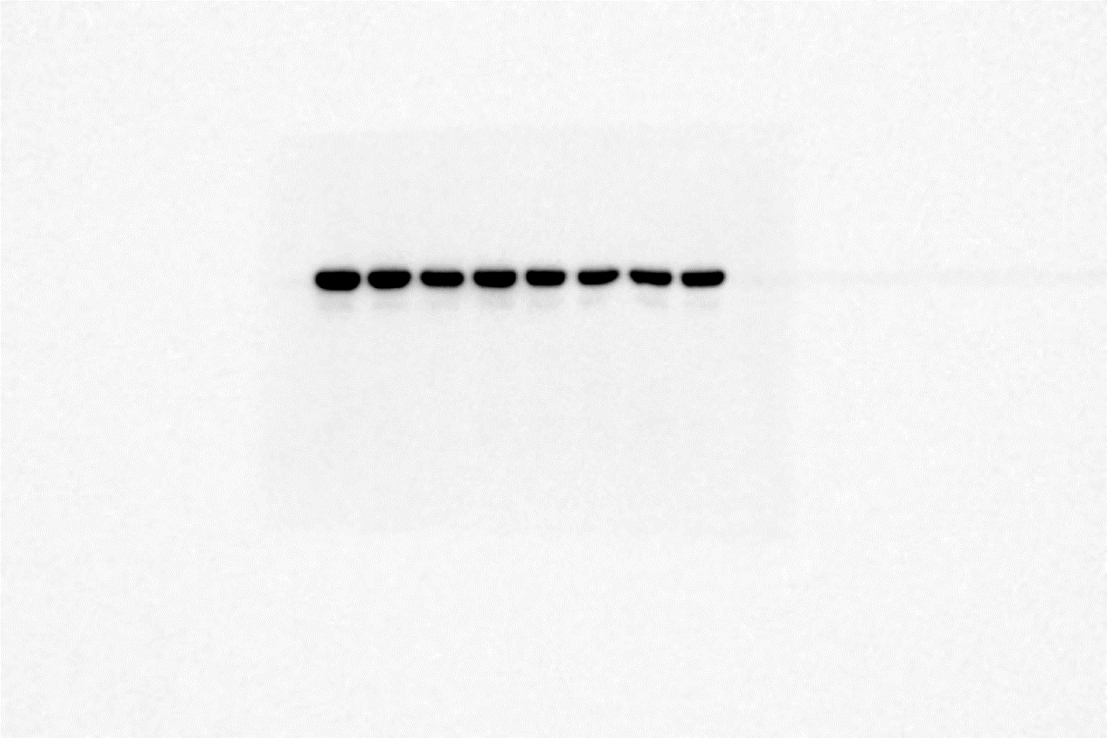

**Figure 5K**

ZO-1 (NC; 3%DSS; 3%DSS+DMOG; 3%DSS+2-ME2)

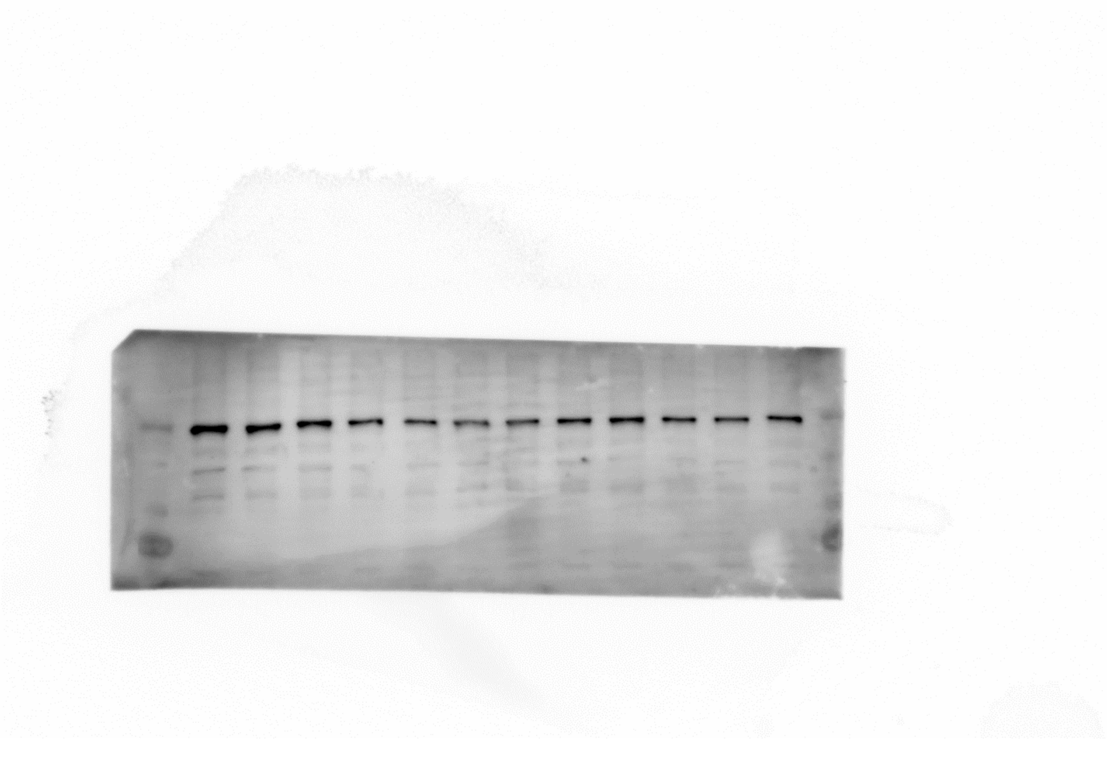

Occludin (NC; 3%DSS; 3%DSS+DMOG; 3%DSS+2-ME2)

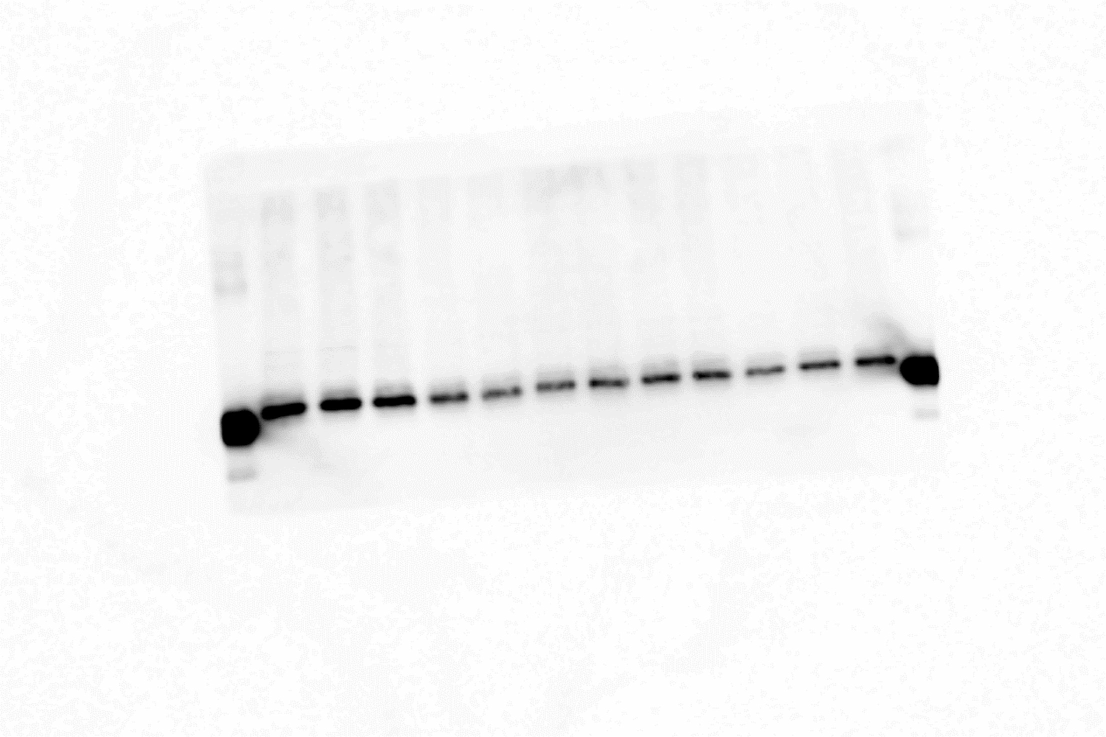

HIF-1α (NC; 3%DSS; 3%DSS+DMOG; 3%DSS+2-ME2)

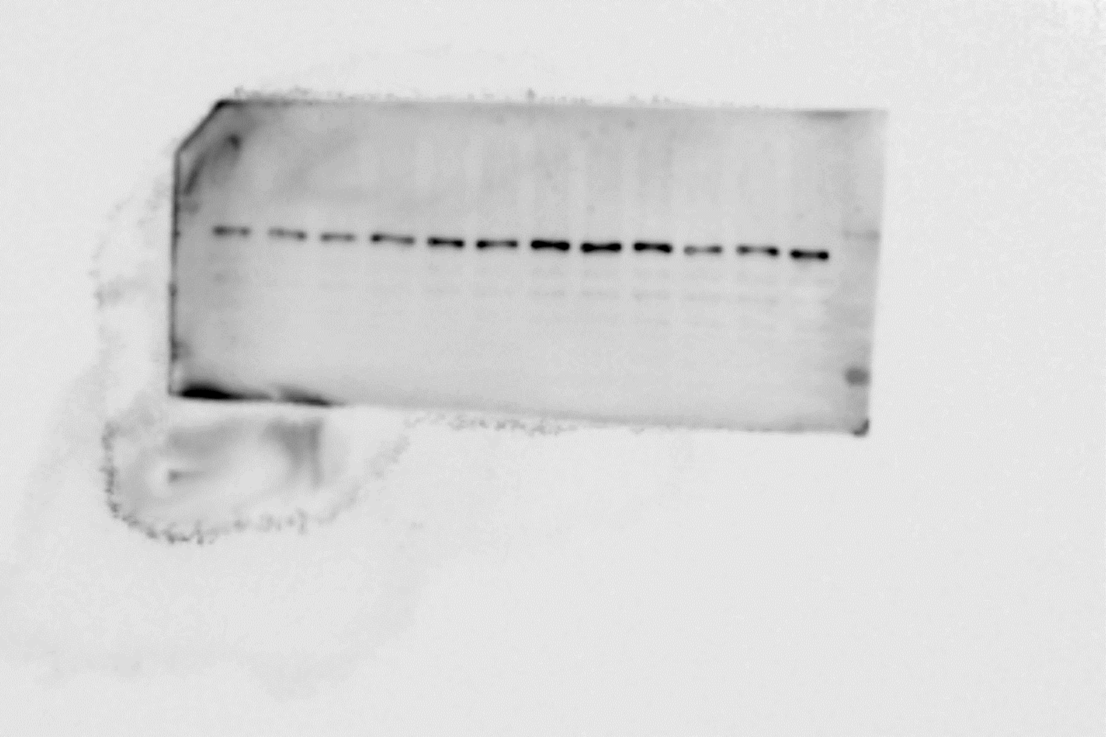

β-actin (NC; 3%DSS; 3%DSS+DMOG; 3%DSS+2-ME2)

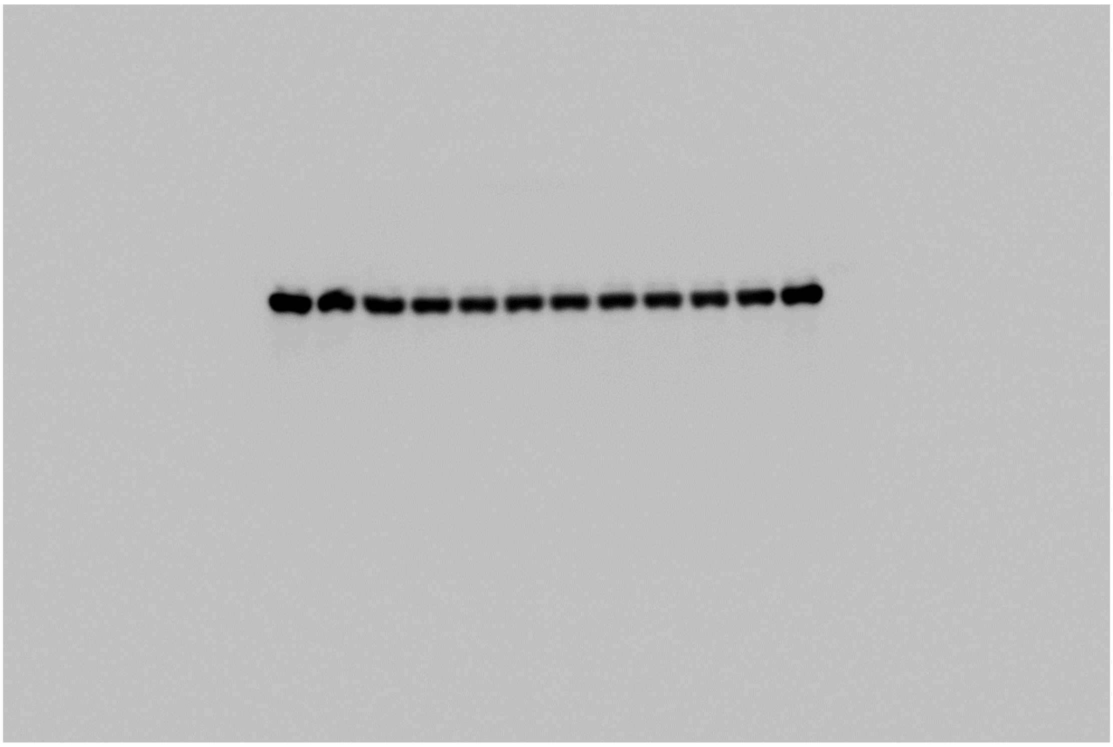

**Figure 6H**

FTL (NC/mouse1-3; 3%DSS/mouse1-3)

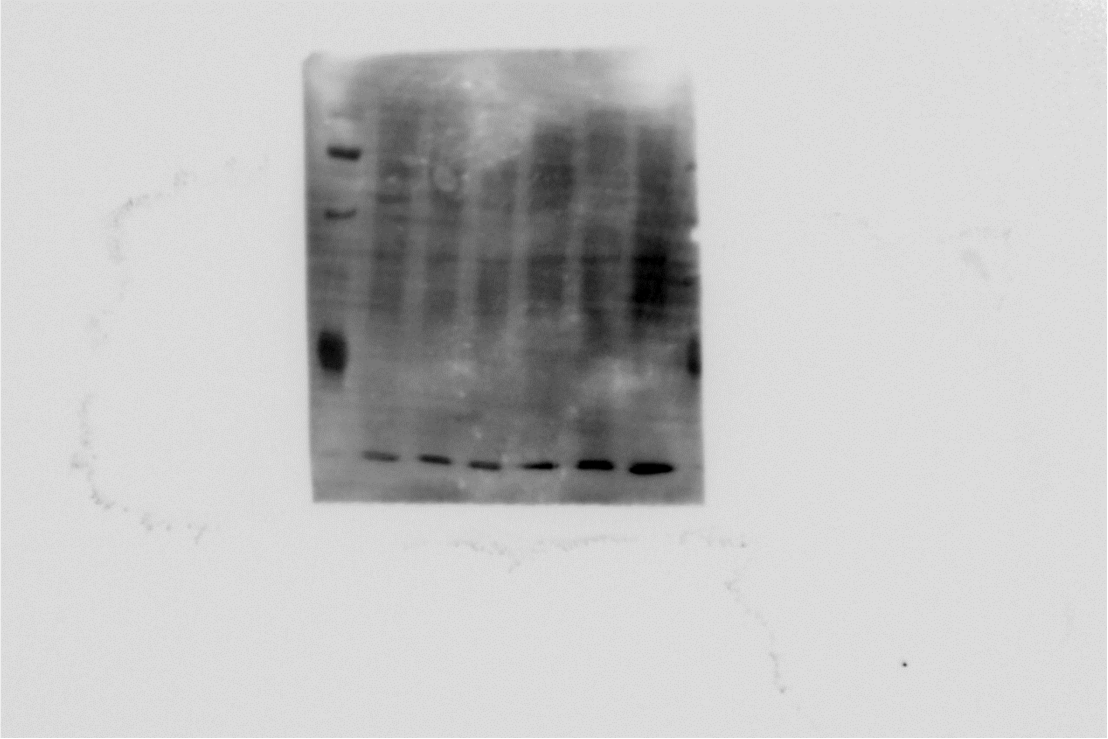

FTH (NC/mouse1-3; 3%DSS/mouse1-3)

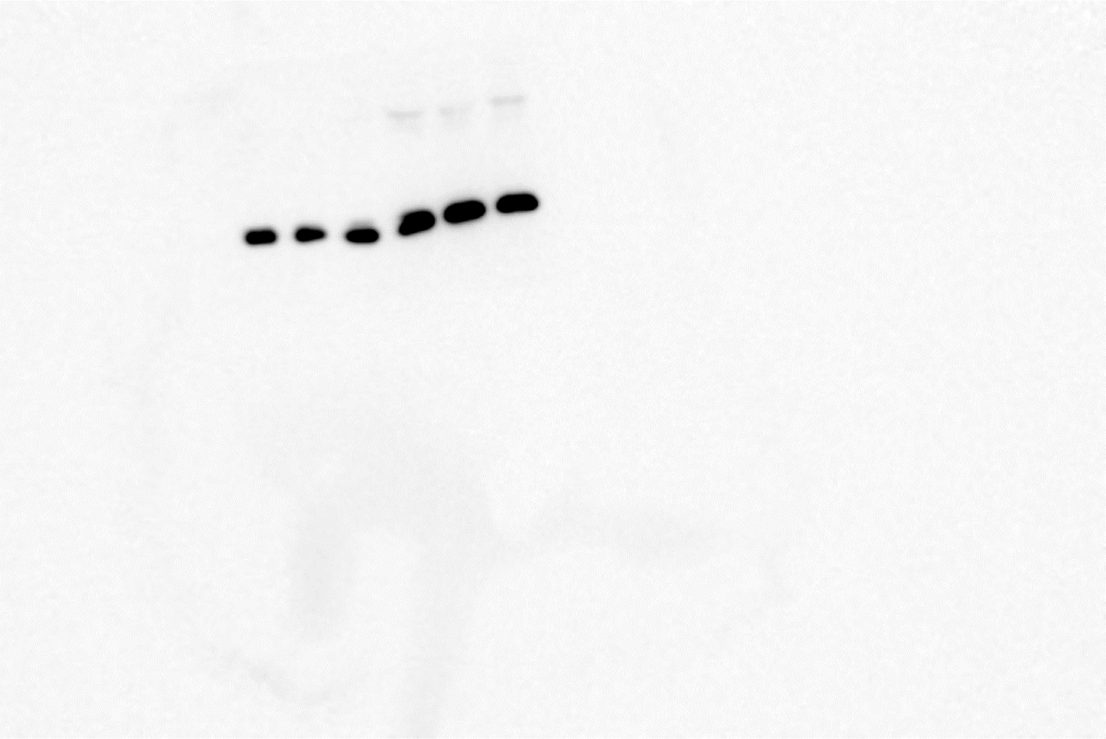

**Figure 6M**

ACSL4 (NC/mouse1-3; 3%DSS/mouse1-3)

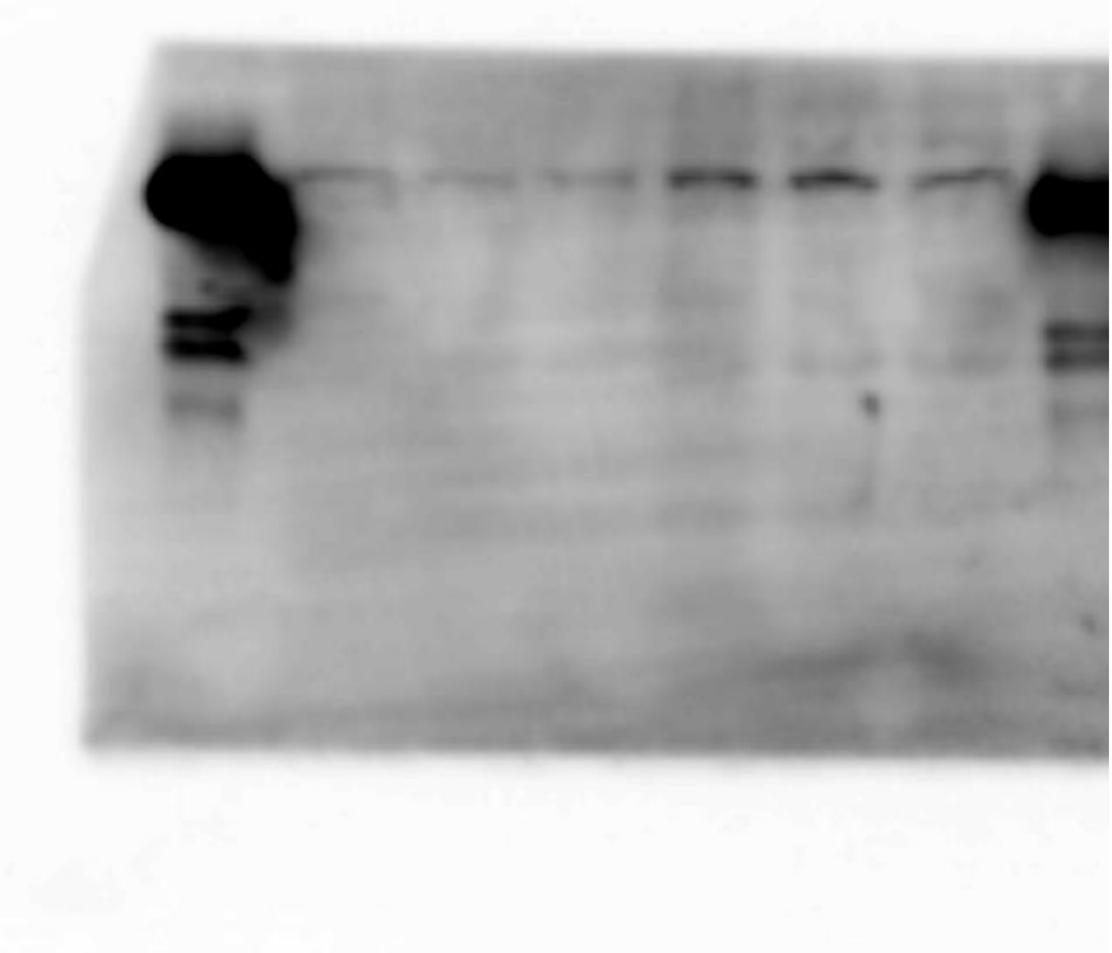

SLC7A11 (NC/mouse1-3; 3%DSS/mouse1-3)

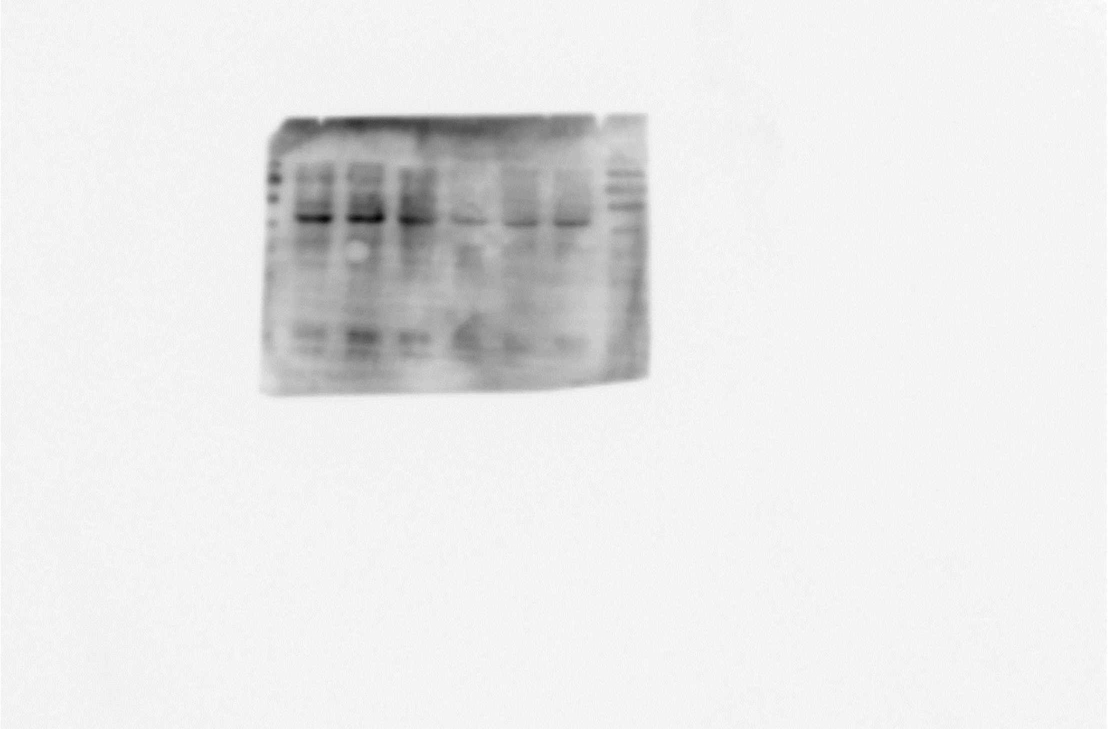

GPX4 (NC/mouse1-3; 3%DSS/mouse1-3)

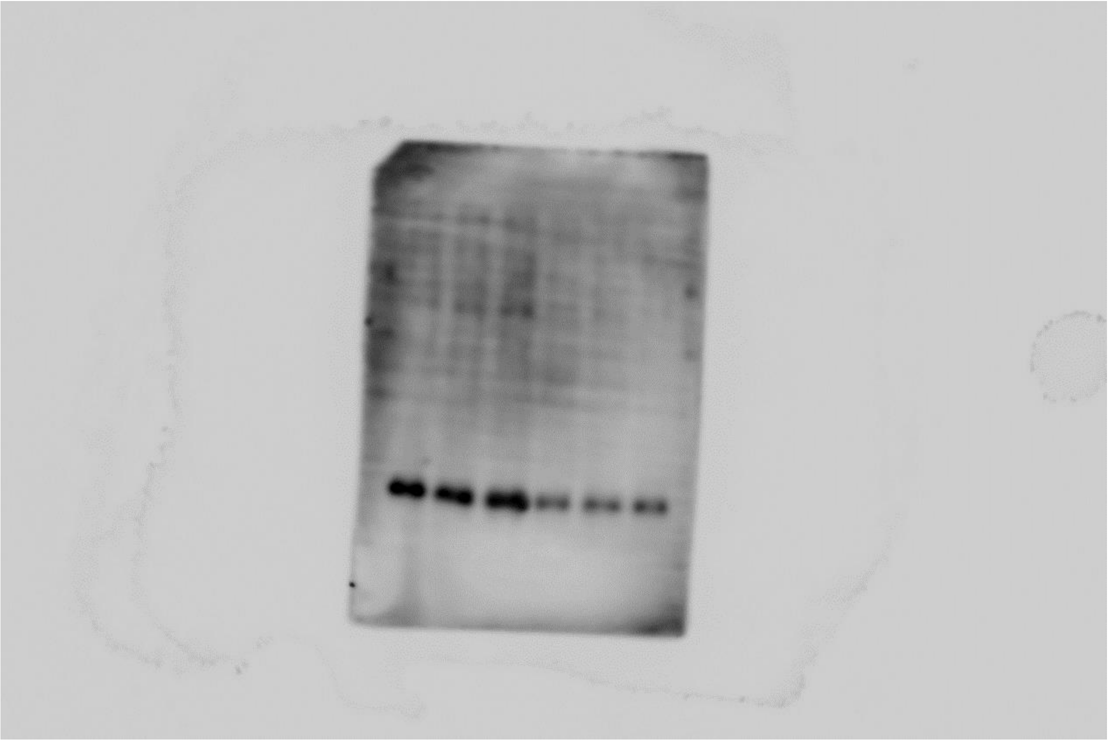

LPCAT3 (NC/mouse1-3; 3%DSS/mouse1-3)

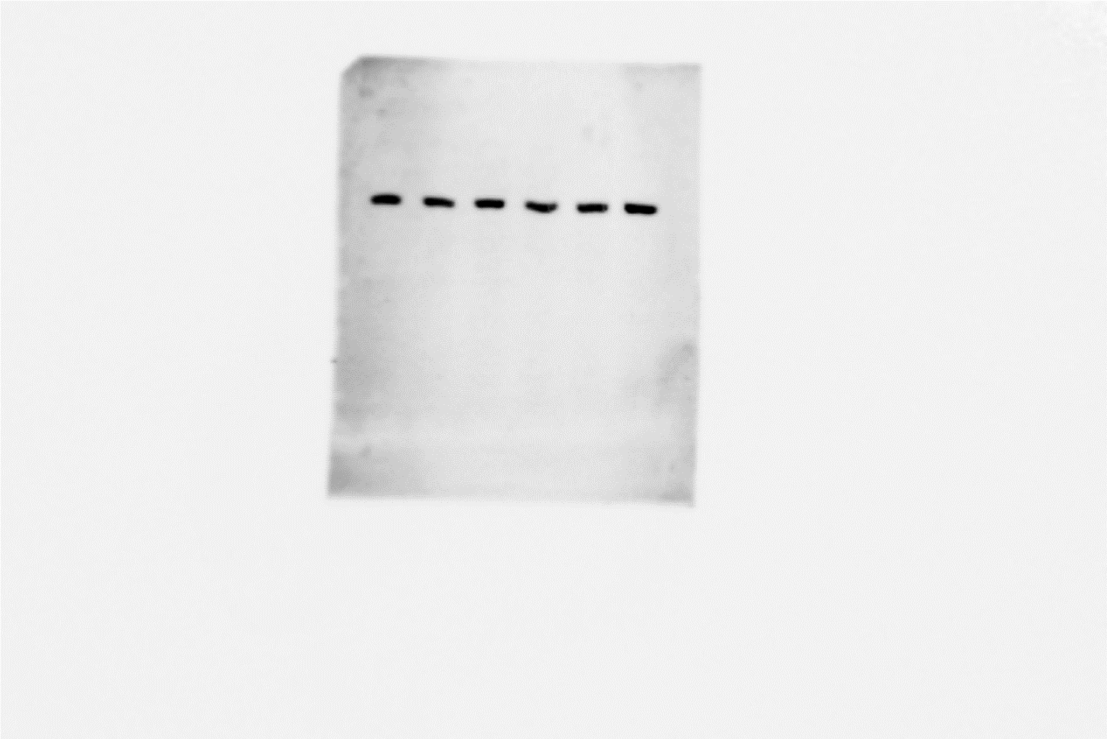

TXNRD1 (NC/mouse1-3; 3%DSS/mouse1-3)

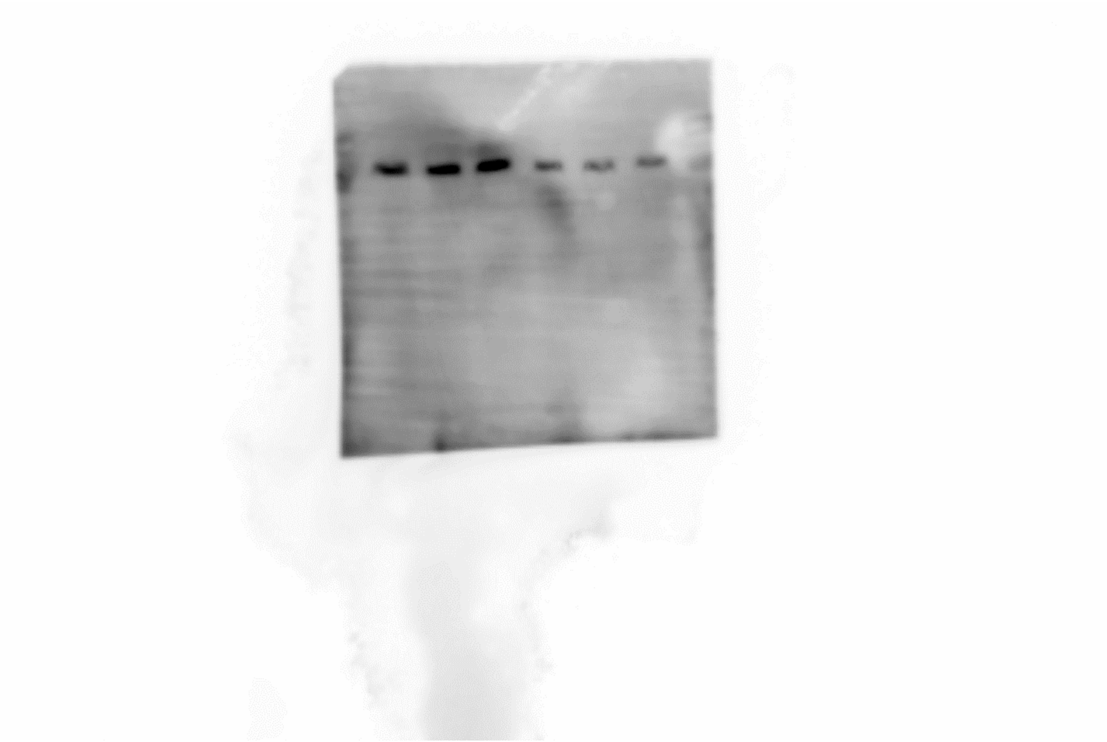

HIF-1α (NC/mouse1-3; 3%DSS/mouse1-3)

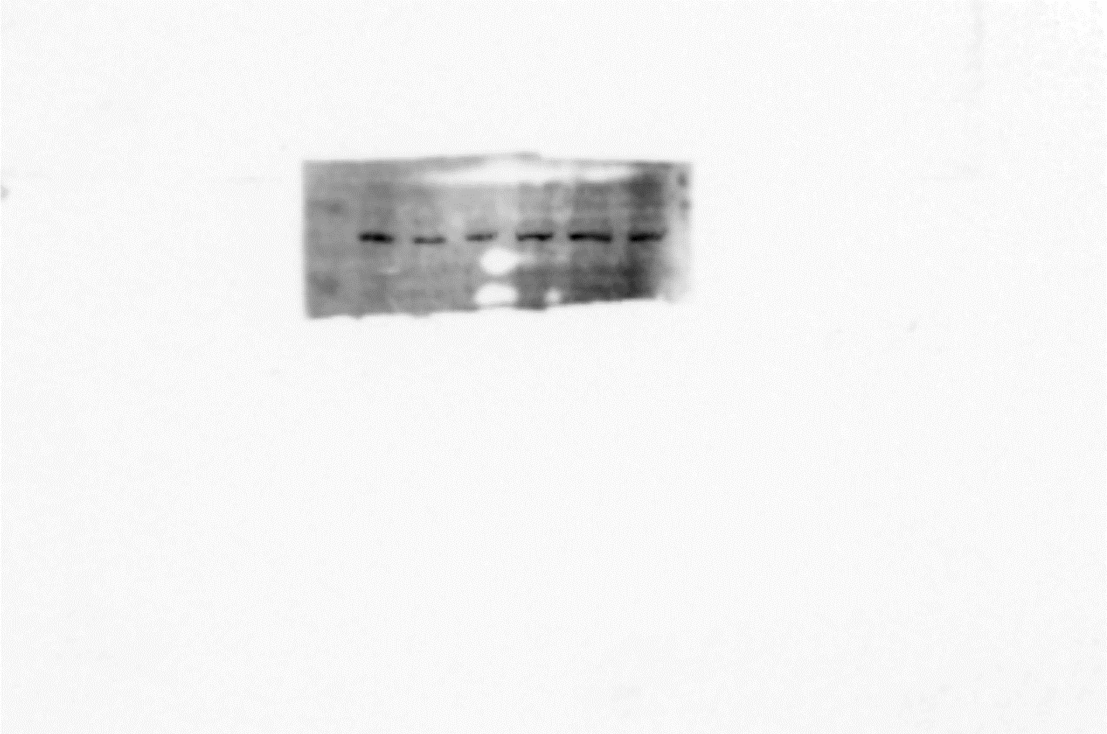

β-actin (NC/mouse1-3; 3%DSS/mouse1-3)

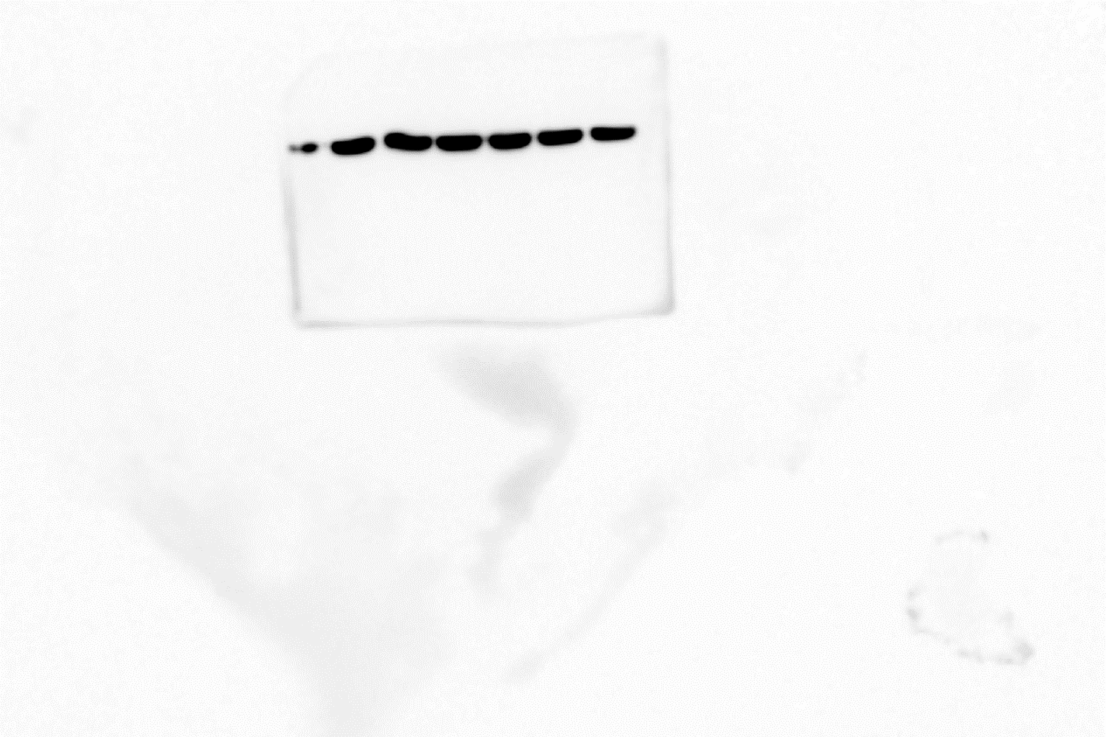

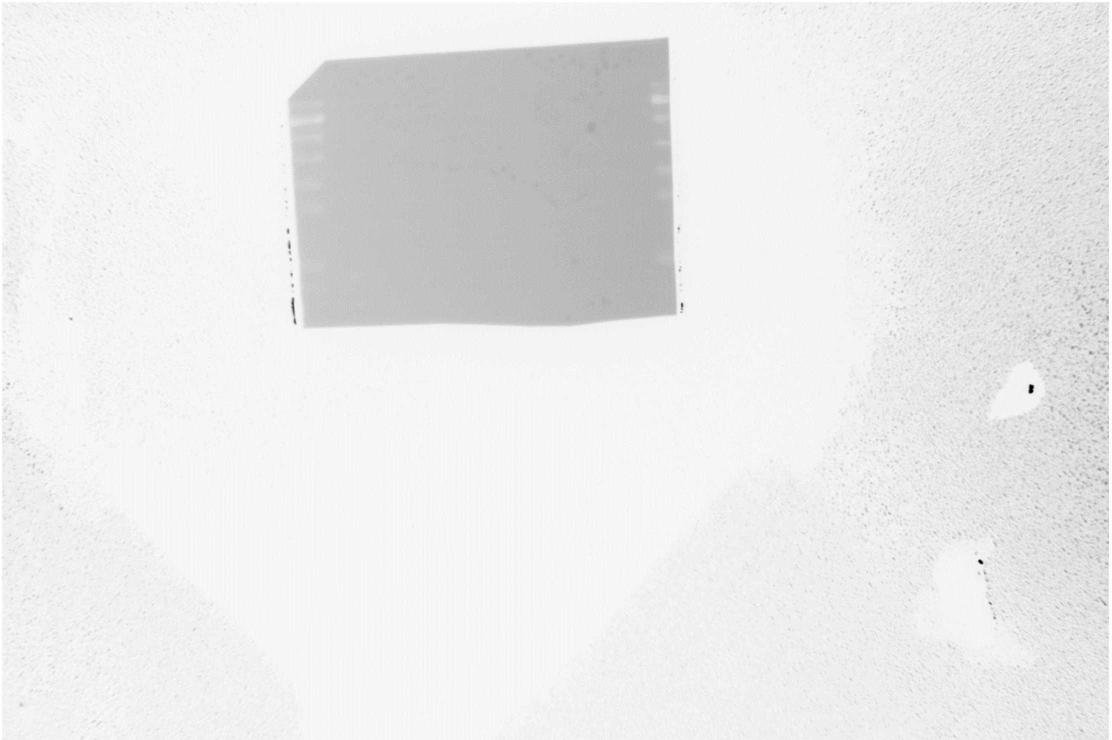

**Figure 7L**

TXNRD1 (3%DSS +RSL3/mouse1-3; 3%DSS+ RSL3+DMOG /mouse1-3; 3%DSS+ RSL3+2-ME2/mouse1-3)

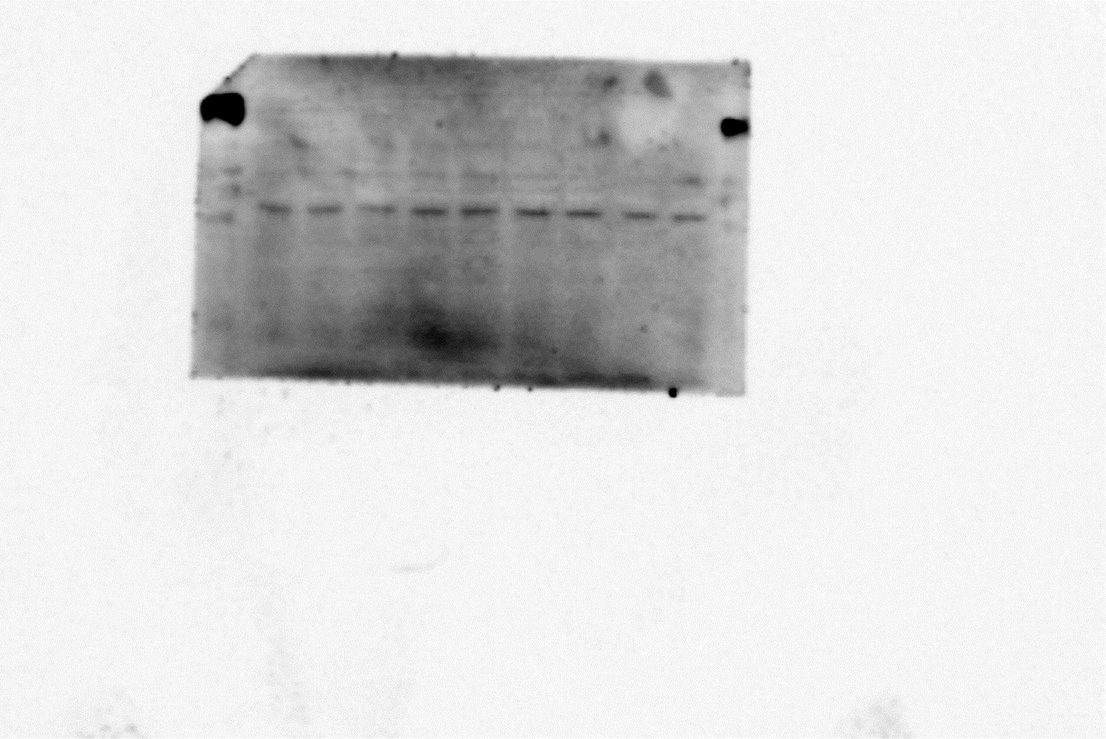

LPCAT3 (3%DSS +RSL3/mouse1-3; 3%DSS+ RSL3+DMOG /mouse1-3; 3%DSS+ RSL3+2-ME2/mouse1-3)

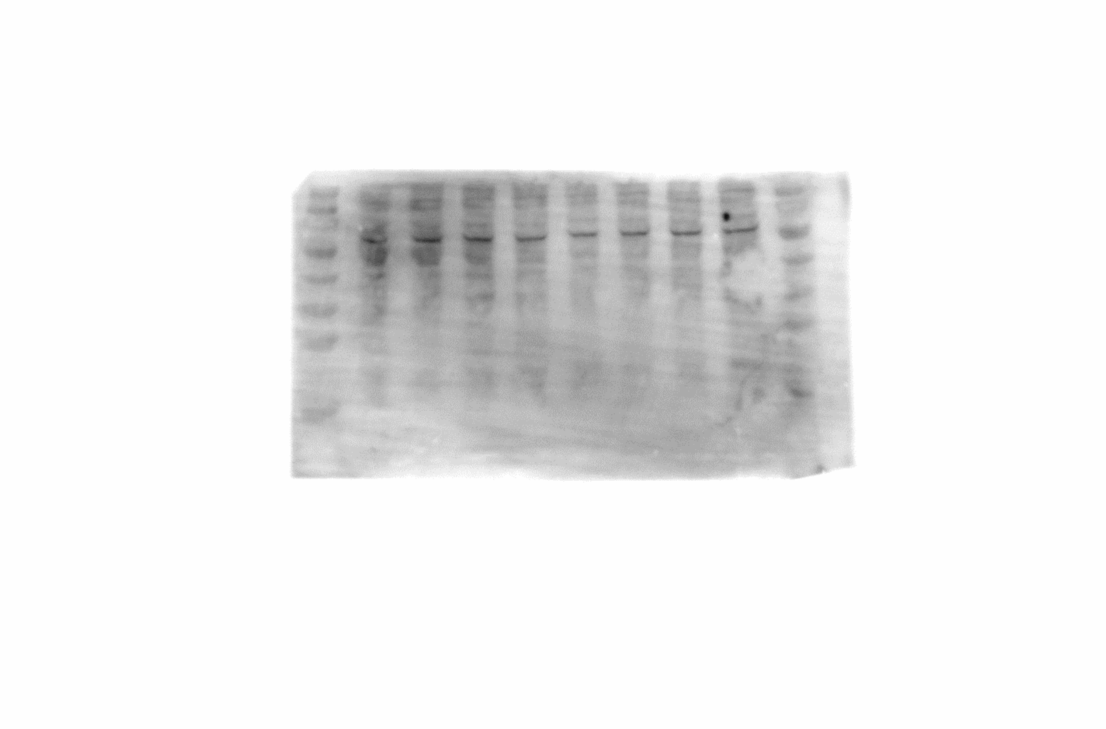

ACSL4 (3%DSS +RSL3/mouse1-3; 3%DSS+ RSL3+DMOG /mouse1-3; 3%DSS+ RSL3+2-ME2/mouse1-3)

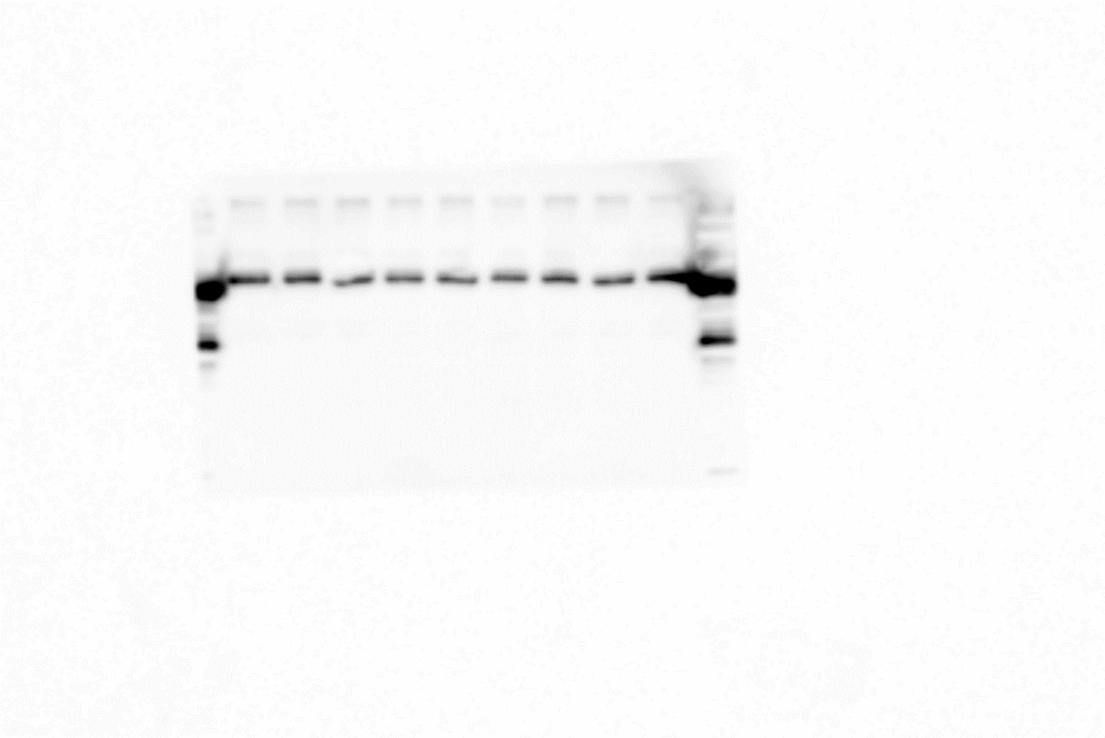

SLC7A11 (3%DSS +RSL3/mouse1-3; 3%DSS+ RSL3+DMOG /mouse1-3; 3%DSS+ RSL3+2-ME2/mouse1-3)

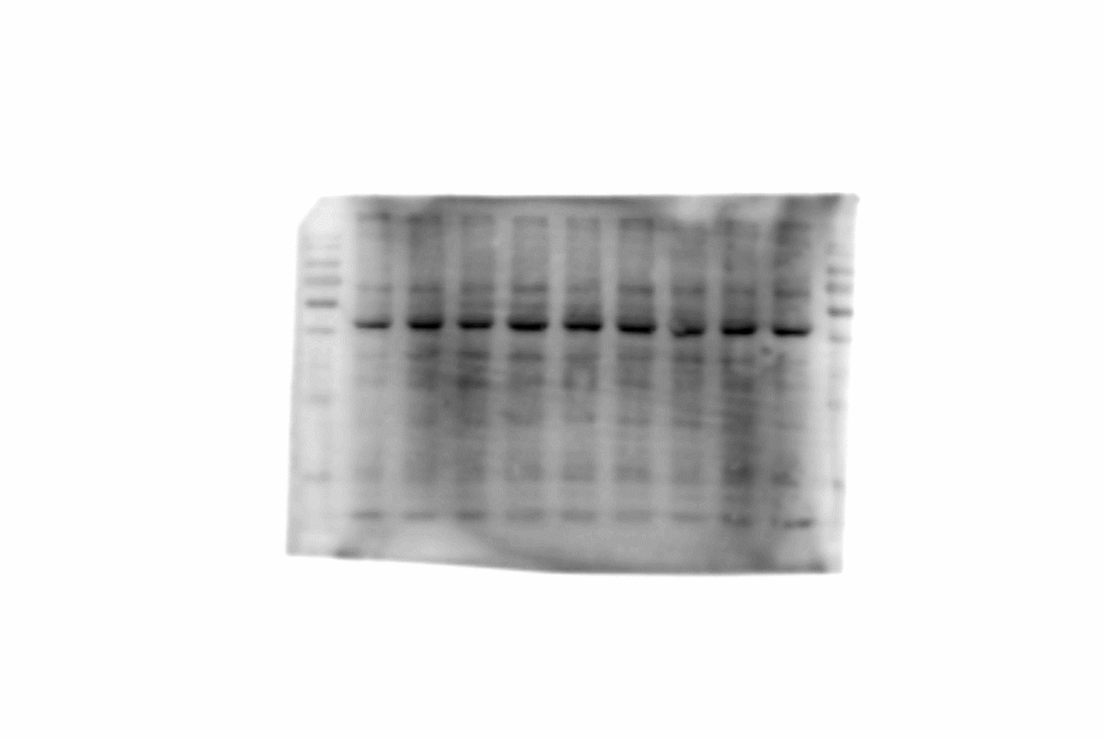

GPX4 (3%DSS +RSL3/mouse1-3; 3%DSS+ RSL3+DMOG /mouse1-3; 3%DSS+ RSL3+2-ME2/mouse1-3)

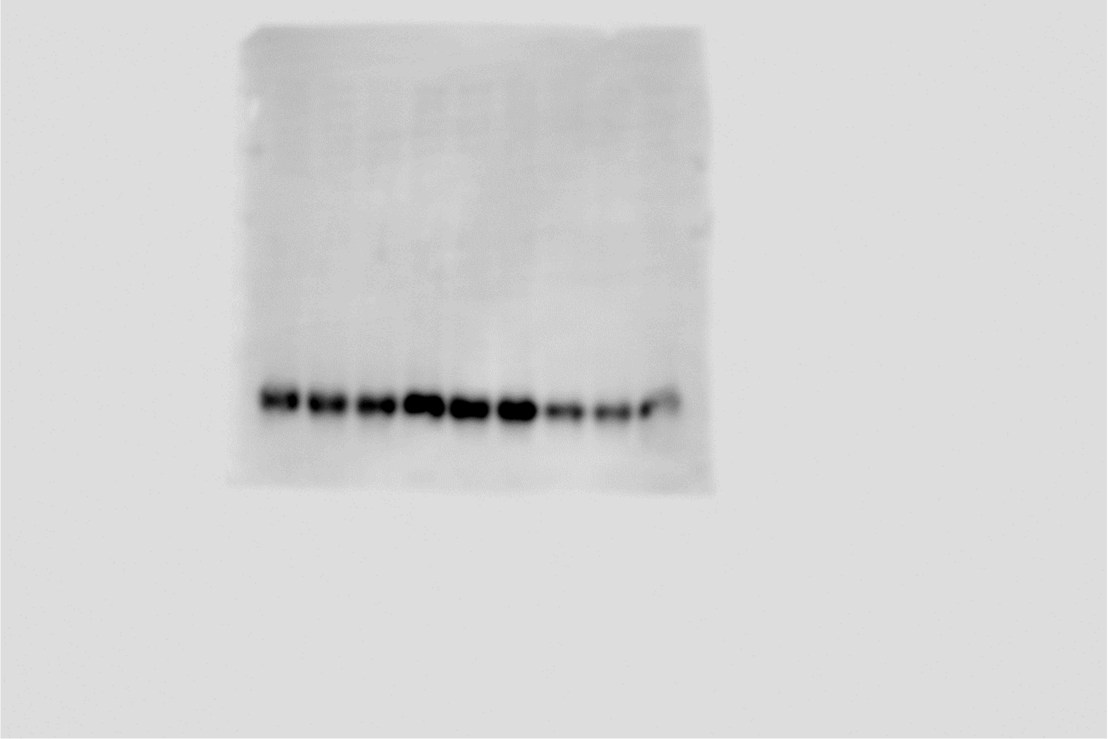

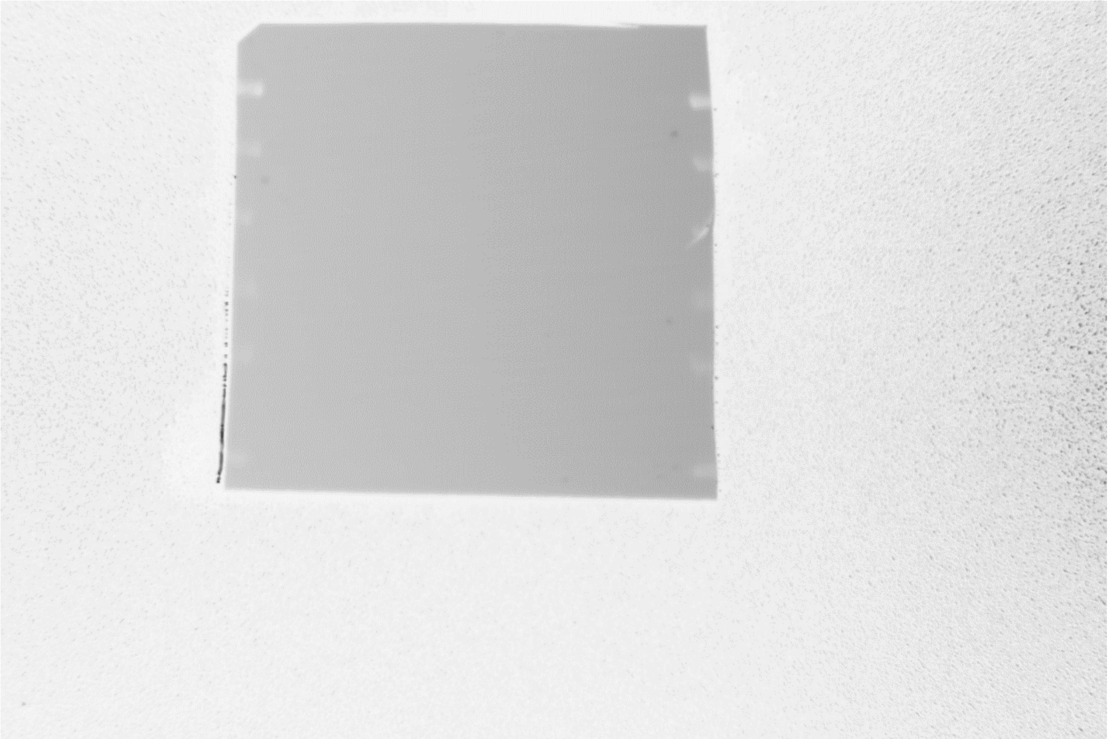

HIF-1α (3%DSS +RSL3/mouse1-3; 3%DSS+ RSL3+DMOG /mouse1-3; 3%DSS+ RSL3+2-ME2/mouse1-3)

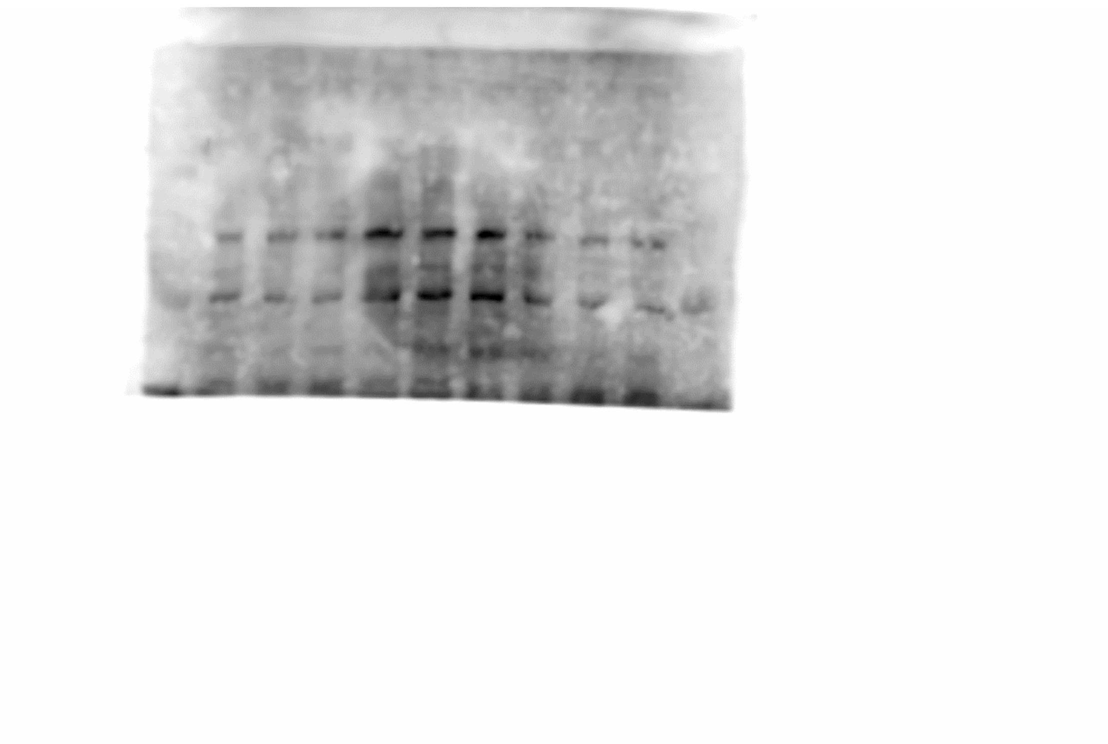

β-actin (3%DSS +RSL3/mouse1-3; 3%DSS+ RSL3+DMOG /mouse1-3; 3%DSS+ RSL3+2-ME2/mouse1-3)

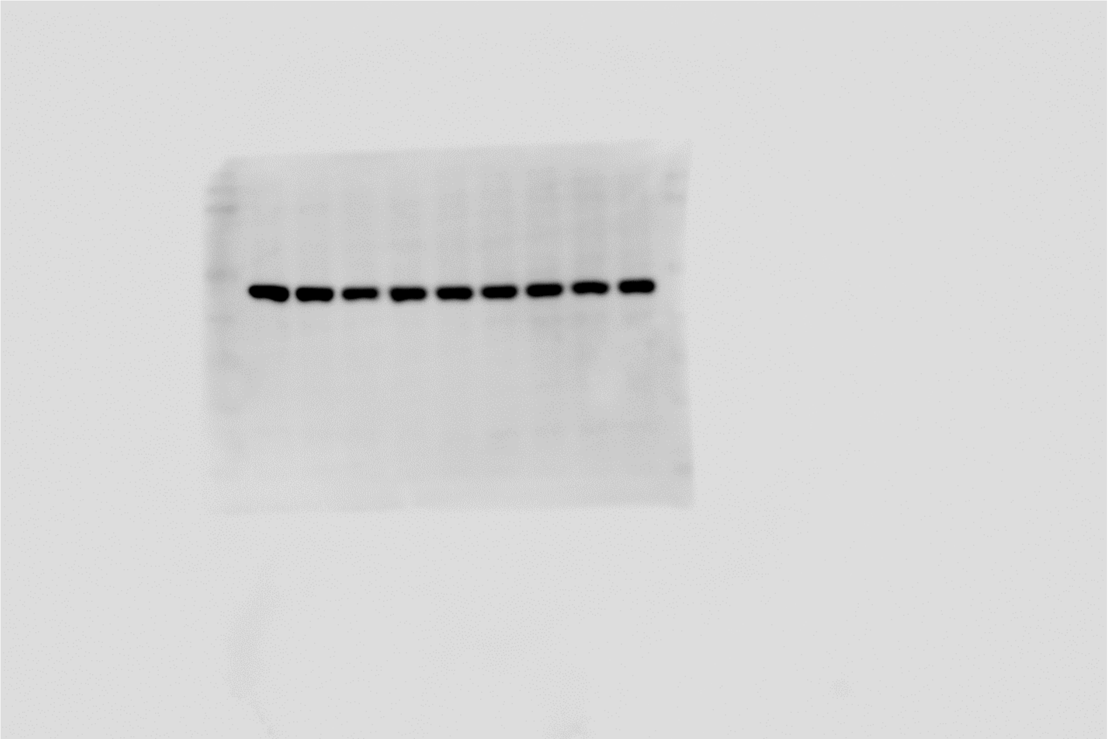

**Figure S4A**

HIF-1α (shHIF1A-1/2/3; shNC; WT)

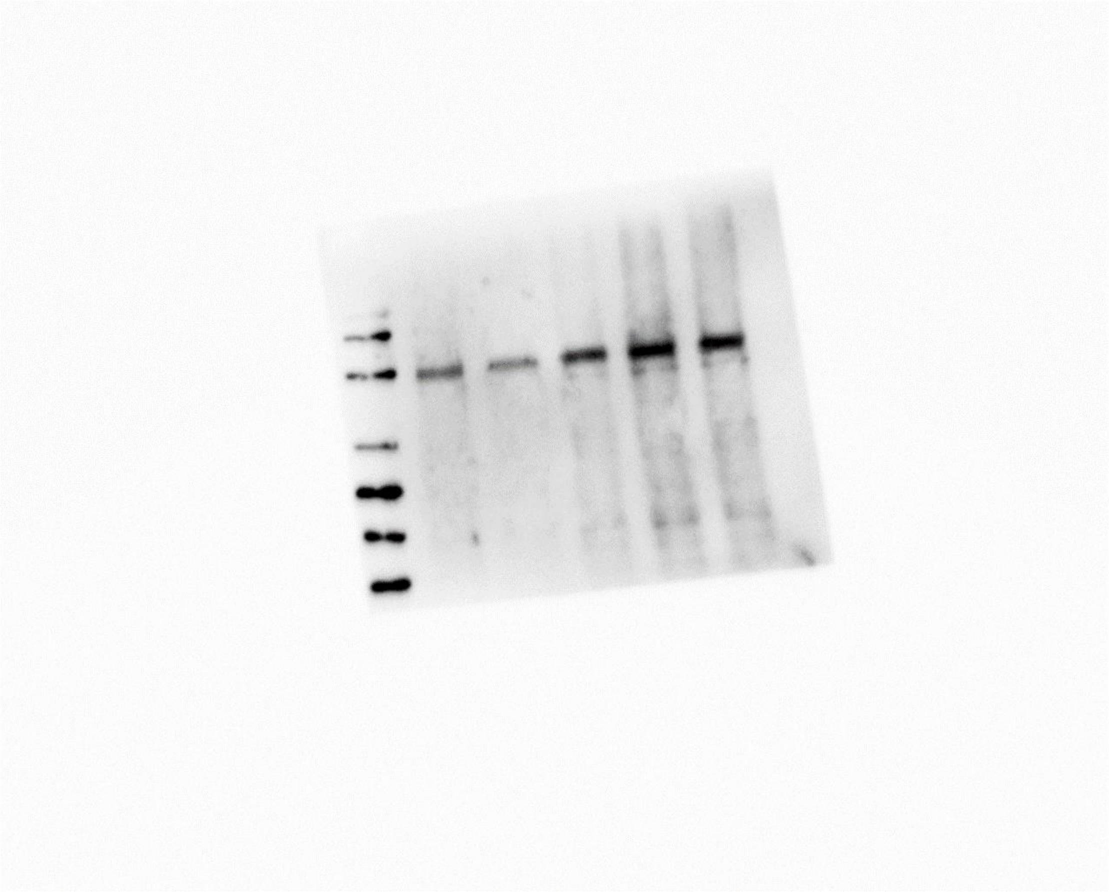

β-actin (shHIF1A-1/2/3; shNC; WT)

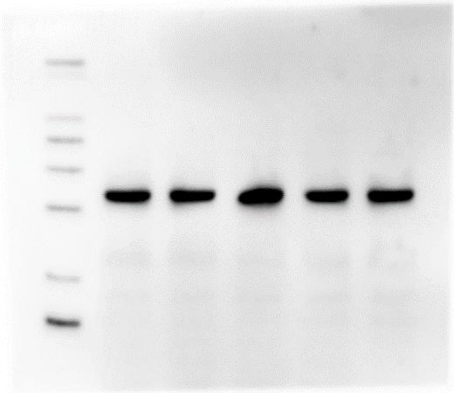

**Figure S4I**

ACSL4 (shNC /LPS- +; shHIF1A/ LPS- +)

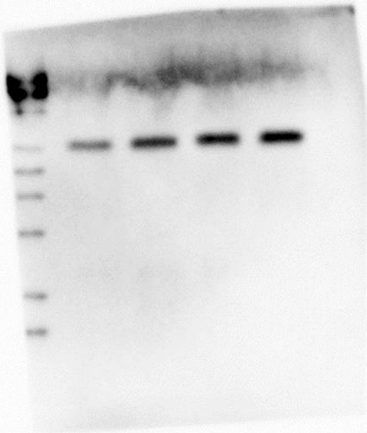

LPCAT3 (shNC /LPS- +; shHIF1A/ LPS- +)

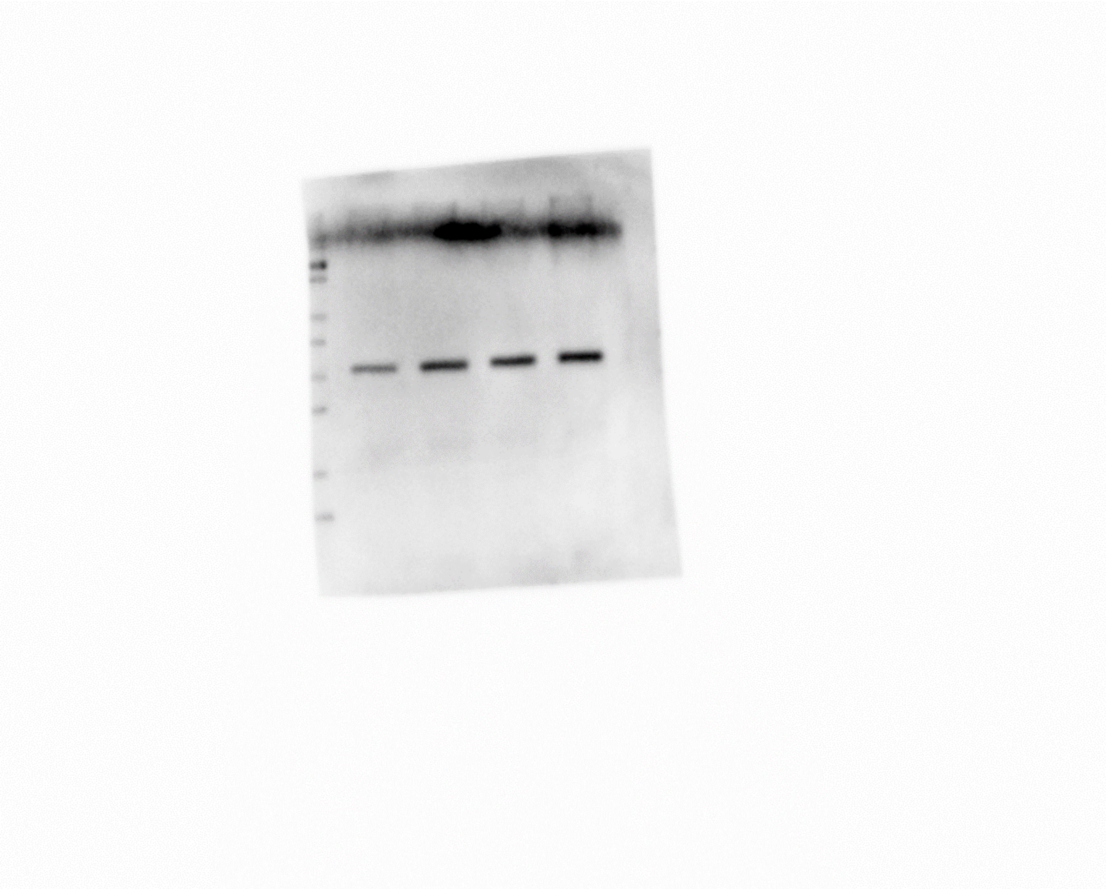

SLC7A11 (shNC /LPS- +; shHIF1A/ LPS- +)

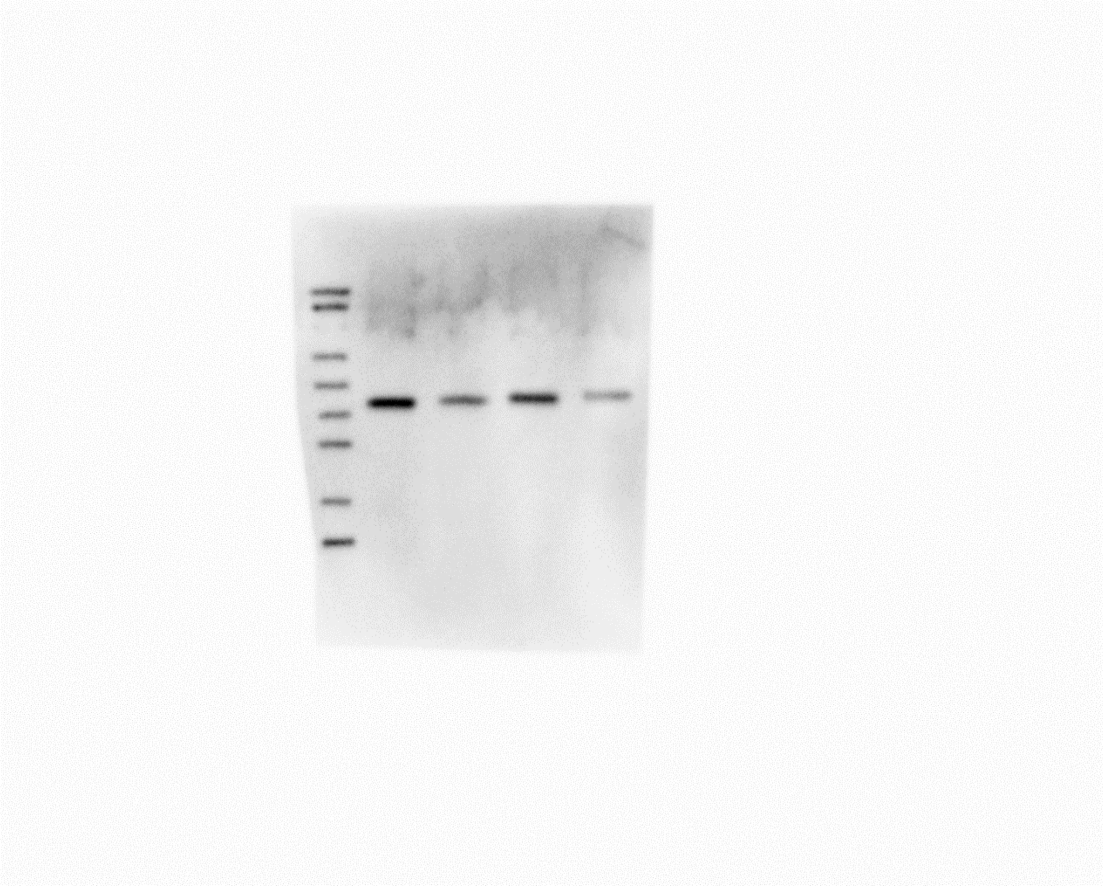

GPX4 (shNC /LPS- +; shHIF1A/ LPS- +)

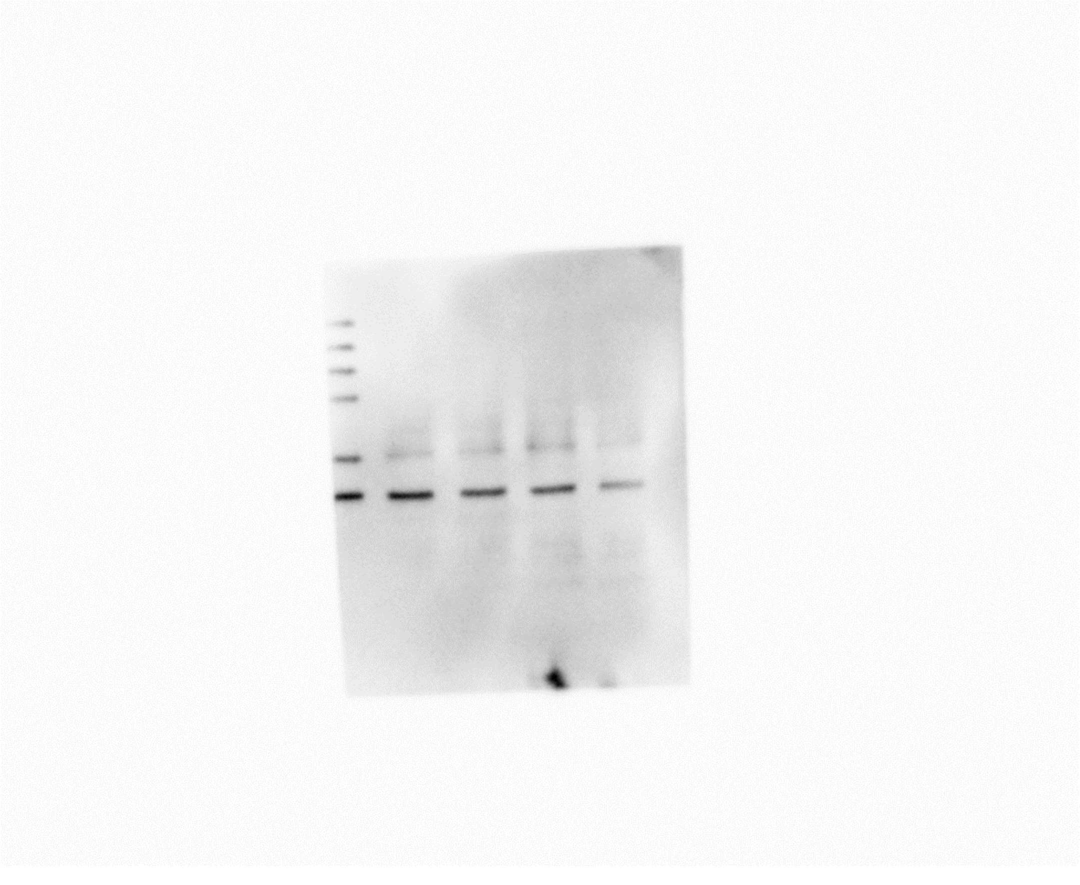

TXNRD1 (shNC /LPS- +; shHIF1A/ LPS- +)

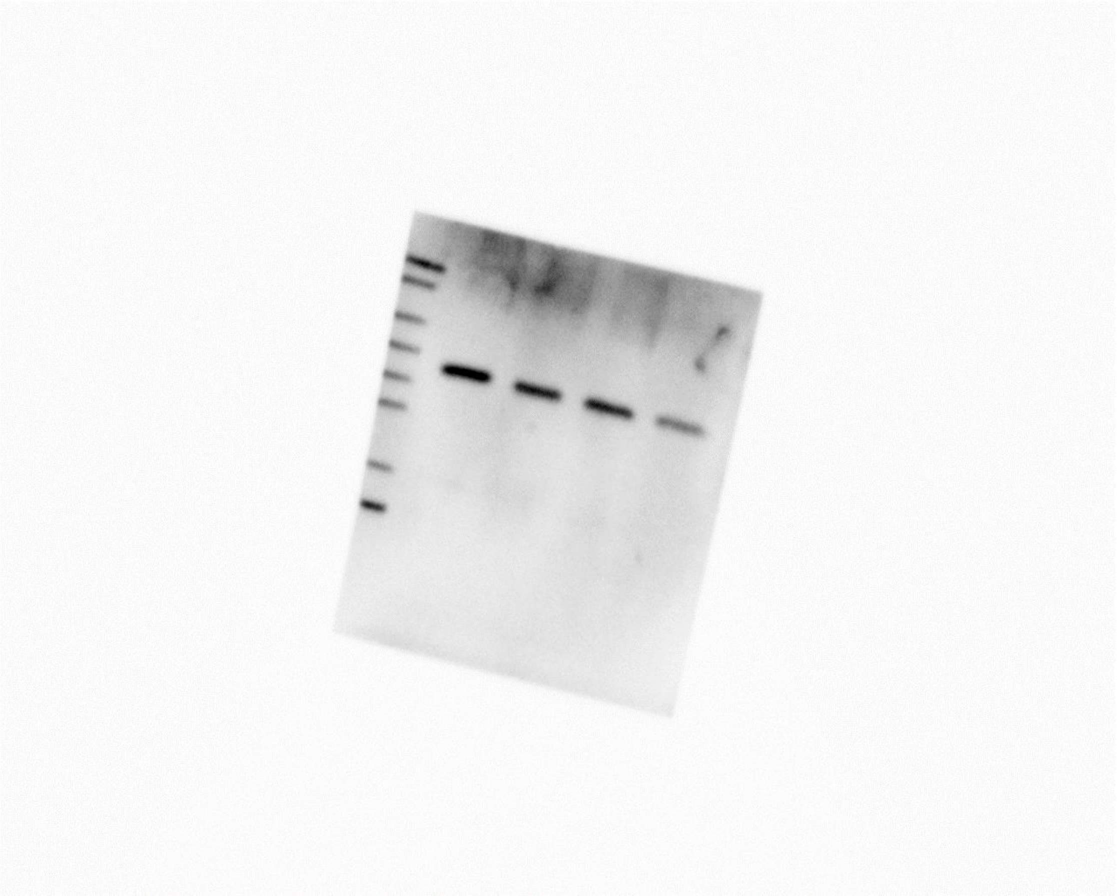

FTL (shNC /LPS- +; shHIF1A/ LPS- +)

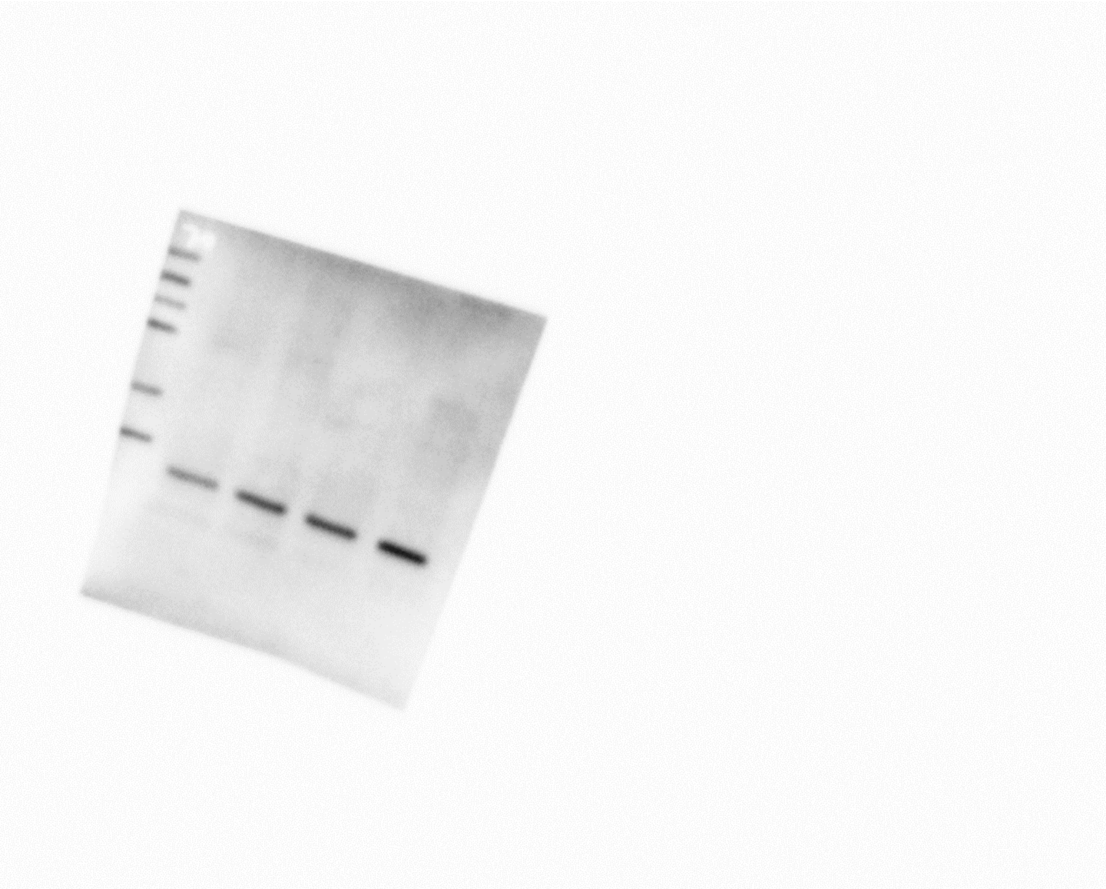

FTH (shNC /LPS- +; shHIF1A/ LPS- +)

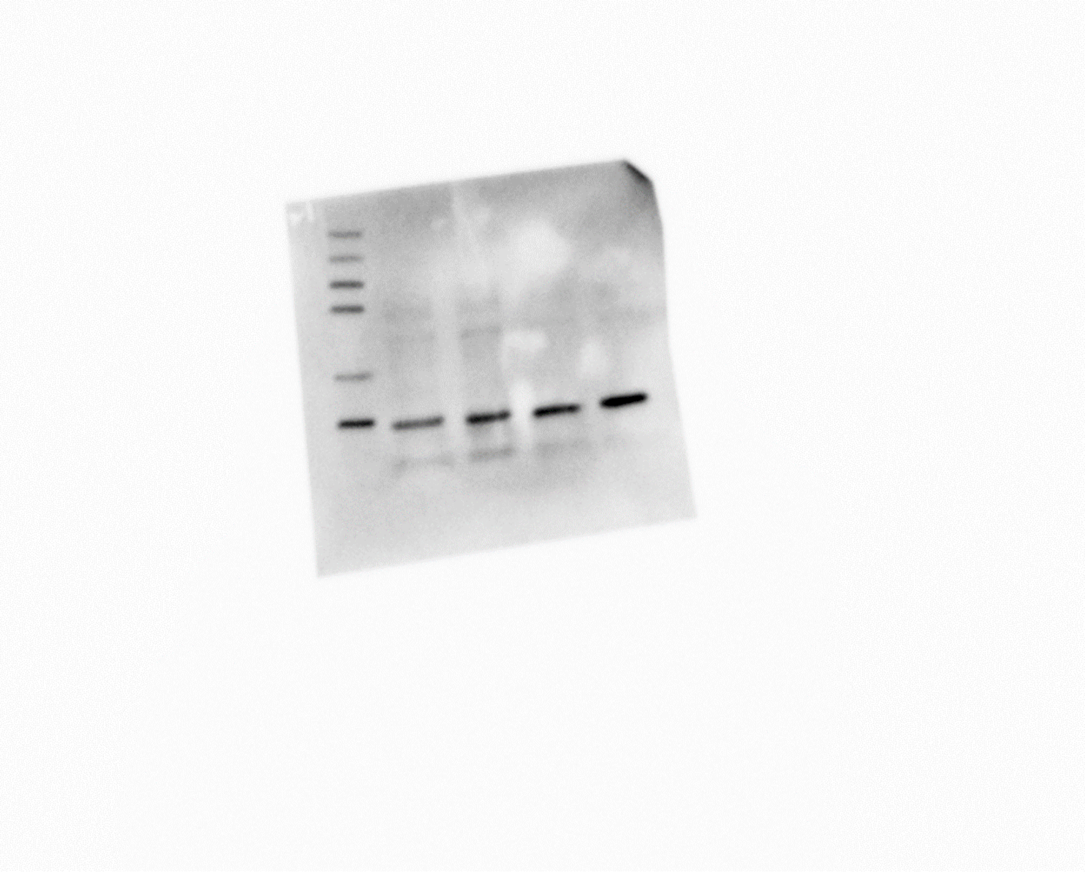

HIF-1α (shNC /LPS- +; shHIF1A/ LPS- +)

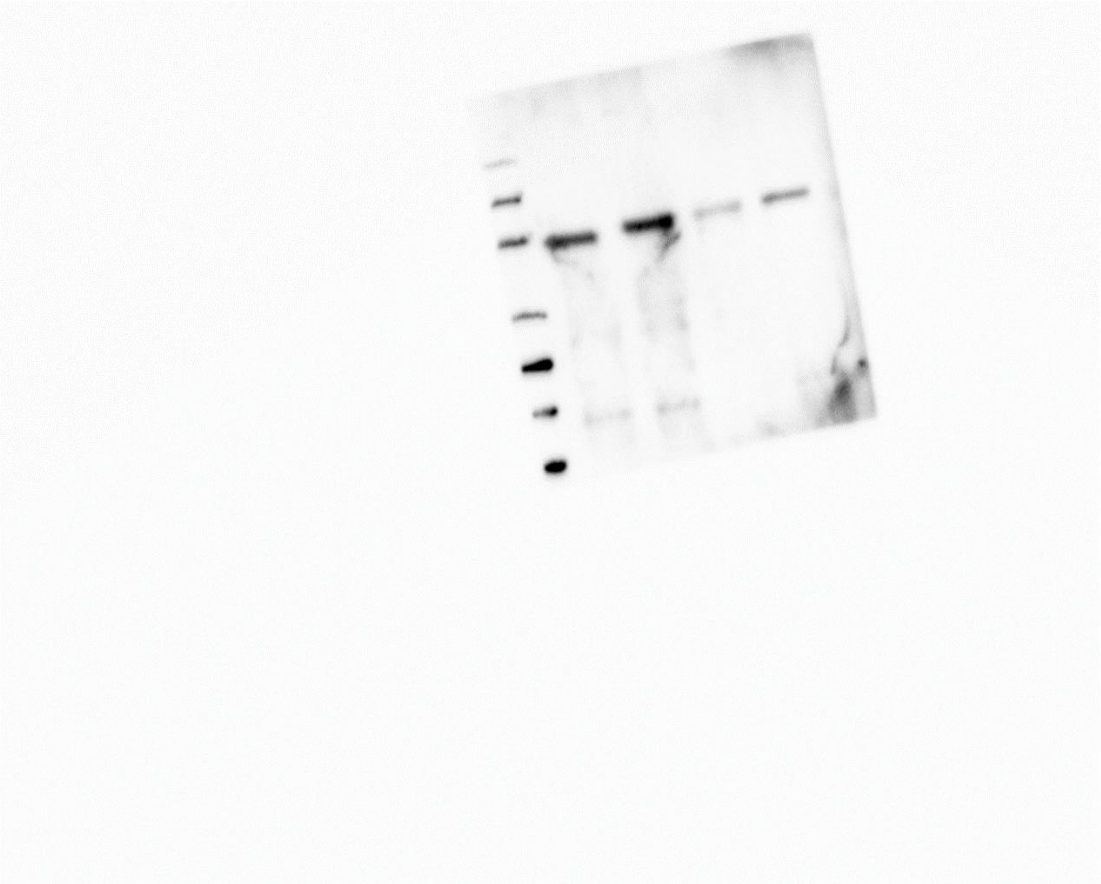

β-actin (shNC /LPS- +; shHIF1A/ LPS- +)

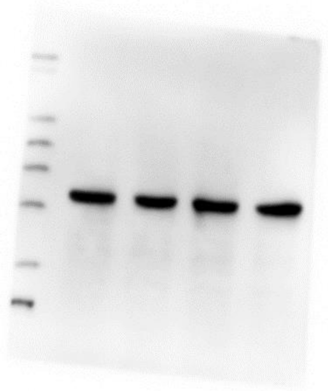

Supplement: Supplementary file 2 — Western blot originai image [file 41419_2025_7883_MOESM2_ESM.pdf]
